# Tracing Pharmaceuticals in Water Systems: Focus on Neurodegenerative and Psychiatric Treatments

**DOI:** 10.3390/jox14040096

**Published:** 2024-11-21

**Authors:** Paula Paíga, Cristina Delerue-Matos

**Affiliations:** REQUIMTE/LAQV, Instituto Superior de Engenharia do Porto, Instituto Politécnico do Porto, Rua Dr. António Bernardino de Almeida, 431, 4249-015 Porto, Portugal

**Keywords:** aquatic ecosystems, environmental and public health, pharmaceuticals (neurodegenerative diseases and psychiatric disorders), caffeine, surface water, wastewaters

## Abstract

Pharmaceutical residues in aquatic ecosystems pose significant environmental and public health challenges. Identifying the presence and levels of these pharmaceuticals is crucial. This study developed an analytical method to detect pharmaceuticals used for Alzheimer’s (AD) and Parkinson’s (PD) disease, including psychiatric drugs and the stimulant caffeine, targeting 30 compounds. Optimized mass spectrometric and liquid chromatographic parameters enabled robust detection and quantification. The methodology was applied to 25 surface and wastewater samples. Twenty-one compounds were detected including eight psychiatric drugs, five metabolites (citalopram N-oxide, citalopram propionic acid, desmethylcitalopram, *O*-desmethylvenlafaxine, and 10,11-epoxycarbamazepine), and seven AD/PD pharmaceuticals along with caffeine. Nine compounds (apomorphine, benserazide, donepezil, didemethylcitalopram, carbidopa, norfluoxetine, galantamine, pramipexole, and safinamide) were not detected. Fluoxetine was found in all samples, and caffeine had the highest concentration at 76,991 ng/L, reflecting its high consumption. Concentrations ranged from 29.8 to 656 ng/L for caffeine, <MDL to 381 ng/L for psychiatric drugs, and <MDL to 37.1 ng/L for AD and PD pharmaceuticals in surface water. In wastewater, concentrations ranged from 140 to 76,991 ng/L for caffeine, <MDL to 5227 ng/L for psychiatric drugs, and <MDL to 206 ng/L for AD and PD pharmaceuticals. These findings highlight the critical need for comprehensive environmental monitoring.

## 1. Introduction

While the undeniable health benefits of pharmaceuticals are acknowledged, their presence as contaminants in the environment has emerged as a pressing concern [1]. These compounds can enter the environment through various pathways, including wastewater discharges and improper disposal practices. Pharmaceutical residues have been detected in various environmental samples, namely rivers, lakes and streams [2,3,4,5,6,7,8], oceans [9,10], drinking water [11], and soils and sediments [12]. Understanding the occurrence and fate of pharmaceuticals in water bodies is crucial for assessing environmental risks. The scientific community has given more attention to evaluating the environmental impact of pharmaceutical compounds from the non-steroid anti-inflammatory drugs, analgesics, and psychiatric drugs due to their high consumption worldwide [9,13,14,15,16,17,18,19,20,21,22]. However, monitoring other therapeutic classes in the environment is of utmost importance for identifying which pharmaceutical compounds are present and at what levels.

Nowadays, a theme making headlines in the media is the increase in life expectancy. With 8 billion individuals inhabiting Earth [23], the prevailing demographic landscape showcases a rise in the average life expectancy, a decrease in birth rates, and a growing population of older adults and senior citizens [24]. In 2020, the global population of individuals aged 60 or over was approximately 1 billion, projected to reach 1.4 billion by 2030 and 2.1 billion by 2050 [25]. Although some elderly individuals remain in good health, others are at risk of mental health issues like depression and anxiety, as well as reduced mobility, chronic pain, or other health problems [25]. Statistics reveal that about 14% of adults aged 60 and above develop a mental disorder [25]. Alzheimer’s (AD) and Parkinson’s (PD) disease are the most common neurodegenerative diseases among the elderly [15]. AD, a progressive, disabling, and irreversible disease, is a major form of dementia in this population. PD is a chronic and progressive neurological disorder that affects movement [26]. In addition to treatments for these diseases, psychiatric drugs are also commonly administered.

Monitoring studies significantly contributes to the broader understanding of pharmaceutical pollution and its impact on ecosystems and public health. Due to their widespread use and potential environmental persistence, psychiatric drugs are a major focus of environmental monitoring studies, with the scientific community often analyzing multiple pharmaceuticals from this class [3,17,18,19,27,28,29,30,31]. On the other hand, pharmaceuticals used for degenerative diseases are typically limited to a few AD and PD compounds on the list of monitored substances. Studies on this topic often focus on just one or a small selection of these compounds for environmental monitoring [32,33,34,35,36,37,38,39,40,41,42,43,44,45,46,47,48,49,50,51,52,53,54,55,56].

This study focuses on pharmaceuticals used in AD and PD disease due to their increasing use in the aging population and the limited research on their environmental impact. Psychiatric drugs, often co-administered with AD and PD treatments, are included due to their high consumption and frequent detection in environmental samples. Together, these compounds provide a comprehensive view of the environmental risks posed by the pharmaceuticals used in treating neurodegenerative and mental health conditions. Pharmaceutical metabolites were included in this study. Typically, present at lower concentrations and with complex chemical structures, these compounds are more challenging to detect and analyze [57]. By monitoring both parent compounds and metabolites, this study aims to offer a more thorough understanding of the environmental impact of pharmaceuticals. Additionally, caffeine was included in this study because it is one of the most widely consumed substances worldwide [58]. Commonly found in beverages (e.g., coffee, tea, energy drinks, and soft drinks), in foods, and in pharmaceuticals (e.g., cold medicines, analgesics, diuretics, and stimulants) [59,60], caffeine is a frequent pollutant detected in aquatic environments [61,62,63], making its analysis essential for this research. Given this context, the primary aim of this study was to develop an analytical methodology using ultra-high-performance liquid chromatography (UHPLC) coupled with tandem mass spectrometry (MS/MS) for the detection and quantification of pharmaceuticals from both therapeutical classes. This includes fourteen pharmaceuticals for neurodegenerative diseases (amantadine, apomorphine, benserazide, carbidopa, donepezil, entacapone, galantamine, pramipexole, rasagiline, rivastigmine, ropinirole, rotigotine, safinamide, selegiline), eight psychiatric drugs (carbamazepine, citalopram, diazepam, fluoxetine, paroxetine, sertraline, trazodone, venlafaxine), and one stimulant (caffeine). Furthermore, our study also includes the analysis of seven metabolites: citalopram N-oxide, citalopram propionic acid, desmethylcitalopram, didemethylcitalopram, *O*-desmethylvenlafaxine, 10,11-epoxycarbamazepine, norfluoxetine. Thus, a total of 30 compounds were extracted using solid phase extraction (SPE) and analyzed using UHPLC-MS/MS in 25 samples, including surface waters (rivers, streams, and ocean) as well as wastewater (influent and effluent) from wastewater treatment plants (WWTPs).

## 2. Materials and Methods

Ultrapure water (resistivity of 18.2 MΩ.cm) was produced using a Simplicity 185 system (Millipore, Molsheim, France). Acetonitrile LC-MS grade was purchased by Carlo Erba (Valle del Reuil Cedex, France), methanol LC-MS grade was supplied by Honeywell (Seelze, Germany), propanol LC-MS was obtained from Sigma-Aldrich (Steinheim, Germany), and formic acid (PA-ACS) was acquired by Carlo Erba (Valle del Reuil Cedex, France). Ethylenediaminetetraacetic acid disodium salt 2-hydrate (Na_2_EDTA) was purchased from Panreac (Barcelona, Spain). Detailed information for the pharmaceuticals, isotopically labeled internal standards (ILIS), and solvents used to prepare the stock solution are presented in Table S1 (Supplementary Materials). All the stock solutions were stored at −20 °C. The working standard solutions containing all compounds were prepared in acetonitrile:ultrapure water (30:70, *v*/*v*).

Samples were vacuum filtered through a 0.45 µm nylon membrane filter and immediately extracted using SPE. The analytical method employed to extract different therapeutical classes in surface waters and wastewater, as described in the authors’ previous works [17,30], was applied to the pharmaceutical compounds targeted in the present study. In summary, Strata-X SPE cartridges (200 mg, 3 mL) were used for the extraction of pharmaceuticals from water samples. To each sample (50 mL of WWTP influent, 100 mL of WWTP effluent, or 250 mL of river water), a 0.1 M Na_2_EDTA solution was added to reach a final concentration of 0.1% (g solute/g solution). The pH of the samples was then adjusted to 2 using 37% hydrochloride acid. The pre-filtered samples were processed through SPE cartridges that were pre-conditioned with 5 mL of methanol and equilibrated with 5 mL of ultrapure water, followed by another 5 mL of ultrapure water adjusted to pH 2. The analytes were eluted using 10 mL of methanol, dried under a gentle stream of nitrogen, and reconstituted with 500 µL of a 30:70 (*v*/*v*) acetonitrile:ultrapure water mixture. Finally, 5 µL of the ILIS mixture was added to both the standards and the samples. The concentrations in the analysis were 100, 350, 200, 60, 100, and 1000 µg/L for carbamazepine-d10, caffeine 13C3, diazepam-d5, fluoxetine-d5, venlafaxine-d6, and ibuprofen-d3, respectively. Before the chromatographic analysis, sample extracts and standards were filtered through 0.22 µm PTFE syringe filters (Specanalitica, Carcavelos, Portugal).

The Nexera UHPLC-MS/MS-ESI (LCMS-8030) liquid chromatograph was sourced from Shimadzu Corporation (Kyoto, Japan). The equipment includes two solvent delivery pumps, a column oven, an auto-sampler, a degasser, and a triple-quadrupole mass spectrometer operating in electrospray ionization (ESI) mode. Control and data processing were performed using Lab Solutions software version 5.80 (Shimadzu Corporation, Kyoto, Japan). Argon was used as the collision-induced dissociation (CID) gas at a pressure of 230 kPa, while nitrogen was used as the nebulizing and drying gas. Auto-sampler was operated at 4 °C and the needle was rinsed before and after sample aspiration using acetonitrile:methanol:propanol (1:1:1, *v*/*v*/*v*).

In the LC-MS/MS analysis, rigorous criteria were applied for both quantification and identification: (i) detection in both the quantification and qualification MRM transitions; (ii) consistent retention time of the analyte in the samples, closely matching that of the reference standard under identical analytical conditions; and (iii) the ratio of quantifier to qualifier ions within ±20% of the ratio observed in the standard. Quantification was based on the linearity of the calibration curve and evaluated over the specified concentration range.

## 3. Results

### 3.1. Mass Spectrometry Optimization

To select the precursor ion for each analyte, chromatograms of individual standards at a concentration of 100 mg/L were recorded in full scan mode. All pharmaceuticals, except entacapone and citalopram propionic acid, showed a higher response in the positive electrospray ionization mode. Then, the direct injection of individual standard solutions at 100 mg/L was carried out, and the mass spectrometer was operated in multiple reaction monitoring (MRM) mode. Collision energies and product ions for both quantification and qualification were determined through MRM optimization. Two transitions were identified for each analyte, with the most intense transition used for quantification and the second most intense used for qualification. Detailed MS/MS parameters are provided in Table S2 (Supplementary Materials).

Optimization of source-dependent parameters was also conducted. A direct injection of a standard mixture solution, with each compound at a concentration of 10 mg/L, was carried out. The increments of the variables were set according to the equipment’s limitations. Specifically, the interface voltage (IV, kV) was varied from 0.5 to 5.0, nebulizing gas flow (NGF, L/min) from 0.5 to 3.0, drying gas flow (DGF, L/min) from 10 to 20, desolvation line temperature (DLT, °C) from 200 to 300, and heat block temperature (HBT, °C) from 200 to 500. The area of each compound was measured for each variable. The optimal parameters were determined as follows: 2.6 L/min for NGF, 15 L/min for DGF, 5.0 kV for IV, 300 °C for DLT, and 425 °C for HBT for the negative ionization mode and 2.6 L/min for NGF, 15 L/min for DGF, 5.0 kV for IV, 225 °C for DLT, and 425 °C for HBT for the positive ionization mode, correspondingly.

### 3.2. Chromatographic Conditions Optimization

The chromatographic columns and elution mode were tested and optimized for the set of the studied pharmaceuticals. Good resolution, peak shape, and reproducibility were the requirements used for the optimization of the chromatographic conditions. Thus, 0.1% formic acid in ultrapure water (A) and acetonitrile (B) were used in the program for the compounds with the positive ionization mode, and ultrapure water (A) and acetonitrile (B) were used in the program for the compounds with the negative ionization mode.

**Figure 1:**
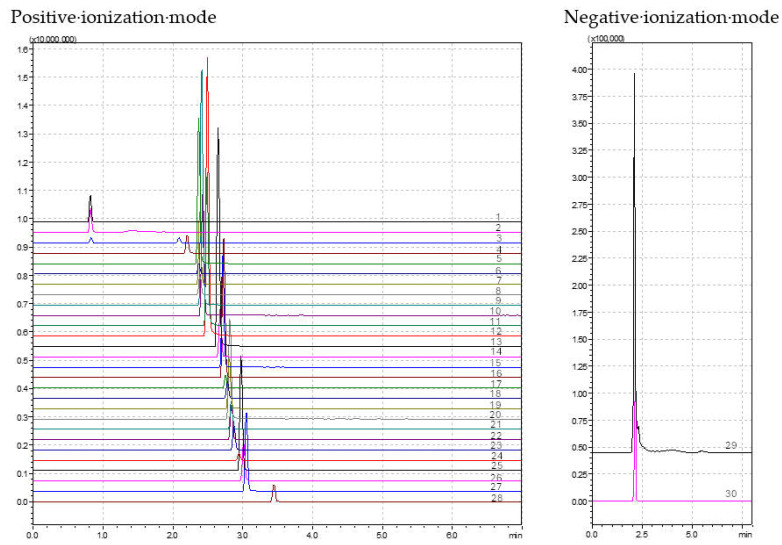
Chromatogram of the compounds analyzed in the positive and negative ionization modes (positive ionization mode: 1-benserazide, 2-pramipexole, 3-carbidopa, 4-galantamine, 5-rasagiline, 6-caffeine, 7-amantadine, 8-apormorphine, 9-ropinirole, 10-O-desmethylvenlafaxine, 11-rivastigmine, 12-selegiline, 13-venlafaxine, 14-trazodone, 15-safinamide, 16-donepezil, 17-didemethylcitalopram, 18-desmethylcitalopram, 19-citalopram, 20-rotigotine, 21-citalopram-n-oxide, 22-10,11-epoxycarbamazepine, 23-paroxetine, 24-norfluoxetine, 25-fluoxetine, 26-sertraline, 27-carbamazepine, and 28-diazepam; negative ionization mode: 29-entacapone and 30-citalopram propionic acid).

The isocratic or gradient mode of elution were evaluated; chromatographic columns, namely, Kinetex 1.7 µm, 150 × 2.1 mm (Phenomenex, Inc., Torrance, CA, USA) and Cortecs^TM^ UPLC^®^ C18+ 1.6 µm, 100 × 2.1 mm (Waters, Milford, MA, USA), and the proportion of the eluents in the mobile phase, were tested. The optimum results were found using the Cortecs^TM^ column in a gradient elution mode for both ionization modes. In the negative ionization mode, the optimized program starts with 5% (B), increases for 1 min to 100% (B), remains for 2.5 min, and returns to the initial conditions in 0.5 min. The total time was set for 8 min. In the positive ionization mode, the optimized program also starts with 5% (B), increasing over 3 min to 100% (B), remaining for 0.5 min, and returning to the initial conditions in 0.5 min. The total time was set for 7 min. The flow rate of each chromatographic program was 0.3 mL/min. The oven temperature for both ionization modes was set to 30 °C and an injection volume of 5 µL was used. A dwell time of 75 ms was used in the negative ionization mode and a dwell time of 10 ms was set for the compounds of the positive ionization mode. A chromatogram registered in each ionization mode of the selected pharmaceuticals is presented in Figure 1 and the retention time of each analyzed compound is presented in Table S3 (Supplementary Materials).

### 3.3. Validation of the Method Developed

The linearity of the method was performed by setting the calibration curves using linear regression analysis. The range of concentrations was set between 1.0 to 1000 μg/L (1.0, 5.0, 10.0, 25.0, 50.0, 75.0, 100, 250, 500, 750, and 1000 μg/L) and all compounds gave good fits (R ≥ 0.998, Table S3, Supplementary Materials). Quantification of the target analytes was performed by the internal standard approach. Regression equations for the quantifier and the qualifier ions are presented in Table S3 (Supplementary Materials).

To identify a compound using UHPLC-MS/MS, several analytical tools were used: retention time, the presence of the compound in both product ion transitions, and ion ratio. The ion ratio was calculated as the ratio between the area obtained from the quantifier ion and the area obtained from the qualifier ion. The results of the ion ratio are listed in Table S3 (Supplementary Materials).

The minimum detectable amount of analyte, with signal-to-noise ratios of 3 and 10, was used to determine the limits of detection (LOD) and quantification (LOQ). From the calibration curves prepared in solvent, LOD values ranged from 0.01 µg/L (for citalopram N-oxide, sertraline, fluoxetine, rotigotine, and rasagiline) to 20.2 µg/L (for pramipexole), while LOQ values ranged from 0.02 µg/L (for rasagiline, rotigotine, and citalopram N-oxide) to 67.3 µg/L (pramipexole) (Table S3, Supplementary Materials). Method detection limits (MDLs) and method quantification limits (MQLs) were determined in surface water and wastewater matrices (Figure S1, Supplementary Materials). Higher limits were found for wastewaters when compared with surface waters, with MDLs ranging from 0.0200 ng/L (sertraline and ropinirole) to 21.1 ng/L (norfluoxetine) in the surface water matrix, and from 0.200 ng/L (trazodone) to 55.5 ng/L (entacapone) in the wastewater matrix. In comparison, MQLs ranged between 0.0600 ng/L (sertraline and ropinirole) to 70.4 ng/L (norfluoxetine) in the surface water matrix, and between 0.650 ng/L (trazodone) to 185 ng/L (entacapone) in the wastewater matrix.

Intra- and inter-day analyses were also performed, and RSD was lower than 10%.

**Figure 2:**
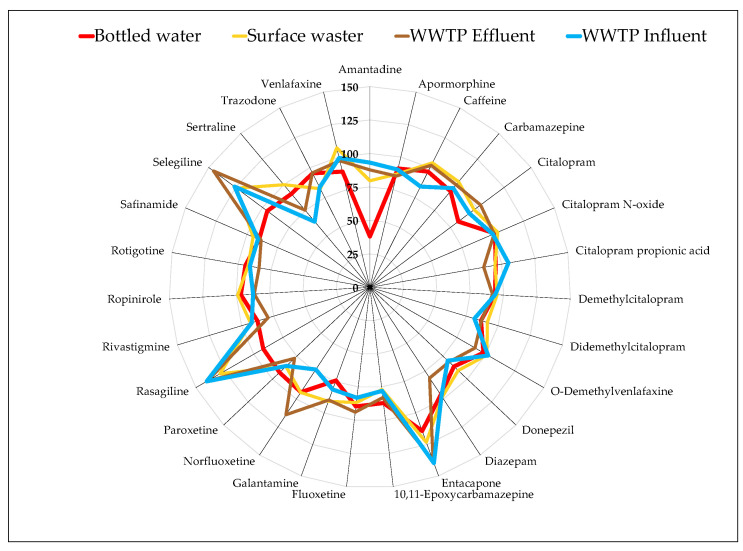
Recovery results for target compounds in different matrices: surface water and wastewaters effluent and influent.

Previous studies by the authors have demonstrated the effectiveness of using Strata-X cartridges for extracting several therapeutical classes of pharmaceuticals from surface water and wastewater matrices [3,17,18,19,30,61,64]. Therefore, the same cartridges and extraction procedure were used to extract the target compounds in the present study. The water matrices analyzed include bottled water, surface water (lake, ocean, and river waters), and wastewater (WWTP influents and WWTP effluents) samples. The average recovery rates observed for all types of samples were higher than 90% (90.4% for bottled water, 97.2% for surface water, 95.7% for WWTP effluents, and 93.8% for WWTP influents). Recoveries were generally consistent across all sample matrices, as illustrated in Figure 2. However, significant variations were observed for certain compounds. Specifically, amantadine exhibited lower recovery rates (38.0%) in bottled water compared to other matrices. Recovery rates lower than 50% were also reported for amantadine in other studies, with 45.7% in ultrapure water [42] and 24% in nanopure water [65]. The highest variability between the matrices was observed for norfluoxetine, with recoveries of 93.8% for bottled water, 94.0% for surface water, 114% for WWTP effluent, and 73.5% for WWTP influent, and for sertraline, with 91.1% for bottled water, 100% for surface water, 75.3% for WWTP effluent, and 64.1% for WWTP influent. Additionally, recoveries exceeding 120% were observed for entacapone, selegiline, and rasagiline in surface water and wastewater samples (WWTP effluent and influent), while recoveries in bottled water were approximately 100%. Three of the thirty compounds—benserazide, carbidopa, and pramipexole—did not exhibit satisfactory recoveries due to the inefficiency of the extraction procedure for these compounds.

The extraction methodology previously developed by the authors for various therapeutic classes, including psychiatric drugs and caffeine, demonstrated strong performance for most of the 14 pharmaceuticals used in treating AD and PD, with recovery values in this study comparable to those reported in other studies (Table S4, Supplementary Materials). The type of SPE cartridge—whether single or combination sorbents—and the use of online or offline SPE extraction in other studies are presented in Table S4 (Supplementary Materials) and further discussed in the Discussion section.

### 3.4. Concentration Found in Surface Waters and Wastewater Analysis

Twenty-nine compounds belonging to two therapeutic classes, along with the stimulant caffeine, were analyzed in twenty-five samples. Five samples were collected from each source: streams, rivers, ocean, WWTP effluent, and WWTP influent. One chromatogram of each type of analyzed sample is shown in Figures S2–S6 (Supplementary Materials). The detected compounds are identified in each chromatogram, and their concentrations are presented in Table 1, along with the relative standard deviations (RSDs) (blue color).

A total of twenty-one compounds were detected in at least one sample. Notably, didemethylcitalopram, norfluoxetine, apomorphine, benserazide, carbidopa, donepezil, galantamine, pramipexole, and safinamide were not detected in any of the analyzed samples. Fluoxetine was the only compound detected in all 25 samples, followed closely by caffeine, which was detected in 24 out of 25 samples. The highest concentration observed was for caffeine, with a value of 76,991 ng/L in sample E5. Significantly, 13 out of 15 psychiatric drugs (86.7%) were detected in at least one sample, highlighting their widespread presence. Additionally, 7 out of 14 compounds (50%) associated with AD and PD pharmaceuticals were also detected, underscoring the relevance of these compounds in the studied samples.

As can be observed in Table 1, the detected concentrations of caffeine ranged from 29.8 to 85.2 ng/L in ocean samples, 31.5 to 86.3 ng/L in streams, 31.6 to 656 ng/L in rivers, 140 to 76,991 ng/L in WWTP effluent, and 448 to 57,640 ng/L in WWTP influent samples. For psychiatric drugs, concentrations varied from <MDL to 15.7 ng/L in ocean samples, <MDL to 52.0 ng/L in streams, <MDL to 381 ng/L in rivers, <MDL to 5227 ng/L in WWTP effluent, and <MDL to 4027 ng/L in WWTP influent samples. Regarding pharmaceuticals administered for neurodegenerative diseases, only selegiline was detected in ocean samples (AO1, <MDL). A similar observation was made in rivers, with only amantadine detected in sample R1 (9.75 ng/L). In streams, concentrations ranged from <MDL to 37.1 ng/L, in WWTP effluent, from <MDL to 149 ng/L, and in WWTP influent samples, from <MDL to 206 ng/L.

Fluoxetine and caffeine were detected with a frequency of 100% in ocean samples; fluoxetine was detected with the same frequency in streams. In the river and in the WWTP effluent and influent samples, carbamazepine, *O*-desmethylvenlafaxine, fluoxetine, venlafaxine, and caffeine were all detected with a frequency of 100%.

Regarding the pharmaceuticals administered for AD and PD, amantadine and rivastigmine were detected in a higher number of samples. Amantadine was detected in 32% of the samples (8 out of 25), present in all WWTP effluent samples (49.0 to 149 ng/L), and in samples R1 (9.75 ng/L), I1 (55.4 ng/L), and I5 (206 ng/L). Amantadine is commonly used to manage symptoms of PD and similar conditions, such as muscle stiffness and tremors, and has also been employed as an antiviral agent against influenza A [34,49]. Its dual role in treating neurological disorders and influenza contributes to its frequent detection in environmental studies. In 2023, Chen Miao [44] reported that amantadine was among the most prevalent pharmaceuticals detected in river waters, with maximum concentrations significantly higher than the concentration observed in the present study, ranging from 0.10 to 1084 ng/L. The authors mentioned that high levels and high detection frequency could be related to the incomplete removal of amantadine during wastewater treatment processes. Rivastigmine was the second most detected pharmaceutical after amantadine, identified in 24% of the analyzed samples (6 out of 25). Rivastigmine was only detected in WWTP wastewaters samples with concentrations ranging from 2.79 to 37.0 ng/L in WWTP effluent and 15.0 ng/L in WWTP influent (sample I5). The higher detection rate of rivastigmine may be explained by its use in treating multiple diseases, particularly those involving cognitive impairment, such as AD and PD-related dementia. This broader therapeutic application results in higher consumption and, consequently, higher detection in environmental samples compared to other AD and PD pharmaceuticals. Relative to the other pharmaceuticals, entacapone was detected in one sample (R4) at a concentration of 37.1 ng/L, while rasagiline (L1), ropinirole (L2 and I4), rotigotine (I1), and selegiline (AO1 and E1) were detected at concentrations below MDL. The highest concentration was found for amantadine in sample E1 with 149 ng/L.

For psychiatric drugs, the highest concentration was found for fluoxetine (15.7 ng/L, AO2) in the ocean, carbamazepine (52.0 ng/L, L2) in streams, and *O*-desmethylvenlafaxine in rivers (381 ng/L, R1), WWTP effluent (5227 ng/L, E1), and WWTP influent (4027 ng/L, I1). For the stimulant caffeine, the highest concentrations were observed in wastewater samples, with 76,991 ng/L in E5 and 57,640 ng/L in I2 (Table 1). The high detection frequency and levels of caffeine can be attributed to its significant consumption, reflecting its widespread use and subsequent discharge into wastewater systems.

### 3.5. Concentration Detected in Micrograms per Liter Levels

Compounds with concentrations measured in µg/L found in both WWTP effluent and influent included carbamazepine (E1, E2, and I1), *O*-desmethylvenlafaxine (E1 to E5, I1, I2, I3, and I5), and caffeine (E1, E2, E5, I1, I2, and I3) (Table 1). The concentration levels increased with the complexity of the samples, being lower in surface waters and significantly higher in WWTP wastewater. This pattern is expected, as surface waters—being the receiving environment for wastewater discharges—undergo dilution, degradation, and adsorption to sediments [18].

### 3.6. Pharmaceutical Metabolites

Most monitoring studies typically concentrate on the parent compounds, with fewer studies focusing on their metabolites. In the present study, metabolites of carbamazepine (10,11-epoxycarbamazepine), citalopram (citalopram N-oxide, citalopram propionic acid, desmethylcitalopram, and didemethylcitalopram), fluoxetine (norfluoxetine), and venlafaxine (*O*-desmethylvenlafaxine) were analyzed.

As can be observed in Table 1, carbamazepine was detected in 19 of 25 samples and its metabolite 10,11- epoxycarbamazepine was detected in only two samples (R1 and E2). In R1 and E2, the concentration of carbamazepine was 4.21 to 14.7 times higher than the concentration of its metabolite. In contrast, the pattern for venlafaxine and its metabolite was different. Both compounds were detected in 16 out of 25 samples, but *O*-desmethylvenlafaxine generally had higher concentrations than venlafaxine. In streams, the metabolite concentration was 3.3 times higher than the concentration found for venlafaxine. In rivers, it ranged from 1.67 to 5.41 times higher, and in wastewater (effluent and influent), it was 4.27 to 9.38 times higher. The metabolite was found in lower concentrations than venlafaxine in only two samples, R3 and R4, where the difference of the obtained concentrations between them was minimal.

Relative to the fluoxetine and the metabolite norfluoxetine, fluoxetine was detected in all samples, whereas its metabolite was never detected. For citalopram, four metabolites were analyzed. Citalopram was detected in 8 out 25 samples with a concentration below MDL in R5, E2, E3, E4, E5, I1, and I2 and with concentration of 38.6 ng/L in E1. Relative to citalopram metabolites, citalopram N-oxide was detected in one sample (R2, <MDL), citalopram propionic acid was detected in four samples (R5, 35.1 ng/L; I3 < MDL; I4 < MDL; and I5, 46.7 ng/L), desmethylcitalopram was detected in two samples (R4, <MDL and E1, 38.0 ng/L), and didemethylcitalopram was never detected. Citalopram and its metabolites can be compared in two samples. In sample R5, citalopram was detected at levels below MDL, while its metabolite, citalopram propionic acid, was found at a concentration of 35.1 ng/L. In sample E1, similar concentrations of citalopram and desmethylcitalopram were observed.

Comprehensive environmental monitoring that accounts for both pharmaceuticals and their metabolites is essential for accurately assessing their impact on aquatic ecosystems. In 2024, Meyer et al. [66] revealed that metabolites represent a significant portion of the pharmaceutical load in wastewater, emphasizing the critical need to include these compounds in chemical risk assessments. Without this inclusion, the environmental and health hazards posed by pharmaceutical contamination are underestimated.

### 3.7. Risk Assessment

The potential environmental risk posed by the pharmaceuticals detected in surface waters in this study was assessed using the risk quotient (RQ) across three trophic levels in the aquatic ecosystem: algae, *daphnia magna*, and fish. The RQ is calculated as the ratio between the measured environmental concentration (MEC) of a compound and its predicted no-effect concentration (PNEC) (RQ = MEC/PNEC). Following EU guidelines, the ECOSAR predictive model (v1.11) was used. A worst-case scenario approach was adopted, using the highest concentration found in the ocean, streams, and rivers. Values of RQ < 0.1 represented the negligible risks of contaminants, 0.1 ≤ RQ < 1 represented the low risk, and 1 ≤ RQ < 10 and RQ ≥ 10 represented the moderate and high risk, respectively. Caffeine emerged as the only compound posing a risk to algae, with RQ values of 5.71 in the ocean, 5.76 in streams, and a higher RQ of 42.0 in rivers. The high consumption of caffeine and the levels found in the environment underscores the need for closer attention to caffeine’s environmental impact.

### 3.8. Pharmaceutical Literature Research: Key Findings and Information

Although extensive research has been conducted on psychiatric drugs [3,17,18,19,27,28,29,30,31], relatively few studies have focused on AD and PD pharmaceuticals. A literature review using the keywords ‘water samples’ and ‘environmental pollution’ identified 28 relevant studies [32,33,34,35,36,37,38,39,40,41,42,43,44,45,46,47,48,49,50,51,52,53,54,55,56,65,67,68]. While these studies analyze AD and PD pharmaceuticals in environmental contexts, they often group them with other pharmaceuticals, including psychiatric drugs (Table S4, Supplementary Materials). Among AD and PD pharmaceuticals, amantadine is the most studied, featured in 23 studies [32,33,34,35,36,37,38,39,40,41,42,43,44,45,46,47,48,49,50,56,65,67,68], likely due to its dual use for movement disorders and as an antiviral for influenza A [33,49]. In contrast, donepezil has been studied in four studies [51,52,53,54], rivastigmine [50,52,56] and ropinirole [42,46,50] in three studies each, galantamine in two studies [50,52], and benserazide [50] and entacapone [55] in one study each.

Most studies (23 of 28) have focused on analyzing a single AD or PD pharmaceutical from their list of pollutants [32,33,34,35,36,37,38,39,40,41,43,44,45,47,48,49,51,53,54,55,65,67,68]. Notably, V. Brieudes et al. [52] (2017) study donepezil, galantamine, and rivastigmine in river samples, while Wei L. et al. [56] (2020) studied amantadine and rivastigmine in surface water and WWTP influent and effluent samples. More recent studies, including B. Gonzalez-Gaya et al. [42] (2021) and N. Lopez-Herguedas et al. [46] (2023), assessed amantadine and ropinirole across various water matrices, ultrapure water [42], estuary [42], river [42], in WWTP effluent [42,46], and WWTP influent [46]. In 2023, Ng Kelsey et al. [50] investigated five AD and PD drugs—amantadine, benserazide, galantamine, rivastigmine, and ropinirole—in groundwater, river, and WWTP wastewaters (both influent and effluent). Significantly, no studies were found for apomorphine, carbidopa, selegiline, pramipexole, rasagiline, rotigotine, and safinamide. This highlights a critical gap in the current literature, which the present study seeks to address by providing essential data on the presence of these under-researched pharmaceuticals in various environmental water samples. The results from this study, detailed in the above subsections, along with the studies found in the literature, are discussed in the Discussion section.

## 4. Discussion

The findings from various studies, summarized in Table S4 (Supplementary Materials), detail the compounds, sample types, extraction methods, analysis techniques, recoveries, and concentrations for the compounds targeted in the present study. Most studies focused on the extraction and analysis of pharmaceuticals in WWTP influent [32,34,37,41,42,46,47,50,51,55,56,65], WWTP effluent [32,33,34,35,37,38,39,41,42,43,46,47,50,51,54,55,56,65,67,68], and river water [33,34,35,39,42,43,44,47,48,49,50,52,55,56,67,68] samples. Fewer studies targeted other surface waters, such as oceans [40,53], estuaries [42], and groundwater [42,43,50,55]. Drinking water was also analyzed, though in a limited number of studies [36,48].

Several studies have investigated the recovery rates of AD and PD pharmaceuticals from various water matrices, with amantadine being the most frequently analyzed. The first published work mentioned the analysis of amantadine in the WWTP effluent sample [32]. The SPE extraction was performed using the cartridge Oasis HLB (200 mg, 6 mL, Waters), and after a Sep-Pak plus NH_2_ (360 mg, Waters) cartridge for the clean-up step. Gonzalez-Gaya et al. [32] reported recovery rates of 95.2% in estuary water and 64.2% in WWTP effluent using a combination of Strata HR-X, ZT-WAX, and ZT-WCX sorbents, but found a recovery lower than 50% in ultrapure water. This aligns with our study, where amantadine showed a 38.0% recovery in bottled water (Figure 2). Similarly, Lahiruni M. Halwatura et al. [65] in 2023 found recoveries of 24% in nanopure water and 82% in wastewater influent. Zou et al. [43] reported amantadine recovery rates above 80% in groundwater, surface water, and WWTP effluent. Xu et al. [40] observed recoveries ranging from 46.8% to 60.1% in seawater using MCX sorbent (Waters). Gómez-Navarro et al. and Manjarrés-Lopez et al. [45,67] achieved recoveries between 70% and 120% in surface waters with mixed sorbents. For amantadine, recovery rates across the remaining studies were generally consistent, ranging from around 40% to 100% [33,35,37,41,46,50,56] in wastewater and from 60% to 112% [33,34,35,44,46,47,49,50,56] in surface and groundwater. For donepezil, rivastigmine, and galantamine, Brieudes et al. [52] achieved recoveries above 70% in natural waters using MCX sorbents (Waters), while Oliveira et al. [51] reported a 98% recovery for donepezil in hospital effluents. González-Gaya et al. [42] found ropinirole recoveries of 76.5% in Milli-Q water, 43.9% in WWTP effluent, and 82.3% in estuary water, with Lopez-Herguedas et al. [46] reporting a high recovery of 129% in wastewater influent. For entacapone, Ugolini V. et al. [55] obtained recoveries of 81% in ultrapure water and 69% in WWTP influent samples. For ropinirole, González-Gaya et al. [42] found a recovery of 76.5% in estuary water, 82.3% in ultrapure water, and 43% in WWTP effluent. Higher recoveries in wastewater were found in the study of Ng N. et al. [50] and Lopez-Herguedas et al. [46], with values of >60% [50] and 129% [46]. Ng N. et al. [50] also reported recoveries above 60% for groundwater.

As shown in Table S4 (Supplementary Materials), a single sorbent is typically used [32,33,34,35,37,40,44,47,48,52,53,55,56,68]. However, some studies report the use of a mixture of sorbents [38,42,45,46,61] or the use of the sorbents in tandem [65]. Most studies reported the use of SPE either offline [32,33,34,35,37,38,40,42,44,45,46,47,48,52,53,55,56,67,68] or online [43,50]. Additionally, a study published in 2015 described a method with the direct injection of hospital effluent for the analysis of donepezil [51]. In 2021, A.B. Martínez-Piernas et al. [54] demonstrated the versatility of QuEChERS methodology for extracting donepezil from treated wastewater samples. This study found recoveries between 85 and 112% for the 107 compounds analyzed.

The recoveries found in the studies presented in Table S4 (Supplementary Materials), particularly for psychiatric drugs and the stimulant caffeine, are similar to those observed in our study. Most studies reported recoveries higher than 70%, with the majority around 100%.

From the other studies and for all the compounds analyzed, it can be observed that recovery rates vary across different water matrices, with high recoveries also observed in more complex matrices such as wastewater. The following paragraphs discuss the pharmaceuticals detected and their levels as reported in the literature. A summary of the concentrations found for each compound across different water types is presented in Table 2.

Numerous studies have demonstrated the presence of several pharmaceuticals, particularly amantadine [32,33,34,35,36,37,38,39,40,41,42,43,44,45,46,47,48,49,50,56,65,67,68], carbamazepine [36,37,38,39,41,42,43,45,46,47,48,49,50,51,52,53,54,65,68], psychoactive drugs (trazodone [44,50,51,54], venlafaxine [37,38,41,43,44,45,50,51,52,53,54,65,67,68], sertraline [39,41,42,44,45,46,49,50,51,52,53,68], paroxetine [37,39,42,44,45,46,50,51,52,53,54,68], fluoxetine [41,44,49,50,51,52,53,54,68], diazepam [39,41,42,45,46,50,54,67,68], citalopram [38,41,44,45,49,50,52,53,54,65,67,68]), metabolites (desmethylcitalopram [50,51,52,67], *O*-desmethylvenlafaxine [41,44,45,47,49,50,51,52,67], 10,11-epoxycarbamazepine [41,45,50,51]), and the stimulant caffeine [39,41,42,45,46,47,49,50,51,65,67,68] in various aquatic environments, with amantadine and carbamazepine showing a 100% detection rate in several studies [32,44,47,49,52,54]. Comparative analysis reveals that the concentrations of these pharmaceuticals vary across different studies, ranging from ng/L to µg/L.

The presence of pharmaceuticals in the environment can be due to various pathways, including human excretion, improper disposal, agricultural runoff, wastewater treatment plants, industrial discharges, surface runoff, leaching from landfills, and biosolids application. Pharmaceuticals can also be discharged to the environment from animals through several mechanisms, including veterinary medicine, aquaculture, wildlife, manure application, migration and dispersal, and biological accumulation [69].

AD and PD pharmaceuticals are often analyzed with other therapeutic classes, including psychiatric drugs [36,37,38,39,41,42,43,44,45,46,47,48,49,50,51,52,53,54,65,67,68], whereas only a few studies focus specifically on anti-influenza drugs [32,33,34,35,40]. For instance, Ugolini, V et al. [55] analyzed antimicrobial chemicals, including entacapone, among 53 compounds. In our study, the highest concentrations detected were 206 ng/L in WWTP influent samples, 149 ng/L in WWTP effluent samples, 9.75 ng/L in river samples, all for amantadine, and 37.1 ng/L in stream samples, for entacapone. Notably, other studies have reported higher concentrations, with amantadine reaching µg/L levels in rivers [44,67]. 

The first study to report on the analysis of AD and PD pharmaceuticals in environmental water samples was conducted in 2010 by Ghosh G.C. et al. [32]. This study assessed the presence and behavior of two antiviral drugs—oseltamivir carboxylate and amantadine—in three sewage treatment plants during the 2008–2009 and 2009–2010 influenza seasons in Japan. Both pharmaceuticals were detected in all analyzed samples, with WWTP influent concentrations ranging from 140 to 460 ng/L for oseltamivir carboxylate and from 184 to 538 ng/L for amantadine. The study also found that primary treatment processes achieved minimal removal of these pharmaceuticals, reducing oseltamivir carboxylate by only 2–9% and amantadine by 7–17%, thus highlighting their persistence through initial wastewater treatment stages. Additionally, Xu Y et al. (2019) [40] reported being the first to monitor amantadine pollution in Laminaria japonica and seawater. 

Azuma T. et al. reported studies in 2013 [33], 2014 [34], and 2017 [35], in which amantadine was one of the anti-influenza pharmaceuticals analyzed. The authors mentioned the importance of introducing ozonation to reduce pollution loads in rivers. To their knowledge, the study was the first to evaluate the removal effects of natural sunlight, biodegradation, and sorption to river sediments on the tested anti-influenza drugs. Our study found lower amantadine concentrations compared to other research, highlighting the influence of factors such as seasonal variations, differences in wastewater treatment technologies, and regional variations in pharmaceutical usage (Table 2). For instance, Ghosh G.C. et al. (2010) reported the highest concentrations in WWTP influent, ranging from 230 to 580 ng/L [32]. Similarly, Azuma T. et al. (2014) found concentrations between 105.1 and 160.9 ng/L [34], and Vergeynst L. et al. (2015) reported levels from 44 to 326 ng/L [37]. In WWTP effluent samples, Zou J. et al. (2020) observed concentrations ranging from 400 to 630 ng/L [43], while Manjarrés-López et al. (2021) reported a significantly higher concentration of 19,800 ng/L [67]. Regarding river waters, the highest amantadine concentrations were reported by Manjarrés-López et al. (131 to 5704 ng/L [67]), Peng Y. et al. (72.6 to 883.6 ng/L [39]), and Chen M. et al. (0.10 to 1084 ng/L [44]), with levels reaching µg/L in the studies by Manjarrés-López et al. [67] and Chen M. et al. [44]. 

Hospital effluent and WWTP wastewater were analyzed in a study conducted by Oliveira et al. [51] in 2015, where donepezil was one of the target compounds. Results reveal it is undetected in WWTP wastewaters and ranges from n.d. to 50.0 ng/L in hospital effluents. Stream samples were only analyzed in our study, with entacapone (n.d.-37.1 ng/L), rasagiline (n.d.−<MDL), and ropinirole (n.d.−<MDL) detected in at least one sample.

Additionally, the presence of compounds like rivastigmine, which was detected in our study but often not in other studies, further emphasizes the variability in pharmaceutical pollution highlighting the importance of monitoring studies. Another example is the case of rivastigmine, which was undetected or found at lower levels in other studies; however, in our study, it was detected in WWTP wastewaters. In WWTP effluent, it was detected in one sample (one of five samples) with a concentration of 15.0 ng/L and in WWTP influent, the concentration ranged from 2.79 to 37.0 ng/L with 100% detection frequency. 

Two studies reported the detection of amantadine (1.99–2.45 ng/L [40]) and donepezil (9.7–180 ng/L [53]) in ocean samples. However, these compounds were not detected in the same type of sample of the present study, being the compound selegiline was the only compound detected (below the MDL) in the ocean. González-Gaya et al. [42] (2021) analyzed estuary samples, detecting amantadine only in the WWTP effluent at 21 ng/L. 

Groundwater studies [42,43,50,55] found n.d. [50] and 82 ng/L [43] for amantadine with benserazide [43], entacapone [55], galantamine [50], and ropinirole [42,50] undetected. For drinking water, two studies reported amantadine concentrations of 2.5–4.0 ng/L [48] and n.d.–21 ng/L [36].

Regarding psychiatric drugs, most studies have focused on analyzing carbamazepine, citalopram, diazepam, fluoxetine, paroxetine, sertraline, venlafaxine, and its metabolite *O*-desmethylvenlafaxine, with wastewaters and river water being the primary sample types studied (Table 2). The concentrations observed in our study closely align with those reported in other countries, reinforcing the consistency of these findings across different regions. Among these, the antidepressant fluoxetine stands out as a pollutant of significant environmental concern. Like many pharmaceuticals, fluoxetine typically enters the environment through human consumption and excretion [70]. Widely prescribed for conditions such as depression and anxiety, the compound fluoxetine’s prevalence in human populations contributes to its frequent detection in wastewater. Up to 30% of ingested fluoxetine is excreted unmetabolized [71], and its incomplete removal by sewage treatment plants [72] allows it to enter aquatic environments via wastewater effluent. Consequently, fluoxetine has been detected frequently in surface waters and wastewater samples [3,17,18,28,61,70,73,74,75,76]. From an environmental perspective, the continuous presence of fluoxetine poses risks for aquatic ecosystems, as pharmaceutical contaminants can impact aquatic organisms. Research by McDonald (2017) suggests that fluoxetine may impact wildlife through its pharmacological effects at concentrations lower than those required to induce general toxicity [77]. From a public health perspective, detecting fluoxetine in water sources raises concerns about unintentional human exposure. 

Low concentrations or non-detection for citalopram, citalopram N-oxide, citalopram propionic acid, desmethylcitalopram, diazepam, didemethylcitalopram, 10,11-epoxycarbamazepine, fluoxetine, paroxetine, sertraline, and trazodone were observed. Notably, no studies have specifically addressed the metabolites citalopram propionic acid and didemethylcitalopram.

Several factors influence the detection or non-detection of compounds and the levels detected in surface waters and wastewater samples. For example, (i) certain medications are taken more frequently during specific times of the year, (ii) the season in which samples were taken, (iii) meteorological conditions; (iv) the level at which compounds are detected; (v) elimination of the compound in WWTP treatments, (vi) deconjugation or reversion back to the parent form during biological treatments, (vii) adsorption or desorption of the compound to or from sludges, (viii) whether the effluent discharge into surface water is recent or not, and (ix) unknown discharges that can lead to uncontrolled levels in samples collected from river water.

Caffeine, due to its widespread consumption, is frequently detected in the environment, making it a crucial marker in monitoring studies. The levels observed in our study align with those reported in other research, with higher concentrations consistently found in WWTP effluent and influent samples, reaching the µg/L level.

The majority of these studies reported in Table 2 and Table S4 (Supplementary Materials) have been conducted in Spain [42,45,46,54,67], Japan [32,33,34,35,36], and China [39,40,44,56]. Additional studies were found in France [52], Belgium [37], Greece [38,41], Sweden [55], USA [51,65], India [43,68], Western Kenya [47,49], Ethiopia [48], and Fiji [53]. The study conducted by Ng. K. et al. [50] collected samples in the Danube River Basin in several countries (Croatia, Slovakia, Hungary, Czech Republic, Serbia, Bulgaria, Romania, Ukraine, Germany, Moldova, and Slovenia).

Monitoring studies is essential for assessing the environmental impact of pharmaceuticals, as they provide critical data on their presence and concentrations in various ecosystems.

## 5. Conclusions

This study developed and validated a robust SPE-UHPLC-MS/MS methodology to extract, and analyze 30 compounds, including pharmaceuticals for neurodegenerative diseases, psychiatric drugs and their metabolites, and caffeine in surface water and wastewater samples. 

Among the 30 targeted compounds, 21 were detected in at least one of the analyzed samples. Notably, all the studied psychiatric drugs (eight analytes) were identified, along with five of their metabolites, the stimulant caffeine, and seven pharmaceuticals used for the treatment of AD and PD.

Fluoxetine was the compound with 100% detection frequency. Caffeine was found in almost all samples (24 of 25), with the highest concentration reaching 76,991 ng/L, indicating its significant environmental presence due to widespread consumption. Concentrations of other pharmaceuticals varied, with psychiatric drugs ranging from <MDL to 5227 ng/L in wastewater and <MDL to 381 ng/L in surface water, while AD and PD pharmaceuticals ranged from <MDL to 206 ng/L in wastewater and <MDL to 37.1 ng/L in surface water.

The study underscores the need for the improved environmental monitoring of pharmaceutical residues to address the ongoing challenge posed by pharmaceutical contamination.

## Figures and Tables

**Table 1:** Concentrations of detected compounds in surface water (ocean, streams, and rivers) and wastewater (WWTP effluent and influent) samples.

**Compounds**	**Concentration (ng/L)**																								
	AO1	AO2	AO3	AO4	AO5	S1	S2	S3	S4	S5	R1	R2	R3	R4	R5	E1	E2	E3	E4	E5	I1	I2	I3	I4	I5
**Carbamazepine**	n.d.	n.d.	n.d.	n.d.	n.d.	n.d.	**52.0**	**20.5**	**48.5**	**26.7**	**108**	**11.4**	**17.6**	**19.0**	**150**	**1347**	**1005**	**599**	**318**	**446**	**1144**	**565**	**220**	**372**	**497**
RSD (%)							5.38	1.34	1.21	1.87	3.30	4.91	1.43	4.06	0.898	7.36	0.0341	1.55	1.32	2.98	0.618	2.91	2.34	6.12	9.41
**Citalopram N-oxide**	n.d.	n.d.	n.d.	n.d.	n.d.	n.d.	n.d.	n.d.	n.d.	n.d.	n.d.	**<MDL**	n.d.	n.d.	n.d.	n.d.	n.d.	n.d.	n.d.	n.d.	n.d.	n.d.	n.d.	n.d.	n.d.
RSD (%)																									
**Citalopram propionic acid**	n.d.	n.d.	n.d.	n.d.	n.d.	n.d.	n.d.	n.d.	n.d.	n.d.	n.d.	n.d.	n.d.	n.d.	**35.1**	n.d.	n.d.	n.d.	n.d.	n.d.	n.d.	n.d.	**<MDL**	**<MDL**	**46.7**
RSD (%)															3.58										13.2
**Citalopram**	n.d.	n.d.	n.d.	n.d.	n.d.	n.d.	n.d.	n.d.	n.d.	n.d.	n.d.	n.d.	n.d.	n.d.	**<MDL**	**38.6**	**<MDL**	**<MDL**	**<MDL**	**<MDL**	**<MDL**	**<MDL**	n.d.	n.d.	n.d.
RSD (%)																5.39									
**Desmethylcitalopram**	n.d.	n.d.	n.d.	n.d.	n.d.	n.d.	n.d.	n.d.	n.d.	n.d.	n.d.	n.d.	n.d.	**<MDL**	n.d.	**38.0**	n.d.	n.d.	n.d.	n.d.	n.d.	n.d.	n.d.	n.d.	n.d.
RSD (%)																0.729									
***O*-Desmethylvenlafaxine**	n.d.	n.d.	n.d.	n.d.	n.d.	n.d.	**35.5**	n.d.	n.d.	n.d.	**381**	**44.9**	**20.6**	**21.2**	**203**	**5227**	**4143**	**3583**	**1850**	**2938**	**4027**	**1949**	**821**	**1506**	**2609**
RSD (%)							1.27				11.3	11.1	1.13	2.78	1.50	3.04	6.94	5.41	8.66	12.4	0.174	3.05	6.50	18.2	2.32
**Diazepam**	n.d.	n.d.	n.d.	n.d.	n.d.	n.d.	n.d.	n.d.	n.d.	n.d.	n.d.	n.d.	n.d.	n.d.	n.d.	n.d.	n.d.	n.d.	**20.6**	n.d.	n.d.	n.d.	n.d.	n.d.	n.d.
RSD (%)																			1.05						
**Didemethylcitalopram**	n.d.	n.d.	n.d.	n.d.	n.d.	n.d.	n.d.	n.d.	n.d.	n.d.	n.d.	n.d.	n.d.	n.d.	n.d.	n.d.	n.d.	n.d.	n.d.	n.d.	n.d.	n.d.	n.d.	n.d.	n.d.
RSD (%)																									
**10,11-Epoxycarbamazepine**	n.d.	n.d.	n.d.	n.d.	n.d.	n.d.	n.d.	n.d.	n.d.	n.d.	**25.7**	n.d.	n.d.	n.d.	n.d.	n.d.	**68.4**	n.d.	n.d.	n.d.	n.d.	n.d.	n.d.	n.d.	n.d.
RSD (%)											10.2						1.23								
**Fluoxetine**	**5.62**	**15.7**	**5.55**	**5.35**	**5.67**	**5.64**	**5.60**	**5.63**	**6.02**	**5.77**	**5.82**	**5.54**	**5.79**	**6.16**	**8.06**	**17.1**	**14.3**	**60.3**	**20.2**	**14.2**	**58.5**	**99.0**	**28.9**	**29.6**	**29.7**
RSD (%)	0.203	9.65	2.76	6.12	0.604	0.723	1.72	1.16	3.09	4.86	3.42	9.40	3.89	5.00	1.86	4.22	10.1	12.1	3.52	10.5	12.2	1.91	0.206	1.58	2.12
**Norfluoxetine**	n.d.	n.d.	n.d.	n.d.	n.d.	n.d.	n.d.	n.d.	n.d.	n.d.	n.d.	n.d.	n.d.	n.d.	n.d.	n.d.	n.d.	n.d.	n.d.	n.d.	n.d.	n.d.	n.d.	n.d.	n.d.
RSD (%)																									
**Paroxetine**	n.d.	**<MDL**	n.d.	n.d.	n.d.	n.d.	n.d.	n.d.	n.d.	n.d.	n.d.	n.d.	n.d.	n.d.	n.d.	n.d.	n.d.	n.d.	n.d.	n.d.	n.d.	n.d.	n.d.	n.d.	n.d.
RSD (%)																									
**Sertraline**	n.d.	**4.28**	**<MDL**	n.d.	**6.41**	n.d.	n.d.	n.d.	n.d.	n.d.	n.d.	n.d.	n.d.	n.d.	n.d.	n.d.	n.d.	**27.4**	n.d.	n.d.	n.d.	n.d.	n.d.	n.d.	n.d.
RSD (%)		11.67			3.73													18.6							
**Trazodone**	n.d.	n.d.	n.d.	n.d.	n.d.	n.d.	n.d.	**<MDL**	**<MDL**	n.d.	n.d.	n.d.	n.d.	n.d.	n.d.	**<MDL**	**<MDL**	**92.9**	**<MDL**	**<MDL**	**<MDL**	**<MDL**	**<MDL**	**<MDL**	**<MDL**
RSD (%)																		9.0							
**Venlafaxine**	n.d.	n.d.	n.d.	n.d.	n.d.	n.d.	**10.9**	n.d.	n.d.	n.d.	**70.4**	**26.9**	**22.1**	**24.0**	**65.1**	**862**	**665**	**513**	**393**	**506**	**773**	**460**	**87.5**	**330**	**481**
RSD (%)							4.90				4.18	2.48	9.19	0.949	1.58	10.4	2.66	1.76	3.56	7.78	0.501	1.84	5.39	7.68	4.17
**Amantadine**	n.d.	n.d.	n.d.	n.d.	n.d.	n.d.	n.d.	n.d.	n.d.	n.d.	**9.75**	n.d.	n.d.	n.d.	n.d.	**149**	**95.9**	**97.5**	**109**	**49.0**	**55.4**	n.d.	n.d.	n.d.	**206**
RSD (%)											8.82					11.7	2.49	4.97	1.68	17.2	15.1				16.9
**Apormorphine**	n.d.	n.d.	n.d.	n.d.	n.d.	n.d.	n.d.	n.d.	n.d.	n.d.	n.d.	n.d.	n.d.	n.d.	n.d.	n.d.	n.d.	n.d.	n.d.	n.d.	n.d.	n.d.	n.d.	n.d.	n.d.
RSD (%)																									
**Benserazide**	n.d.	n.d.	n.d.	n.d.	n.d.	n.d.	n.d.	n.d.	n.d.	n.d.	n.d.	n.d.	n.d.	n.d.	n.d.	n.d.	n.d.	n.d.	n.d.	n.d.	n.d.	n.d.	n.d.	n.d.	n.d.
RSD (%)																									
**Carbidopa**	n.d.	n.d.	n.d.	n.d.	n.d.	n.d.	n.d.	n.d.	n.d.	n.d.	n.d.	n.d.	n.d.	n.d.	n.d.	n.d.	n.d.	n.d.	n.d.	n.d.	n.d.	n.d.	n.d.	n.d.	n.d.
RSD (%)																									
**Donepezil**	n.d.	n.d.	n.d.	n.d.	n.d.	n.d.	n.d.	n.d.	n.d.	n.d.	n.d.	n.d.	n.d.	n.d.	n.d.	n.d.	n.d.	n.d.	n.d.	n.d.	n.d.	n.d.	n.d.	n.d.	n.d.
RSD (%)																									
**Entacapone**	n.d.	n.d.	n.d.	n.d.	n.d.	n.d.	n.d.	n.d.	**37.1**	n.d.	n.d.	n.d.	n.d.	n.d.	n.d.	n.d.	n.d.	n.d.	n.d.	n.d.	n.d.	n.d.	n.d.	n.d.	n.d.
RSD (%)									0.944																
**Galantamine**	n.d.	n.d.	n.d.	n.d.	n.d.	n.d.	n.d.	n.d.	n.d.	n.d.	n.d.	n.d.	n.d.	n.d.	n.d.	n.d.	n.d.	n.d.	n.d.	n.d.	n.d.	n.d.	n.d.	n.d.	n.d.
RSD (%)																									
**Pramipexole**	n.d.	n.d.	n.d.	n.d.	n.d.	n.d.	n.d.	n.d.	n.d.	n.d.	n.d.	n.d.	n.d.	n.d.	n.d.	n.d.	n.d.	n.d.	n.d.	n.d.	n.d.	n.d.	n.d.	n.d.	n.d.
RSD (%)																									
**Rasagiline**	n.d.	n.d.	n.d.	n.d.	n.d.	**<MDL**	n.d.	n.d.	n.d.	n.d.	n.d.	n.d.	n.d.	n.d.	n.d.	n.d.	n.d.	n.d.	n.d.	n.d.	n.d.	n.d.	n.d.	n.d.	n.d.
RSD (%)																									
**Rivastigmine**	n.d.	n.d.	n.d.	n.d.	n.d.	n.d.	n.d.	n.d.	n.d.	n.d.	n.d.	n.d.	n.d.	n.d.	n.d.	**37.0**	**22.8**	**9.68**	**2.79**	**14.6**	n.d.	n.d.	n.d.	n.d.	**15.0**
RSD (%)																7.14	6.67	17.5	23.3	2.44					1.79
**Ropinirole**	n.d.	n.d.	n.d.	n.d.	n.d.	n.d.	**<MDL**	n.d.	n.d.	n.d.	n.d.	n.d.	n.d.	n.d.	n.d.	n.d.	n.d.	n.d.	n.d.	n.d.	n.d.	n.d.	n.d.	**<MDL**	n.d.
RSD (%)																									
**Rotigotine**	n.d.	n.d.	n.d.	n.d.	n.d.	n.d.	n.d.	n.d.	n.d.	n.d.	n.d.	n.d.	n.d.	n.d.	n.d.	n.d.	n.d.	n.d.	n.d.	n.d.	**<MDL**	n.d.	n.d.	n.d.	n.d.
RSD (%)																									
**Safinamide**	n.d.	n.d.	n.d.	n.d.	n.d.	n.d.	n.d.	n.d.	n.d.	n.d.	n.d.	n.d.	n.d.	n.d.	n.d.	n.d.	n.d.	n.d.	n.d.	n.d.	n.d.	n.d.	n.d.	n.d.	n.d.
RSD (%)																									
**Selegiline**	**<MDL**	n.d.	n.d.	n.d.	n.d.	n.d.	n.d.	n.d.	n.d.	n.d.	n.d.	n.d.	n.d.	n.d.	n.d.	**<MDL**	n.d.	n.d.	n.d.	n.d.	n.d.	n.d.	n.d.	n.d.	n.d.
RSD (%)																									
**Caffeine**	**72.3**	**44.8**	**29.8**	**85.7**	**34.3**	n.d.	**33.2**	**31.5**	**86.3**	**33.2**	**630**	**121**	**31.6**	**122**	**213**	**12,018**	**9164**	**318**	**130**	**76,991**	**9802**	**57,640**	**38,001**	**448**	**536**
RSD (%)	8.38	6.48	15.1	6.44	5.83		0.479	4.64	1.72	16.4	14.5	6.23	13.2	1.91	6.36	6.21	0.0249	2.12	1.49	4.90	3.37	7.91	7.70	17.0	0.638

<MDL, below method detection limit; n.d., not detected.

**Table 2:** Concentrations of detected pharmaceuticals in various types of water samples, including hospital effluent, WWTP influent, WWTP effluent, river, ocean, stream, estuary, groundwater, and drinking water.

Compound	Concentration (ng/L) (Note: INA, Information Not Available)							
	Hospital Effluent	WWTP Influent	WWTP Effluent	River	Ocean	Estuary	Groundwater	Drinking Water
Amantadine		**n.d.−580** [32,34,37,41,46,47,50,56]	**n.d.−19,800** [32,33,34,35,37,38,41,42,43,46,47,50,56,65,67]	**nd.−1084** [33,34,35,39,42,43,44,47,48,49,50,56,67,68]	**1.99–2.45** [40]	**n.d.** [42]	**n.d.−82** [43,50]	**n.d.−21** [36,48]
Benserazide		**n.d.** [50]	**n.d.** [50]	**1.80–30.2** [50]			**n.d.** [50]	
Donepesil	**n.d.−50** [51]	**n.d.** [51]	**n.d.** [51,54]	**n.d.** [52]	**9.7–180** [53]			
Entacapone		**n.d.** [55]	**n.d.** [55]	**n.d.** [55]			**n.d.** [55]	
Galantamine		**n.d.** [50]	**n.d.** [50]	**n.d.** [50,52]			**n.d.** [50]	
Rivastigmine		**INA−n.d.** [50,56]	**INA−n.d.** [50,56]	**INA−<LOQ-0.79** [50,52,56]			**n.d.** [50]	
Ropinirole		**n.d.−6** [42,46,50]	**n.d−29** [42,46,50]	**n.d.** [42,50]			**n.d.** [42,50]	
Caffeine	**45,740–325,000** [51]	**n.d.−830,659** [41,46,47,50,51,65]	**n.d.−170,889** [41,42,46,47,50,51,67]	**n.d.−666** [39,42,47,49,50,67,68]		**28** [42]	**0.39–3.80** [50]	
Carbamazepine	**20–620** [51]	**n.d.−1610** [37,41,46,47,50,51,65]	**n.d.−1700** [37,38,41,42,43,46,47,50,51,54]	**n.d.−210** [39,42,43,47,48,49,50,52,68]	**1.2–8.9** [53]	**n.d.** [42]	**0.10–170** [43,50]	**n.d.−10** [36,48]
Citalopram		**n.d.−700** [41,50,65]	**n.d.−235** [38,41,50,54,67]	**n.d.−180** [44,49,50,52,67,68]	**7.0–85** [53]		**n.d.** [50]	
Ctalopram N-oxide		**n.d.−13.0** [50]	**n.d.−5.10** [50]	**n.d.** [50]			**n.d.** [50]	
Desmethylcitalopram	**30–210** [51]	**n.d.−40** [50,51]	**n.d.−121** [50,51,67]	**n.d.−93.1** [50,52,67]			**n.d.** [50]	
*O*-Desmethylvenlafaxine	**620–2500** [51]	**n.d.−1480** [41,47,50,51]	**n.d.−2758** [41,47,50,51,67]	**n.d.−2077** [44,47,49,50,52,67]			**n.d.** [50]	
Diazepam		**n.d.−20** [41,46,50]	**n.d.−11.6** [41,42,46,50,54,67]	**n.d.−100** [39,42,50,67,68]		**n.d.** [42]	**n.d.** [50]	
10,11-Epoxycarbamazepine	**40–100** [51]	**1.40–70** [41,50,51]	2–60 [41,50,51]	**n.d.** [50]			**<LOQ−1.40** [50]	
Fluoxetine	**20–230** [51]	**6.10–100** [41,50,51]	**n.d.**−130 [41,50,51]	**n.d.−0.84** [44,49,50,52,54,68]	**n.d.** [53]		**n.d.** [50]	
Norfluoxetine	**n.d.−30** [51]	**n.d.** [50,51]	**n.d.** [50,51]	**n.d.** [50,52]			**n.d.** [50]	
Paroxetine	**n.d.−380** [51]	**n.d.** [37,46,50,51]	**n.d.−<MQL** [37,42,46,50,51,54]	**n.d.−0.04** [39,42,44,50,52,68]	**11–370** [53]		**n.d.** [46]	
Sertraline	**20–150** [51]	**0.53–140** [41,46,50,51]	**n.d.−80** [41,42,46,50,51]	**n.d.−1.12** [39,41,42,44,49,50,52,68]	**12–140** [53]	**n.d.** [42]		
Trazodone	**30–1160** [50]	**n.d.−80** [50,51]	**n.d.−58** [50,51,54]	**n.d.−0.08** [44,50]				
Venlafaxine	**170–660** [51]	**n.d.−1450** [37,41,50,51,65]	**0.21–1000** [37,38,41,43,50,51,54,67]	**n.d.−20** [43,44,50,52,68]	**5.7–758** [53,67]		**0.21–1000** [37,38,41,43,50,51,54,67]	

## Data Availability

The original contributions presented in the study are included in the article/Supplementary Material, further inquiries can be directed to the corresponding authors.

## Supplementary Materials

The following supporting information can be downloaded at: https://www.mdpi.com/article/10.3390/jox14040096/s1. Figure S1: Method detection limits for surface water and wastewater matrices for each studied compound. Figure S2: Overlay chromatogram of the detected compounds in ocean water sample AO2. Figure S3: Overlay chromatogram of the detected compounds in stream water sample S3. Figure S4: Overlay chromatogram of the detected compounds in river water sample R1, Figure S5: Overlay chromatogram of the detected compounds in WWTP effluent wastewater sample E1. Figure S6: Overlay chromatogram of the detected compounds in WWTP influent wastewater sample I2. Table S1: Pharmaceuticals, isotopically labeled internal standards (ILIS), chemical abstracts service (CAS), formula, molecular weight, supplier company, and solvent used for stock solution preparation. Table S2: Therapeutic class, pharmaceuticals, ionization mode, precursor and product ions, mass spectrometry conditions, ion ratio, and isotopically labeled internal standards (ILIS) for each pharmaceutical in the study. Table S3: Retention time, regression (equation and determination coefficient (R)), detection and quantitation limits (LOD and LOQ) for each transition, and ion ratio for each pharmaceutical. Table S4: Pharmaceuticals used in Neurodegenerative Treatments: bibliography search in the extraction and analysis.
