# Supplementary material for: Tracing Pharmaceuticals in Water Systems: Focus on Neurodegenerative and Psychiatric Treatments
Source: J Xenobiot. 2024 Nov 21;14(4):1807–25. doi: 10.3390/jox14040096 (PMC11586952; doi:10.3390/jox14040096)
Supplement: Supplementary file 1 [file jox-14-00096-s001.zip › jox-3284710-supplementary.pdf]

Supplementary Materials

Tracing Pharmaceuticals in Water Systems: Focus on Neurodegenerative and Psychiatric Treatments

Paula Paíga and Cristina Delerue-Matos

Number of pages: 40

Number of figures: 6

Number of tables: 4

List of tables

Table		Page
Table S1	Pharmaceuticals, isotopically labeled internal standards (ILIS), chemical abstracts service (CAS), formula, molecular weight, supplier company, and solvent used for stock solution preparation.	3
Table S2	Therapeutic class, pharmaceuticals, ionization mode, precursor and product ions, mass spectrometry conditions, ion ratio, and isotopically labeled internal standards (ILIS) for each pharmaceutical in the study.	4
Table S3	Retention time, regression (equation and determination coefficient (R)), detection and quantitation limits (LOD and LOQ) for each transition, and ion ratio for each pharmaceutical.	5-6
Table S4	Pharmaceuticals used in Neurodegenerative Treatments- bibliography search in the extraction and analysis.	7-34

List of Figures

Figure		Page
Figure S1	Method detection limits for surface water and wastewater matrices for each studied compound.	35
Figure S2	Overlay chromatogram of the detected compounds in ocean water sample AO2.	36
Figure S3	Overlay chromatogram of the detected compounds in stream water sample S3.	37
Figure S4	Overlay chromatogram of the detected compounds in river water sample R1.	38
Figure S5	Overlay chromatogram of the detected compounds in WWTP effluent wastewater sample E1.	39
Figure S6	Overlay chromatogram of the detected compounds in WWTP influent wastewater sample I2.	40

Table S1. Pharmaceuticals, isotopically labeled internal standards (ILIS), chemical abstracts service (CAS), formula, molecular weight, supplier company, and solvent used for stock solution preparation.

**Pharmaceuticals, metabolites, transformation products, and Isotopically Labeled Internal Standards (ILIS)** Note 1	**CAS** Note 2,3	**Formula (Molecular Weight)** Note 3	**Supplier Company**	**The solvent used for the preparation of each stock solution**
Amantadine hydrochloride	665-66-7	$C_{10}H_{17}N$ HCl (MW= 187.71 g/mol)	Sigma-Aldrich (Madrid, Spain)	Water
Apomorphine hydrochloride	41372-20-7	$C_{17}H_{17}NO_2$ HCl 1/2H_2O (MW= 312.79 g/mol)	Sigma- Aldrich (Madrid, Spain)	Water
Benserazide hydrochloride	14919-77-8	$C_{10}H_{15}N_3O_5$ HCl (MW= 293.70 g/mol)	Sigma-Aldrich (Madrid, Spain)	Water
Caffeine	58-08-2	$C_8H_{10}N_4O_2$ (MW= 194.19 g/mol)	Sigma-Aldrich (Madrid, Spain)	Methanol
Carbamazepine	298-46-4	$C_{15}H_{12}N_2O$ (MW= 236.274 g/mol)	Sigma-Aldrich (Madrid, Spain)	Methanol
Carbidopa	38821-49-7	$C_{10}H_{14}N_2O_4$ H_2O (MW= 244.24 g/mol)	Sigma-Aldrich (Madrid, Spain)	Methanol
Citalopram	59729-33-8	$C_{20}H_{21}FN_2O$ (MW= 324.399 g/mol)	Sigma-Aldrich (Madrid, Spain)	Methanol
Citalopram N-oxide hydrochloride	62498-71-9	$C_{20}H_{22}ClFN_2O_2$ (MW= 376.856 g/mol)	H. Lundbeck (Copenhagen, Denmark)	Methanol
Citalopram propionic acid*	Not Available	$C_{18}H_{14}FNO_3$ (MW= 311.312 g/mol)	H. Lundbeck (Copenhagen, Denmark)	Methanol
Entacapone	130929-57-6	$C_{14}H_{15}N_3O_5$ (MW= 305.29 g/mol)	Sigma-Aldrich (Madrid, Spain)	Ethanol
Demethylcitalopram hydrochloride	97743-99-2	$C_{19}H_{20}ClFN_2O$ (MW= 346.83 g/mol)	H. Lundbeck (Copenhagen, Denmark)	Methanol
O-Desmethylvenlafaxine	93413-62-8	$C_{16}H_{25}NO_2$ (MW= 263.381 g/mol)	Sigma-Aldrich (Madrid, Spain)	Purchased as a methanolic solution
R(-)-Deprenyl hydrochloride (Selegiline hydrochloride)	14611-52-0	$C_{13}H_{17}N$ HCl (MW= 223.74 g/mol)	Sigma-Aldrich (Madrid, Spain)	Water
Diazepam	439-14-5	$C_{16}H_{13}ClN_2O$ (MW= 284.743 g/mol)	Lipomed AG (Arlesheim, Switzerland)	Methanol
Didemethylcitalopram hydrochloride	1189694-81-2	$C_{18}H_{18}ClFN_2O$ (MW= 332.803 g/mol)	H. Lundbeck (Copenhagen, Denmark)	Methanol
Donepezil hydrochloride	120011-70-3	$C_{24}H_{29}NO_3$ HCl (MW= 415.95 g/mol)	Sigma-Aldrich (Madrid, Spain)	Water
10,11-Epoxycarbamazepine	36507-30-9	$C_{15}H_{12}N_2O_2$ (MW= 252.273 g/mol)	Sigma-Aldrich (Madrid, Spain)	Methanol
Fluoxetine hydrochloride	56296-78-7	$C_{17}H_{19}ClF_3NO$ (MW= 345.79 g/mol)	Sigma-Aldrich (Madrid, Spain)	Methanol
Galantamine hydrochloride	1953-04-4	C17H21NO3 HBr (MW= 368.27 g/mol)	Sigma-Aldrich (Madrid, Spain)	Water
Norfluoxetine hydrochloride	57226-68-3	$C_{16}H_{17}ClF_3NO$ (MW= 331.763 g/mol)	Sigma-Aldrich (Madrid, Spain)	Methanol
Paroxetine hydrochloride	78246-49-8	$C_{19}H_{21}ClFNO_3$ (MW= 365.829 g/mol)	Sigma-Aldrich (Madrid, Spain)	Methanol
Pramipexole dihydrochloride monohydrate	191217-81-9	$C_{10}H_{17}N_3S$ 2HCl H_2O (MW=302.26 g/mol)	Sigma-Aldrich (Madrid, Spain)	Water
Safinamide mesylate salt	202825-46-5	$C_{17}H_{19}FN_2O_2$ xCH_4O_3S (MW=302.34 g/mol)	Sigma-Aldrich (Madrid, Spain)	Water
Sertraline hydrochloride	79559-97-0	$C_{17}H_{18}Cl_3N$ (MW= 342.688 g/mol)	Sigma-Aldrich (Madrid, Spain)	Methanol
Rasagiline mesylate	161735-79-1	$C_{12}H_{13}N$ CH_4O_3S (MW= 267.34 g/mol)	Sigma-Aldrich (Madrid, Spain)	Water
Rivastigmine hydrogen tartrate	129101-54-8	$C_{14}H_{22}N_2O_2$ $C_4H_6O_6$ (MW= 400.42 g/mol)	Sigma-Aldrich (Madrid, Spain)	Water
Ropinirole hydrochloride	91374-20-8	$C_{16}H_{24}N_2O$ HCl (MW= 296.84 g/mol)	Sigma-Aldrich (Madrid, Spain)	Water
Rotigotine hydrochloride	125572-93-2	$C_{19}H_{25}NOS$·HCl (MW= 351.93 g/mol)	Sigma-Aldrich (Madrid, Spain)	Ethanol
Trazodone hydrochloride	19666-36-5	$C_{19}H_{23}Cl_2N_5O$ (MW= 408.327 g/mol)	Sigma-Aldrich (Madrid, Spain)	Methanol
Venlafaxine hydrochloride	99300-78-4	$C_{17}H_{28}ClNO_2$ (MW= 313.866 g/mol)	Sigma-Aldrich (Madrid, Spain)	Methanol
**ILIS**, Carbamazepine-d10	132183-78-9	$C_{15}H_2D_{10}N_2O$ (MW= 246.33 g/mol)	Cerilliant-Certified Reference Materials (Texas, USA)	Purchased as a methanolic solution
**ILIS**, Diazepam-d5	65854-76-4	$C_{16}H_8D_5ClN_2O$ (MW= 289.77 g/mol)	Lipomed AG (Arlesheim, Switzerland)	Purchased as a methanolic solution
**ILIS**, Fluoxetine-d5 hydrochloride	1173020-43-3	$C_{17}H_{14}D_5ClF_3NO$ (MW= 350.821 g/mol)	Sigma-Aldrich (Madrid, Spain)	Methanol
**ILIS**, Venlafaxine-d6	1062606-12-5	$C_{17}H_{22}ClD_6NO_2$ (MW= 319.90 g/mol)	Cerilliant-Certified Reference Materials (Texas, USA)	Purchased as a methanolic solution
**ILIS**, Caffeine $^{13}C_3$	78072-66-9	$^{13}C_3C_5H_{10}N_4O_2$ (MW= 197.17g/mol)	Sigma-Aldrich (Madrid, Spain)	Methanol
**ILIS**, Ibuprofen -d3	121662-14-4	$C_{13}H_{18}D_3O_2$ (MW= 209.30 g/mol)	Sigma-Aldrich (Madrid, Spain)	Acetonitrile

Note 1: Pharmaceuticals are organized in the table by alphabetic order. Note 2: Chemical Abstracts Service, CAS (a unique numerical identifier assigned by the Chemical Abstracts Service to every chemical substance described in the open scientific literature). Note 3: CAS, formula, and molecular weight for all the compounds were obtained in the website of PubChem (PubChem is an open chemistry database at the National Institutes of Health (NIH) [Available at: https://pubchem.ncbi.nlm.nih.gov/], with exception for citalopram propionic acid [Available at: http://www.hmdb.ca/metabolites/HMDB60463].

Table S2. Therapeutic class, pharmaceuticals, ionization mode, precursor and product ions, mass spectrometry conditions, ion ratio, and isotopically labeled internal standards (ILIS) for each pharmaceutical in the study.

Diseases	*Pharmaceuticals, metabolites, transformation products, and isotopically labeled Internal Standards (ILIS)* Note 1	ESI	Precursor (m/z)	Quantitation product				Qualifier Product				Dwell Time (msec)	ILIS
				m/z	*Q1 Pre Bias (V)*	*CE*	*Q3 Pre Bias (V)*	*m/z*	*Q1 Pre Bias (V)*	*CE*	*Q3 Pre Bias (V)*		
Used to treat Parkinson's disease	**Amantadine**	**ESI+**	151.95	135.05	-20.0	-20.0	-20.0	79.0	-13.0	-34.0	-16.0	10	**3**
Used to treat Parkinson's disease	**Apomorphine**	**ESI+**	268.10	191.05	-20.0	-30.0	-20.0	237.00	-20.0	-20.0	-20.0	10	**3**
Used to treat Parkinson's disease	**Benserazide**	**ESI+**	257.90	120.00	-20.0	-10.0	-20.0	103.00	-20.0	-20.0	-20.0	10	**3**
Stimulant	**Caffeine**	**ESI+**	194.95	138.00	-22.0	-21.0	-13.0	42.00	-23.0	-41.0	-16.0	10	**5**
Pshychiatric drugs	**Carbamazepine**	**ESI+**	236.95	193.95	-18.0	-21.0	-23.0	193.00	-26.0	-36.0	-13.0	10	**1**
Used to treat Parkinson's disease	**Carbidopa**	**ESI+**	227.00	181.20	-19.0	-14.0	-12.0	71.00	-18.0	-24.0	-13.0	10	**3**
Psychiatric drugs	**Citalopram**	**ESI+**	325.05	108.95	-16.0	-29.0	-23.0	261.95	-16.0	-22.0	-18.0	10	**3**
Psychiatric drugs-metabolite	**Citalopram N-oxide**	**ESI+**	341.05	108.95	-17.0	-26.0	-10.0	261.95	-28.0	-19.0	-18.0	10	**3**
Psychiatric drugs-metabolite	**Citalopram propionic acid**	**ESI-**	310.05	266.10	15.0	11.0	11.0	236.20	30.0	18.0	10.0	75	**6**
Used to treat Parkinson's disease	**Entacapone ESI-**	**ESI-**	304.05	66.05	15.0	22.0	10.0	184.00	15.0	31.0	28.0	75	**6**
Psychiatric drugs-metabolite	**Desmethylcitalopram**	**ESI+**	352.05	311.00	-29.0	-8.0	-15.0	108.95	-18.0	-34.0	-22.0	10	**3**
Psychiatric drugs-metabolite	**O-Desmethylvenlafaxine**	**ESI+**	264.00	58.00	-20.0	-25.0	-20.0	246.05	-21.0	-13.0	-17.0	10	**4**
Used to treat Parkinson's disease	**R(-)-Deprenyl (Selegiline)**	**ESI+**	188.00	91.00	-20.0	-25.0	-20.0	119.0	-20.0	-15.0	-20.0	10	**3**
Pshychiatric drugs	**Diazepam**	**ESI+**	284.95	153.90	-23.0	-29.0	-10.0	192.95	-23.0	-33.0	-23.0	10	**2**
Psychiatric drugs-metabolite	**Didemethylcitalopram**	**ESI+**	297.00	108.95	-25.0	-23.0	-10.0	262.00	-23.0	-15.0	-18.0	10	**3**
Used to treat Alzheimer's disease	**Donepezil**	**ESI+**	380.15	90.95	-20.0	-40.0	-20.0	243.15	-15.0	-28.0	-17.0	10	**3**
Psychiatric drugs-metabolite	**10,11-Epoxycarbamazepine**	**ESI+**	253.00	179.95	-21.0	-27.0	-12.0	235.90	-21.0	-12.0	-16.0	10	**1**
Psychiatric drugs	**Fluoxetine**	**ESI+**	309.95	44.00	-25.0	-14.0	-18.0		Note 2			10	**3**
Used to treat Alzheimer's disease	**Galantamine**	**ESI+**	288.00	213.00	-23.0	-24.0	-21.0	198.05	-23.0	-34.0	-13.0	10	**3**
Psychiatric drugs-metabolite	**Norfluoxetine**	**ESI+**	296.00	134.0	-23.0	-8.0	-13.0	30.20	-24.0	-15.0	-12.0	10	**3**
Psychiatric drugs	**Paroxetine**	**ESI+**	330.00	70.0	-27.0	-34.0	-14.0	44.00	-37.0	-28.0	-14.0	10	**3**
Used to treat Parkinson's disease	**Pramipexole**	**ESI+**	211.90	152.95	-20.0	-15.0	-20.0	111.05	-17.0	-29.0	-10.0	10	**3**
Used to treat Parkinson's disease	**Safinamide**	**ESI+**	302.95	215.00	-20.0	-10.0	-20.0	108.95	-20.0	-25.0	-20.0	10	**3**
Psychiatric drugs	**Sertraline**	**ESI+**	305.95	158.90	-15.0	-26.0	-15.0	274.95	-25.0	-13.0	-19.0	10	**3**
Used to treat Parkinson's disease	**Rasagiline**	**ESI+**	172.00	117.00	-20.0	-15.0	-20.0	56.00	-14.0	-7.0	-19.0	10	**3**
Used to treat Alzheimer's and Parkinson's disease	**Rivastigmine**	**ESI+**	251.00	206.00	-20.0	-15.0	-20.0	86.0	-20.0	-25.0	-20.0	10	**3**
Used to treat Parkinson's disease	**Ropinirole**	**ESI+**	261.15	114.05	-20.0	-20-0	-20.0	86.05	-20.0	-35.0	-20.0	10	**3**
Used to treat Parkinson's disease	**Rotigotine**	**ESI+**	315.15	147.00	-20.0	-25.0	-20.0	107.00	-11.0	-48.0	-20.0	10	**3**
Psychiatric drugs	**Trazodone**	**ESI+**	372.05	175.95	-19.0	-26.0	-11.0	147.95	-19.0	-40.0	-15.0	10	**3**
Psychiatric drugs	**Venlafaxine**	**ESI+**	278.10	58.00	-20.0	-25.0	-20.0	260.05	-23.0	-14.0	-12.0	10	**4**
**ILIS**	**Carbamazepine-d10 (ILIS 1)**	**ESI+**	246.95	204.10	-18.0	-22.0	-23.0		Note 2			10	**-**
	**Diazepam-d5 (ILIS 2)**	**ESI+**	289.90	154.05	-23.0	-31.0	-10.0-		Note 2			10	**-**
	**Fluoxetine-d5 (ILIS 3)**	**ESI+**	315.05	44.05	-25.0	-15.0	-20.0		Note 2			10	**-**
	**Venlafaxine-d6 (ILIS 4)**	**ESI+**	283.80	64.05	-20.0	-25.0	-20.0		Note 2			10	**-**
	**Caffeine $^{13}C_3$ (ILIS 5)**	**ESI+**	197.95	140.05	-22.0	-22.0	-14.0		Note 2			10	**-**
	**Ibuprofen d3 (ILIS 6)**	**ESI-**	208.00	164.00	21.0	10.0	23.0		Note 2			75	**-**

Note 1: Pharmaceuticals are organized in the table by alphabetic order, Note 2: For the isotopically labeled internal standards only one transition is needed.

Table S3. Retention time, regression (equation and determination coefficient (R)), detection and quantitation limits (LOD and LOQ) for each transition, and ion ratio for each pharmaceutical.

Ionization mode	Pharmaceutical	MRM	Retention time			Regression			LOD	LOQ	Ion Ratio				
			Average (min)	RSD (%)	n	Equation	R²	R	(µg/L)	(µg/L)	Average	RSD (%)	n	-20% Ion ratio	+ 20% Ion ratio
**ESI+**	Amantadine	Quantitation	2.386	0.20	33	Y = (0.0453376) X + (0.0504369)	0.99989	0.99994	0.18	0.09	4.63	4.39	25	3.70	5.55
		Qualifier	2.386	0.19	33	Y = (0.00963938) X + (0.0206266)	0.99893	0.99946	0.03	0.60					
**ESI+**	Apomorphine	Quantitation	2.388	0.16	33	Y = (0.0161022) X + (0.0319779)	0.99948	0.99974	0.03	0.10	1.23	2.50	24	0.99	1.48
		Qualifier	2.388	0.20	33	Y = (0.0132965) X + (0.0231884)	0.99950	0.99975	0.02	0.07					
**ESI+**	Benserazide	Quantitation	0.820	1.26	30	Y = (0.00461856) X + (-0.00921162)	0.99591	0.99795	0.22	0.72	2.01	4.44	14	1.61	2.41
		Qualifier	0.820	0.72	29	Y = (0.00183634) X + (-0.00179317)	0.99929	0.99964	0.30	1.01					
**ESI+**	Caffeine	Quantitation	2.373	0.16	33	Y = (0.0153888) X + (-0.0178585)	0.99947	0.99973	1.07	3.58	1.11	3.16	28	0.89	1.33
		Qualifier	2.373	0.21	33	Y = (0.0132080) X + (-0.00592861)	0.99989	0.99995	2.48	8.27					
**ESI+**	Carbamazepine	Quantitation	3.051	0.14	33	Y = (0.122604) X + (-0.0310019)	0.99979	0.99990	0.11	0.36	7.25	2.47	20	5.80	8.69
		Qualifier	3.051	0.15	33	Y = (0.0167452) X + (0.00860501)	0.99993	0.99996	0.01	0.02					
**ESI+**	Carbidopa, PEAK 1	Quantitation	0.830	2.27	33	Y = (0.00562929) X + (-0.0196927)	0.99866	0.99933	9.14	30.5	1.56	2.87	18	1.25	1.87
		Qualifier	0.830	2.21	33	Y = (0.00368878) X + (-0.0110467)	0.99864	0.99932	19.2	64.1					
**ESI+**	Carbidopa, PEAK 2	Quantitation	2.090	0.54	33	Y = (0.00450814) X + (-0.00116717)	0.99954	0.99977	2.80	9.32	1.49	5.15	21	1.20	1.79
		Qualifier	2.090	1.09	33	Y = (0.00292583) X + (-0.00126132)	0.99987	0.99994	10.7	35.7					
**ESI+**	Citalopram	Quantitation	2.808	0.25	33	Y = (0.0442799) X + (0.0880598)	0.99648	0.99824	0.02	0.07	3.54	3.80	28	2.83	4.25
		Qualifier	2.808	0.25	33	Y = (0.0125844) X + (0.0259496)	0.99720	0.99860	0.03	0.10					
**ESI+**	Citalopram N-oxide	Quantitation	2.837	0.18	33	Y = (0.0353610) X + (0.0397188)	0.99983	0.99991	0.01	0.02	1.12	3.40	18	0.89	1.34
		Qualifier	2.837	0.19	33	Y = (0.0295560) X + (0.117298)	0.99921	0.99961	0.02	0.08					
**ESI-**	Citalopram propionic acid	Quantitation	2.155	0.64	33	Y = (0.0216267) X + (0.00813724)	0.99993	0.99997	0.14	0.47	1.38	2.15	29	1.10	1.66
		Qualifier	2.155	0.66	33	Y = (0.0155848) X + (0.0107721)	0.99993	0.99996	0.02	0.06					
**ESI+**	Desmethylcitalopram	Quantitation	2.782	0.23	33	Y = (0.0354190) X + (0.0354633)	0.99965	0.99983	1.03	3.42	3.64	5.81	23	2.91	4.37
		Qualifier	2.782	0.27	33	Y = (0.00908106) X + (0.0257226)	0.99964	0.99982	0.03	0.10					
**ESI+**	*O*-Desmethylvenlafaxine	Quantitation	2.428	0.10	33	Y = (0.0365291) X + (-0.0193206)	0.99955	0.99977	0.08	0.25	4.27	1.46	27	3.42	5.12
		Qualifier	2.428	0.13	33	Y = (0.00871025) X + (-0.00355572)	0.99946	0.99973	0.63	2.10					
**ESI+**	R(-)-Deprenyl (Selegiline)	Quantitation	2.494	0.21	33	Y = (0.128105) X + (0.253603)	0.99963	0.99982	0.03	0.10	2.71	1.56	26	2.16	3.25
		Qualifier	2.494	0.21	33	Y = (0.0466670) X + (0.0970582)	0.99918	0.99959	0.07	0.23					
**ESI+**	Diazepam	Quantitation	3.447	0.14	33	Y = (0.0473222) X + (-0.000968902)	0.99993	0.99997	0.32	1.07	1.24	2.53	25	0.99	1.48
		Qualifier	3.447	0.14	33	Y = (0.0385476) X + (-0.0100235)	0.99971	0.99986	0.08	0.27					
**ESI+**	Didemethylcitalopram	Quantitation	2.756	0.25	33	Y = (0.0119227) X + (-0.00876088)	0.99973	0.99986	0.62	2.07	1.22	3.46	27	0.98	1.46
		Qualifier	2.756	0.23	33	Y = (0.0102706) X + (-0.0107376)	0.99943	0.99971	0.20	0.65					
**ESI+**	Donepezil	Quantitation	2.726	0.20	33	Y = (0.0646998) X + (0.131358)	0.99975	0.99987	0.02	0.07	8.05	4.69	16	6.44	9.65
		Qualifier	2.726	0.21	33	Y = (0.00853614) X + (-7.03144e-005)	0.99998	0.99999	0.04	0.14				0.99	1.48
**ESI+**	10,11-Epoxycarbamazepine	Quantitation	2.839	0.16	33	Y = (0.0436250) X + (-0.0500290)	0.99971	0.99985	0.04	0.12	1.15	2.57	30	0.92	1.38
		Qualifier	2.839	0.18	33	Y = (0.0381065) X + (-0.0366129)	0.99952	0.99976	4.39	14.63					
**ESI-**	Entacapone	Quantitation	2.104	1.95	30	Y = (0.0148894) X + (-0.0593280)	0.99895	0.99948	8.54	28.47	4.56	5.52	16	3.65	5.47
		Qualifier	2.104	2.01	32	Y = (0.00313723) X + (-0.00770596)	0.99726	0.99863	10.49	34.98					
**ESI+**	Fluoxetine	Quantitation	2.977	0.31	33	Y = (0.0952901) X + (-0.00482390)	0.99987	0.99991	0.01	0.03					
		Qualifier	**Note 1**												
**ESI+**	Galantamine	Quantitation	2.206	0.29	33	Y = (0.0198837) X + (0.0383596)	0.99951	0.99976	0.02	0.08	1.64	1.79	24	1.31	1.97
		Qualifier	2.206	0.32	33	Y = (0.0122658) X + (0.0192965)	0.99979	0.99989	0.01	0.04					
**ESI+**	Norfluoxetine	Quantitation	2.945	0.32	33	Y = (0.00916582) X + (-0.00731686)	0.99987	0.99993	0.09	0.29	1.19	3.54	24	0.95	1.43
		Qualifier	2.945	0.25	33	Y = (0.00756993) X + (-0.00302123)	0.99992	0.99996	0.08	0.28					

(cont. Table S3)

Ionization mode	Pharmaceutical	MRM	Retention time Average (min)	Retention time RSD (%)	n	Regression Equation	R^2	R	LOD (µg/L)	LOQ (µg/L)	Ion Ratio Average	Ion Ratio RSD (%)	n	-20% Ion ratio	+ 20% Ion ratio
**ESI+**	Paroxetine	Quantitation	2.876	0.24	33	Y = (0.0271489) X + (0.00260759)	0.99982	0.99991	0.13	0.44	1.36	1.68	23	1.09	1.63
		Qualifier	2.876	0.23	33	Y = (0.0198245) X + (-0.000336545)	0.99998	0.99999	0.15	0.52					
**ESI+**	Pramipexole	Quantitation	0.822	1.66	30	Y = (0.0114714) X + (-0.109256)	0.99961	0.99980	20.2	67.3	4.07	2.28	17	3.26	4.88
		Qualifier	0.822	2.34	30	Y = (0.00292274) X + (-0.0284604)	0.99977	0.99989	24.0	80.1					
**ESI+**	Rasagiline	Quantitation	2.370	0.20	33	Y = (0.0834670) X + (0.287126)	0.99927	0.99963	0.01	0.02	1.42	3.83	25	1.14	1.70
		Qualifier	2.370	0.20	33	Y = (0.0617683) X + (0.0957436)	0.99986	0.99993	0.02	0.06					
**ESI+**	Rivastigmine	Quantitation	2.488	0.25	33	Y = (0.116837) X + (0.00320035)	0.99983	0.99992	0.05	0.17	1.31	2.01	29	1.05	1.58
		Qualifier	2.488	0.25	33	Y = (0.0889442) X + (0.0261316)	0.99973	0.99986	0.02	0.07					
**ESI+**	Ropinirole	Quantitation	2.411	0.13	33	Y = (0.181141) X + (0.110708)	0.99997	0.99998	0.02	0.06	6.27	1.28	24	5.02	7.53
		Qualifier	2.411	0.16	33	Y = (0.0291694) X + (0.0262145)	0.99986	0.99993	0.01	0.04					
**ESI+**	Rotigotine	Quantitation	2.817	0.24	33	Y = (0.109587) X + (0.0337510)	0.99878	0.99939	0.01	0.02	3.15	1.74	29	2.52	3.78
		Qualifier	2.817	0.26	33	Y = (0.0350049) X + (0.00987641)	0.99957	0.99978	0.02	0.06					
**ESI+**	Safinamide	Quantitation	2.713	0.18	33	Y = (0.116952) X + (-0.0470246)	0.99972	0.99986	0.14	0.46	1.43	2.61	31	1.14	1.71
		Qualifier	2.713	0.20	33	Y = (0.0879782) X + (-0.0937930)	0.99964	0.99982	0.10	0.33					
**ESI+**	Sertraline	Quantitation	3.012	0.45	33	Y = (0.0502044) X + (0.0339637)	0.99992	0.99996	0.01	0.04	1.43	3.14	20	1.14	1.71
		Qualifier	3.012	0.44	33	Y = (0.0344204) X + (0.0599422)	0.99966	0.99983	0.02	0.06					
**ESI+**	Trazodone	Quantitation	2.679	0.23	33	Y = (0.0242538) X + (0.172381)	0.99965	0.99982	0.05	0.17	1.11	1.83	30	0.89	1.33
		Qualifier	2.679	0.23	33	Y = (0.0219858) X + (0.166551)	0.99962	0.99981	0.12	0.38					
**ESI+**	Venlafaxine	Quantitation	2.649	0.25	33	Y = (0.220887) X + (-0.202028)	0.99968	0.99984	0.16	0.12	5.01	2.80	22	4.01	6.01
		Qualifier	2.649	0.26	33	Y = (0.0429576) X + (-0.0240453)	0.99975	0.99987	0.04	0.55					

Pharmaceuticals are organized in the table by alphabetic order

Note 1: Only one transition could be recorded due to their poor fragmentation

Table S4. Pharmaceuticals used in Neurodegenerative Treatments- bibliography search in the extraction and analysis.

**Compounds**		Sample	**Extraction**		**Recovery**	**Analysis**	**Concentration**	**Reference**
Amantadine	Two antiviral drugs	WWTP influents:<br>-primary influent<br><br>WWTP effluents:<br>-primary effluent,<br>-secondary effluent,<br>-tertiary effluent	SPE<br><br>Clean-up	Oasis1 HLB (200 mg, 6 mL)/ 200 mL (pH = 4), Waters<br><br>Sep-Pak plus NH2 (360 mg)	Primary WWTP effluent: 72%<br><br>Secondary WWTP effluent: 81%	UPLC system coupled to a Quattro Micro API mass spectrometer with electrospray ionization (Waters)	Japan, Kyoto<br>WWTP Influent: 230-580 ng/L<br>WWTP primary effluent: 207–470 ng/L<br>WWTP secondary effluent: 125–274 ng/L<br>WWTP tertiary effluent: n.d.–92.7 ng/L	2010<br>Gopal Chandra Ghosh
Amantadine	Four anti-influenza drugs	Surface water<br>-river<br>-tributary water<br><br>WWTP effluent	SPE	Strong-cation solid phase extraction (Bond Elut SCX, 500 mg, 6 mL, Agilent Technologies, Santa Clara, CA)	River or tributary water: 66.2%<br>WWTP Effluent: 48.9%	UPLC system was coupled to a Quattro Micro API MS (Waters Corp., Milford, MA, USA) equipped with an electrospray ionization source	Japan, Kansai<br>WWTP effluent: 22.5–180 ng/L<br>Surface water: 0.9–85.2 ng/L	2013<br>Takashi Azuma
Amantadine	Three anti-influenza drugs<br>-oseltamivir<br>-zanamivir<br>-amantadine<br>-metabolite (oseltamivir carboxylate)	River water<br><br>WWTP Sewage<br>-influent<br>-effluent	SPE	Strong-cation solid phase extraction (Bond Elut SCX, 500 mg, 6 mL Agilent Technologies, Santa Clara, CA)	WWTP influent: 62.9%<br>WWTP effluent: 69.9%<br>River: 75.4%	UPLC system was coupled to a Quattro Micro API MS (Waters Corp., Milford, MA, USA) equipped with an electrospray ionization source	Japan, Kansai<br>WWTP Influent: 105.1–160.9 ng/L<br>WWTP effluent: 89.1–258.4 ng/L<br>River: 7.8–97.6 ng/L	2014<br>Takashi Azuma
Amantadine	64 Pharmaceuticals and metabolites	Source water finished water (6 drinking water purification plants and 2 industrial water purification plants)	SPE		Recovery ratios of the target pharmaceuticals were in the range of 28% to 146%	Liquid chromatography with tandem mass spectrometry (LCeMS/MS);<br>Liquid chromatography with mass spectrometry (LC/MS);<br>Trimethylsilyl derivatization followed by gas chromatography with mass spectrometry (GC/MS).	Japan<br>Source water: n.d.–21 ng/L	2015<br>Dai Simazaki
Amantadine	43 Pharmaceuticals	WWTP influent<br>WWTP effluent	SPE	Oasis 1 HLB (200 mg, 6 mL)/50 mL (pH = 7)	WWTP influents: 110%<br>WWTP effluent: 100%	Surveyor HPLC system (Thermo Finnigan) with with an electrospray ionization. The double-focusing magnetic sector HRMS (Thermo Finnigan) was operated in multiple ion detection (MID) mode for selective target analysis	Belgium, Lede<br>WWTP influent:44–326 ng/L<br>WWTP effluent: 53–592 ng/L	2015<br>Leendert Vergeynst
Amantadine	Three anti-influenza drugs and one prodrug	River water<br><br>WWTP effluent	SPE	Tandem system<br>Bond Elut SCX (500 mg; Agilent Technologies, Santa Clara, CA, USA) followed by an OASIS HLB (60 mg;Waters Corp., Milford, MA, USA)	river water: 52%–94%<br>WWTP effluent: 42%–70%<br>(the range of recovery of all compounds)	UPLC system was coupled to a Quattro Micro API MS (Waters Corp., Milford, MA, USA) equipped with an electrospray ionization source	Japan, Kansai<br>WWTP effluent: 76-232 ng/L<br>River: 5-61 ng/L ng/L	2017<br>Takashi Azuma
Amantadine	450 compounds	WWTP efluent	SPE	Strata X (200 mg) and a mixture of Strata WAX (100 mg), Strata WCX (100 mg), and IsoluteENV+ (150 mg), to achieve sufficient enrichment for a broad range of compounds (neutral, acidic, basic).	Not mentioned	UHPLC system (DionexUltiMate 3000 RSLC, Thermo Fish-erScientific, Germany) interfaced to a QTOF mass spectrometer(MaxisImpact, Bruker Daltonics, Bremen, Germany)<br>UPLC system (Waters, Milford, MA, USA) was interfaced to a hybrid quadrupole-orthogonal acceleration-TOFmass spectrometer (XEVO G2 QTOF, Waters	Greece, Athens<br>WWTP effluent<br>Detected in all samples (7 samples)	2017<br>Ibáñez

						Micromass, Manch-ester, UK), using an orthogonal Z-spray-ESI interface		
Amantadine	484 chemicals	Surface waters<br>Yangtze River Delta	SPE	Not mentioned	Not mentioned	LC-HRMS, LC system coupled via a heated electrospray ion source to a quadrupole orbitrap MS (QExactive Plus, Thermo)	China<br>River: 72.58–883.6 ng/L	2018<br>Ying Peng
Amantadine	Amantadine	Sea water	SPE	MCX (3 cc, 60 mg, Waters, USA)	46.8%–60.1% (for different spiking levels)	UHPLC-MS/MS system comprised an Acquity UHPLC system connected on-line with a Xevo TQ-S micro tandem mass spectrometer (Waters, USA) and coupled with an electrospray ionization source	China<br>Sea water 1.99–2.45 ng/L	2019<br>Yingjiang Xu
Amantadine	>2000 emerging contaminants	WWTP influents<br><br>WWTP effluent	SP+E	200 mg Oasis HLB, 150 mg Isolute ENV+, 100 mg Strata-X-AW and 100 mg Strata-X-CV	Acceptable absolute recovery rates (in the range 57–120 %) were observed for the vast majority of the studied compounds (> 75 % of the total).	UHPLC system,<br>with a HPG-3400 pump (Dionex UltiMate 3000 RSLC, Thermo Fisher Scientific, Germany), interfaced to a QTOF mass spectrometer (Maxis Impact, Bruker Daltonics, Bremen, Germany)	Greece, Athens<br>WWTP influent: 60 ng/L<br>WWTP effluent: 40 ng/L	2020<br>Pablo Gago-Ferrero
Amantadine	74 compounds	Surface water (river waters)<br>WWTP influent<br>WWTP effluent	SPE	Oasis HLB cartridge (6 cc/500 mg,Waters, USA)	WWTP effluent: 79.7–97.5%	Liquid chromatography (LC; Agilent Technologies, Waldbronn, Germany) coupled with a high resolution hybrid quadrupole time-of-flight mass spectrometer (Triple TOF 5600, AB Sciex, Foster City, CA) coupled with electrospray ionization source (ESI).	China<br>River: 11.1–26.7 ng/L<br>WWTP influent: not mentioned<br>WWTP effluent: not mentioned	2020<br>Wei Liu
Amantadine	A total of 178 xenobiotics)	Estuary water<br><br>WWTP effluent<br><br>Ultrapure water	SPE	Chromabond HR-X (85 µm, 55-65 Å, Macherey-Nagel, Düren, Germany) in the top and Sepra ZT-WAX (30 µm, 85 Å) and Sepra ZT-WCX (, 30 µm, 85 Å) from Phenomenex, California, USA in the bottom	Estuary water: 95.2%<br><br>WWTP effluent: 64.2%<br><br>Ultrapure water: 45.7%	UHPLC coupled quadrupole-Orbitrap mass spectrometer equipped with a heated ESI source (Thermo-Fisher Scientific, CA, USA)	Spain<br>Galindo WWTP Effluent: 21 ng/L<br>Bilbao Estuary: n.d.<br>Leitzaran River: n.d.<br>Mustarre River: n.d.<br>Oria River: n.d.	2021<br>González-Gaya, B,
Amantadine	79 compounds	Groundwater<br><br>Surface water<br><br>WWTP effluents	SPE ONLINE	Mixed-mode online solid-phase extraction. A series of sorbents, including C18 substances, hyper cross-linked polymers, cation-exchange resin, anion-exchange resin, and graphitized nonporous carbon, were selected and mixed into a single online cartridge	Ground water: 80%<br><br>Surface water: 82%<br><br>WWTP effluent: 82%	Two sets of MS, QQQ MS and QTOF MS, were applied in this study.<br>Both MS systems were equipped with an Agilent Jet Stream (AJS) electrospray ionization (ESI) source.	India<br>Ground water: 82 ng/L<br>Surface water: 22–39 ng/L<br>WWTP effluent: 400–630 ng/L	2022<br>Jianmin Zou
Amantadine	60 compounds	WWTP influent	SPE	Tandem hydrophilic-lipophilic balance (HLB) and mixed-mode cation exchange (MCX) sorbents (Waters)	NanoPure: 24 %<br><br>WWTP influent: 82%	Agilent 6410 triple quadrupole mass analyzer equipped with a 1200 HPLC system (Palo Alto, CA) coupled with electrospray ionization<br>(ESI).	United States<br>n.d.	2023<br>Lahiruni M. Halwatura
Amantadine	>750 compounds	Surface water and tributaries	spe	Waters Oasis HLB SPE cartridges	Not mentioned	Ultra-high Performance Liquid Chromatography/Quadrupole-	India, Northern India<br>Surface water: 0.4–0.8 ng/L	2023<br>Laura A. Richards

		11 surface water samples				Time-of-Flight Mass Spectrometry (LC/Q-TOF-MS) analysis was performed using a semi-quantitative method on an Agilent Q-TOF (model 6545).		
Amantadine	80 compounds	Surface water:<br>-River<br>R-eservoir	SPE	Oasis HLB SPE cartridges (Waters, 500 mg, 6 cc) (Tetracyclines, macrolides, sulfonamides, quinolones, and antiviral)<br>Oasis MCX SPE cartridges (Waters, 60 mg, 3 cc) (Illicit drugs)	Antiviral recovery ranged from 65.5% to 111.9%<br>Antidepressants: recovery ranged from: 78.8%–107.6%	ACQUITY liquid chromatography and a triple quadrupole mass spectrometer (Xevo T-QS micro, Waters Co., Milford, MA, USA). An electrospray ionization source (positive mode) coupling with multiple-reaction monitoring (MRM)	China<br>Surface water: 0.10–1,084.29 ng/L	2023<br>Miao Chen
Amantadine	database containing >40,000 chemical substances<br><br>(follow the work of 2021, González-Gaya, B, A total of 178 xenobiotics)	WWTP effluent<br>WWTP influent	SPE	500 mg solid-phase extraction (SPE) cartridges consisting of cation exchange (100 mg, ZT-WCX), anion exchange (100 mg, ZT-WAX) and reverse phase (300 mg, HRX) sorbents for effluent samples, and with 250 mg SPE cartridges containing half of the above described amounts for influent samples	Work of González-Gaya, B: 95.2% (Estuary water)<br>64.2 % (WWTP effluent)<br>45.7 % (ultrapure water)<br><br>N. Lopez-Herguedas: 127%	Thermo Scientific Dionex Ulti-Mate 3000 UHPLC coupled to a Thermo Scientific Q Exactive Focus quadrupole Orbitrap mass spectrometer (UHPLC-q-Orbitrap) equipped with a heated electrospray ionization source (HESI, Thermo-Fisher Scientific, CA, USA)	Spain<br>WWTP influent: 15–31 ng/L<br>WWTP effluent: 20–49 ng/L	2023<br>N. Lopez-Herguedas
Amantadine	116 Compounds	Surface water pool samples	SPE	Oasis HLB sorbent (200 mg) at the top, and a mixture of the Bond Elut PPL (150 mg), WAX (100 mg), and WCX (100 mg) sorbents at the bottom	Pool samples: 95.35–101.89%<br><br>Good recoveries were obtained between 70 and 120% for the majority of the selected contaminants.	UHPLC system (Waters, Milford, MA) coupled with a Q-Exactive Orbitrap mass spectrometer (Thermo-Fisher Scientific, Germany) and equipped with heated electrospray ionization (HESI)	Spain<br>No information for each specific compound could be taken<br><br>In general, very low levels of contamination were found.<br>48 compounds of the 116 targeted compounds were detected in at least one of the samples<br>The highest concentrations found were mainly concerning those compounds used frequently in our day-to-day life such as sucralose (sweetener) with the highest concentration of 376.9 ng/Lfound in Taradell followed by caffeine (stimulant), acesulfame or saccharin (sweeteners) with maximum concentrations of 179.4, 130.3 and 119.0 ng/L	2023<br>Olga Gómez-Navarro,
Amantadine	32 compounds	Surface Water<br>WWTP effluent	SPE	Method developed by Olga Gómez-Navarro, 2023:<br>Oasis HLB sorbent (200 mg) at the top, and a mixture of the Bond Elut PPL (150 mg), WAX (100 mg), and WCX (100 mg) sorbents at the bottom	Method developed by Olga Gómez-Navarro, 2023:<br><br>Good recoveries were obtained between 70 and 120% for the majority of the selected contaminants.	UPLC system (Waters. Milford. MA),<br>coupled with an Orbitrap Q-Exactive™ mass spectrometer (Thermo Fischer Scientific, San Jose, CA, USA).	Spain<br>Surface water: 131–5,704 ng/L<br>WWTP effluent: 19,800 ng/L	2024<br>Diana P. Manjarrés-Lopez

Amantadine	697 compounds	Surface water (river(	SPE	HR-X (Macherey Nagel) cartridges containing 200 mg of sorbent (hydrophobic polystyrene divinylbenzene copolymer)	River: 93%	Thermo Ultimate 3000 LC system coupled to a QExactive Plus high-resolution mass spectrometer (Thermo). Separate runs were performed in negative and positive electrospray ionization modes.	Western Kenya<br>River: 4–49 ng/L	2024<br>Isaac Cheruiyot Tanui
Amantadine	> 750 compounds	groundwater, Awash River, tap water, and surface water	SPE online	Solid phase extraction (SPE) was conducted with an automated extraction system using waters Oasis HLB SPE cartridges, which had been conditioned with 6 ml of methanol<br>followed by 6 ml of ultra-high purity water. A 1000 ml water sample was loaded	Not mentioned	Agilent 1290 Infinity II LC coupled with 6546 LC-QTOF.	Ethiopia<br><br>Awash river: n.d.<br>Surface water: n.d.<br>Tap water: 2.5–4.0 ng/L<br>Shallow well: n.d.<br>Deep well: n.d.	2024<br>Kidist Hailu
Amantadine	785 Compounds	Surface waters:<br>River<br>WWTP Effluent	SPE	HR-X cartridges containing 200 mg sorbent (Milford, USA)	Rivers: 93%	Thermo Ultimate 3000 LC system coupled to a QExactive Plus high-resolution mass spectrometer (LC-HRMS, Thermo) with separate runs in negative and positive electrospray<br>ionization modes.	Western Kenya<br>River: 23.78–37.23 ng/L<br>WWTP influent: 4.16–10.69 ng/L<br>WWTP effluent: 5.12–20.88 ng/L	2024<br>Ruth Chepchirchir
Amantadine	2362 compounds	River Basin.<br>Ground Water<br>WWTP influent<br>WWTP effluent	SPE	large volume solid phase extraction (LVSPE) device. Solid phase extraction (SPE) (J2 Scientific) was performed on an Atlantic HLB-H SPE Disk	The generic sample preparation protocols that were used assure satisfactory recovery (typically above 60%) for the majority of the targeted compounds	Gas chromatography/high-resolution mass spectrometry (GC-HRMS) (DFS, Thermo) and liquid chromatography/tandem mass spectrometry (LC-MS/MS) (QTrap 5500, Sciex)	Danube<br>WWTP influent: n.d.–7.40 ng/L<br>WWTP effluent: n.d.–10 ng/L<br>River water: 0.52–7.51 ng/L<br>Groundwater: n.d.	2023<br>Kelsey Ng
Benserazide	2362 compounds	River Basin.<br>Ground Water<br>WWTP influent<br>WWTP effluent	SPE	large volume solid phase extraction (LVSPE) device. Solid phase extraction (SPE) (J2 Scientific) was performed on an Atlantic HLB-H SPE Disk	The generic sample preparation protocols that were used assure satisfactory recovery (typically above 60%) for the majority of the targeted compounds	Gas chromatography/high-resolution mass spectrometry (GC-HRMS) (DFS, Thermo) and liquid chromatography/tandem mass spectrometry (LC-MS/MS) (QTrap 5500, Sciex)	Danube<br>WWTP influent: n.d.<br>WWTP effluent: n.d.<br>River water: 1.80–30.24 ng/L<br>Groundwater: n.d.	2023<br>Kelsey Ng
Donepezil	185 Compounds	WWTP effluents<br>Hospital<br>WWTP influent<br>WWTP effluent		by direct-injection LC-MS-MS<br><br>The procedure used follows the general guidelines indicated in USEPA Method 1694 except for sample preservation agent usage (USEPA, 2007)	Note:<br>Recoveries were performed with two spiking levels in: analytical control aliquot, hospital effluent, WWTP influent, WWTP effluent<br>Results:<br>-analytical control aliquot: 98%<br>-WWTP effluent: 94–108%	LC/MS/MS - Agilent 1290-6460 with an electrospray ionization mode	Average concentrations<br>Hospital effluent: n.d.–50 ng/L<br>WWTP influent: n.d.<br>WWTP effluent: n.d.	2015<br>Tiago S. Oliveira

Donepezil	68 compounds	Natural waters	SPE	Oasis® MCX (60 mg, 3cc) cartridge (Waters, Guyancourt, France) c	Evian® and Vittel® in glass bottles (FranceBoissons, Rueil-Malmaison, France) were used as reference waters during initial development and initial characterization of the method. Satisfactory extraction recoveries >70% were obtained for the majority of analytes Evian® water: 104%	UPLCsystem (Waters, Guyancourt, France). A XevoTQ-MS®triple quadripole mass spectrometer (Waters, Guyan-court, France) equipped with an electrospray ionisation (ESI)	France<br>River water: n.d.	2017<br>V. Brieudes
Donepezil	107 compounds	WWTP effluent	QuEChERS	4 g $MgSO_4$<br>1 g NaCl<br>1 g citric acid monohydrate<br>0.5 g trisodiumcitrate dihydrate	Two concentration levels<br>85–112 % effluent	Agilent 1200 series HPLC system (Agilent Technologies, Foster City, CA, USA) coupled to a hybrid triple quadrupole-linear ion trap-mass spectrometer (5500 QTRAP®LC/MS/MS system, Sciex Instruments, Foster City, CA, USA).	Spain<br>WWTP effluent: n.d.	2021<br>A.B. Martínez-Piernas
Donepezil	80 pharmaceuticals	Coastal waters	SPE	Oasis HLB cartridges (200 mg, Waters Corp, Milford, USA)	Not mentioned	Heated electrospray (HESI) in positive or negative ion modewas used for ionization	Fiji<br>Coastal water: 9.7–180 ng/L	2021<br>Jasha Dehm
			SPE					
Entacapone	53 antimicrobials	Groundwater<br>Surface water<br>WWTP effluent<br>WWTP influent		Oasis® WCX (Waters)	Milli-Q water: 81%<br>WWTP influent: 69%	High performance liquid chromatography coupled with tandem mass spectrometry (HPLC-MS/MS; Exion® LC, Sciex® Triple-Quad 3500).	Sweeden<br>Groundwater: n.d.<br>Surface water: n.d.<br>WWTP effluent: n.d.<br>WWTP influent: n.d.	2024<br>Valentina Ugolini
Galantamine	2362 compounds	River Basin.<br>Ground Water<br>WWTP influent<br>WWTP effluent	SPE	large volume solid phase extraction (LVSPE) device. Solid phase extraction (SPE) (J2 Scientific) was performed on an Atlantic HLB-H SPE Disk	The generic sample preparation protocols that were used assure satisfactory recovery (typically above 60%) for the majority of the targeted compounds	Gas chromatography/high-resolution mass spectrometry (GC-HRMS) (DFS, Thermo) and liquid chromatography/tandem mass spectrometry (LC-MS/MS) (QTrap 5500, Sciex)	Danube<br>WWTP influent: n.d.<br>WWTP effluent: n.d.<br>River water: n.d.<br>Groundwater: n.d.	2023<br>Kelsey Ng
Galantamine	68 compounds	Natural waters	SPE	Oasis® MCX (60 mg, 3cc) cartridge (Waters, Guyancourt, France) c	Evian® and Vittel® in glass bottles (France Boissons, Rueil-Malmaison, France) were used as reference waters during initial development and initial characterization of the method. Satisfactory extraction recoveries >70% were obtained for the majority of analytes Evian® water: 71%	UPLCsystem (Waters, Guyancourt, France). A XevoTQ-MS®triple quadripole mass spectrometer (Waters, Guyan-court, France) equipped with an electrospray ionisation (ESI)	France<br>River water: n.d.	2017<br>V. Brieudes

Rivastigmine	68 compounds	Natural waters	SPE	Oasis® MCX (60 mg, 3cc) cartridge (Waters, Guyancourt, France) c	Evian® and Vittel® in glass bottles (France Boissons, Rueil-Malmaison, France) were used as reference waters during initial development and initial characterization of the method. Satisfactory extraction recoveries >70% were obtained for the majority of analytes<br>Evian® water: 96%	UPLCsystem (Waters, Guyancourt, France). A XevoTQ-MS®triple quadripole mass spectrometer (Waters, Guyan-court, France) equipped with an electrospray ionisation (ESI)	France<br>River water: <LOD–0.79 ng/L	2017<br>V. Brieudes
Rivastigmine	74 compounds	Surface water (river waters)<br>WWTP influent<br>WWTP effluent	SPE	Oasis HLB cartridge (6 cc/500 mg,Waters, USA)	Not mentioned	Liquid chromatography (LC; Agilent Technologies, Waldbronn, Germany) coupled with a high resolution hybrid quadrupole time-of-flight mass spectrometer (Triple TOF 5600, AB Sciex, Foster City, CA) coupled with electrospray ionization source (ESI).	China<br>River: not mentioned<br>WWTP influent: not mentioned<br>WWTP effluent: not mentioned	2020<br>Wei Liu
Rivastigmine	2362 compounds	River Basin.<br>Ground Water<br>WWTP influent<br>WWTP effluent	SPE	large volume solid phase extraction (LVSPE) device. Solid phase extraction (SPE) (J2 Scientific) was performed on an Atlantic HLB-H SPE Disk	The generic sample preparation protocols that were used assure satisfactory recovery (typically above 60%) for the majority of the targeted compounds	Gas chromatography/high-resolution mass spectrometry (GC-HRMS) (DFS, Thermo) and liquid chromatography/tandem mass spectrometry (LC-MS/MS) (QTrap 5500, Sciex)	Danube<br>WWTP influent: n.d.<br>WWTP effluent: n.d.<br>River water: <LOQ<br>Groundwater: n.d.	2023<br>Kelsey Ng
Ropinirole	178 xenobiotics)	Estuary water<br>WWTP effluent<br>Ultrapure water	SPE	Strata HR-X (top), and Strata ZT-WAX and ZT-WCX (bottom) sorbents	Estuary water: 76.5%<br>WWTP effluent: 43.9%<br>Ultrapure water 82.3%	UHPLC coupled quadrupole-Orbitrap mass spectrometer equipped with a heated ESI source (Thermo-Fisher Scientific, CA, USA)	Spain<br>Galindo Effluent: n.d.<br>Bilbao Estuary: n.d.<br>Leitzaran River: n.d.<br>Mustarre River: n.d.<br>Oria River: n.d.	2021<br>González-Gaya, B
Ropinirole	2362 compounds	River Basin.<br>Ground Water<br>WWTP influent<br>WWTP effluent	SPE	large volume solid phase extraction (LVSPE) device. Solid phase extraction (SPE) (J2 Scientific) was performed on an Atlantic HLB-H SPE Disk	The generic sample preparation protocols that were used assure satisfactory recovery (typically above 60%) for the majority of the targeted compounds	Gas chromatography/high-resolution mass spectrometry (GC-HRMS) (DFS, Thermo) and liquid chromatography/tandem mass spectrometry (LC-MS/MS) (QTrap 5500, Sciex)	Danube<br>WWTP influent: n.d.<br>WWTP effluent: n.d.<br>River water: n.d.<br>Groundwater: n.d.	2023<br>Kelsey Ng
Ropinirole	database containing >40,000 chemical substances<br><br>(follow the work of 2021, González-Gaya, B, A total of 178 xenobiotics)	WWTP effluent<br>WWTP influent	SPE	500 mg solid-phase extraction (SPE) cartridges consisting of cation exchange (100 mg, ZT-WCX), anion exchange (100 mg, ZT-WAX) and reverse phase (300 mg, HRX) sorbents for effluent samples, and with 250 mg SPE cartridges containing half of the above described amounts for influent samples	Recovery: 129%	Thermo Scientific Dionex Ulti-Mate 3000 UHPLC coupled to a Thermo Scientific Q Exactive Focus quadrupole Orbitrap mass spectrometer (UHPLC-q-Orbitrap) equipped with a heated electrospray ionization source (HESI, Thermo-Fisher Scientific, CA, USA)	Spain<br>WWTP influent: 6 ng/L<br>WWTP effluent: 9–29 ng/L	2023<br>N. Lopez-Herguedas

Caffeine	185 Compounds	WWTP effluents<br>Hospital<br>WWTP influent<br>WWTP effluent	-	by direct-injection LC-MS-MS<br><br>The procedure used follows the general guidelines indicated in USEPA Method 1694 except for sample preservation agent usage (USEPA, 2007)	Note:<br>Recoveries were performed with two spiking levels in: analytical control aliquot, hospital effluent, WWTP influent, WWTP effluent<br>Results:<br>-analytical control aliquot: 101-102%<br>-WWTP effluent: 92-175%	LC/MS/MS - Agilent 1290-6460 with an electrospray ionization mode	Average concentrations<br>Hospital effluent: 45,740–325,000 ng/L<br>WWTP influent: 73,960–83,330 ng/L<br>WWTP effluent: n.d.–11,650 ng/L	2015<br>Tiago S. Oliveira
Caffeine	484 chemicals	Surface waters<br>Yangtze River Delta	SPE	Not mentioned	Not mentioned	LC-HRMS, LC system coupled via a heated electrospray ion source to a quadrupole orbitrap MS (QExactive Plus, Thermo)	China<br>n.d.	2018<br>Ying Peng
Caffeine	>2000 emerging contaminants	WWTP influents<br>WWTP effluent	SP+E	200 mg Oasis HLB, 150 mg Isolute ENV+, 100 mg Strata-X-AW and 100 mg Strata-X-CV	Acceptable absolute recovery rates (in the range 57–120 %) were observed for the vast majority of the studied compounds (> 75 % of the total).	UHPLC system,<br>with a HPG-3400 pump (Dionex UltiMate 3000 RSLC, Thermo Fisher Scientific, Germany), interfaced to a QTOF mass spectrometer (Maxis Impact, Bruker Daltonics, Bremen, Germany)	Greece, Athens<br>WWTP influent: 9,600 ng/L<br>WWTP effluent: 3,000 ng/L	2020<br>Pablo Gago-Ferrero
Caffeine	178 xenobiotics)	Estuary water<br>WWTP effluent<br>Ultrapure water	SPE	Strata HR-X (top), and Strata ZT-WAX and ZT-WCX (bottom) sorbents	Estuary water: 83.4%<br>WWTP effluent: 87.9%<br>Ultrapure water 57.3%	UHPLC coupled quadrupole-Orbitrap mass spectrometer equipped with a heated ESI source (Thermo-Fisher Scientific, CA, USA)	Spain<br>Galindo Effluent: n.d<br>Bilbao Estuary: 28 ng/L<br>Leitzaran River=n.d.<br>Mustarre River: 9.3 ng/L<br>Oria River: 12 ng/L	2021<br>González-Gaya, B
Caffeine	60 compounds	WWTP influent	SPE	Tandem hydrophilic-lipophilic balance (HLB) and mixed-mode cation exchange (MCX) sorbents (Waters)	NanoPure: 96%<br><br>WWTP influent: 93%	Agilent 6410 triple quadrupole mass analyzer equipped with a 1200 HPLC system (Palo Alto, CA) coupled with electrospray ionization<br>(ESI).	United States<br>WWTP influente: 15,900–40,500 ng/L	2023<br>Lahiruni M. Halwatura
Caffeine	>750 compounds	Surface water and tributaries<br><br>11 surface water samples	spe	Waters Oasis HLB SPE cartridges	Not mentioned	Ultra-high Performance Liquid Chromatography/Quadrupole-Time-of-Flight Mass Spectrometry (LC/Q-TOF-MS) analysis was performed using a semi-quantitative method on an Agilent Q-TOF (model 6545).	India,Northern India<br>Surface water: n.d.	2023<br>Laura A. Richards
Caffeine	database containing >40,000 chemical substances<br><br>(follow the work of 2021, González-Gaya, B, A total of 178 xenobiotics)	WWTP effluent<br>WWTP influent	SPE	500 mg solid-phase extraction (SPE) cartridges consisting of cation exchange (100 mg, ZT-WCX), anion exchange (100 mg, ZT-WAX) and reverse phase (300 mg, HRX) sorbents for effluent samples, and with 250 mg SPE cartridges containing half of the above described	Work of González-Gaya, B:<br>83.4% (Estuary water)<br>87.9 % (WWTP effluent)<br>57.3 % (ultrapure water)<br><br>Lopez-Herguedas: 95%	Thermo Scientific Dionex Ulti-Mate 3000 UHPLC coupled to a Thermo Scientific Q Exactive Focus quadrupole Orbitrap mass spectrometer (UHPLC-q-Orbitrap) equipped with a heated electrospray ionization source (HESI, Thermo-Fisher Scientific, CA, USA)	Spain<br>WWTP influent: 9,587–284,801 ng/L<br>WWTP effluent: <LOD	2023<br>N. Lopez-Herguedas

				amounts for influent samples				
Caffeine	116 Compounds	Surface water pool samples	SPE	Oasis HLB sorbent (200 mg) at the top, and a mixture of the Bond Elut PPL (150 mg), WAX (100 mg), and WCX (100 mg) sorbents at the bottom	Pool samples: 113.70–145.82%<br><br>Good recoveries were obtained between 70 and 120% for the majority of the selected contaminants	ACQUITY UHPLC system (Waters, Milford, MA) coupled with a Q-Exactive Orbitrap mass spectrometer (Thermo-Fisher Scientific, Germany) and equipped with heated electrospray ionization (HESI)	Spain<br>No information for each specific compound could be taken<br><br>In general, very low levels of contamination were found.<br>48 compounds of the 116 targeted compounds were detected in at least one of the samples<br>The highest concentrations found were mainly concerning those compounds used frequently in our day-to-day life such as sucralose (sweetener) with the highest concentration of 376.9 ng/L found in Taradell followed by caffeine (stimulant), acesulfame or saccharin (sweeteners) with maximum concentrations of 179.4, 130.3 and 119.0 ng/L	2023<br>Olga Gómez-Navarro,
Caffeine	2362 compounds	River Basin.<br>Ground Water<br>WWTP influent<br>WWTP effluent	SPE	large volume solid phase extraction (LVSPE) device. Solid phase extraction (SPE) (J2 Scientific) was performed on an Atlantic HLB-H SPE Disk	The generic sample preparation protocols that were used assure satisfactory recovery (typically above 60%) for the majority of the targeted compounds	Gas chromatography/high-resolution mass spectrometry (GC-HRMS) (DFS, Thermo) and liquid chromatography/tandem mass spectrometry (LC-MS/MS) (QTrap 5500, Sciex)	Danube<br>WWTP influent: 896-3944 ng/L<br>WWTP effluent: 6.5-345 ng/L<br>River water: 1.19-70.42<br>Groundwater: 0.39-3.80 ng/L	2023<br>Kelsey Ng
Caffeine	32 compounds	Surface Water<br>WWTP effluent	SPE	Method developed by Olga Gómez-Navarro, 2023:<br>Oasis HLB sorbent (200 mg) at the top, and a mixture of the Bond Elut PPL (150 mg), WAX (100 mg), and WCX (100 mg) sorbents at the bottom	Method developed by Olga Gómez-Navarro, 2023:<br><br>Good recoveries were obtained between 70 and 120% for the majority of the selected contaminants.	UPLC system (Waters. Milford. MA),<br>coupled with an Orbitrap Q-Exactive™ mass spectrometer (Thermo Fischer Scientific, San Jose, CA, USA).	Spain<br>Surface water: 34.9–262 ng/L<br>WWTP effluent 202 ng/L	2024<br>Diana P. Manjarrés-Lopez
Caffeine	697 compounds	Surface water (river(	SPE	HR-X (Macherey Nagel) cartridges containing 200 mg of sorbent (hydrophobic polystyrene divinylbenzene copolymer)	River: 88%	Thermo Ultimate 3000 LC system coupled to a QExactive Plus high-resolution mass spectrometer (Thermo). Separate runs were performed in negative and positive electrospray ionization modes.	Western Kenya<br>River: 2–7 ng/L	2024<br>Isaac Cheruiyot Tanui
Caffeine	785 Compounds	Surface waters:<br>River<br>WWTP Influent<br>WWTP Effluent	SPE	HR-X cartridges containing 200 mg sorbent (Milford, USA)	Rivers: 88%	Thermo Ultimate 3000 LC system coupled to a QExactive Plus high-resolution mass spectrometer (LC-HRMS, Thermo) with separate runs in negative and positive electrospray ionization modes.	Western Kenya<br>River: 27.62–666.36 ng/L<br>WWTP influent: n.d.–830,659 ng/L<br>WWTP effluent: n.d.–170,088 ng/L	2024<br>Ruth Chepchirchir
Carbamazepine	64 Pharmaceuticals and metabolites	Source water finished water (6 drinking water purification plants	SPE		Recovery ratios of the target pharmaceuticals were in the range of 28% to 146%	Liquid chromatography with tandem mass spectrometry (LCeMS/MS);<br>Liquid chromatography with mass spectrometry (LC/MS);	Japan<br>n.d.–10 ng/L	2015<br>Dai Simazaki

		and 2 industrial water purification plants)				Trimethylsilyl derivatization followed by gas chromatography with mass spectrometry (GC/MS)		
Carbamazepine	43 Pharmaceuticals	Wastewater influent<br>Wastewater effluent		SPE - Oasis 1 HLB (200 mg, 6 mL)/50 mL (pH = 7)	WWTP influents: 110%<br>WWTP effluent: 100%	Surveyor HPLC system (Thermo Finnigan) with with an electrospray ionization. The double-focusing magnetic sector HRMS (Thermo Finnigan) was operated in multiple ion detection (MID) mode for selective target analysis	Belgium, Lede<br><br>WWTP influent:462–708 ng/L<br>WWTP effluent: 460–741 ng/L	2015<br>Leendert Vergeynst
Carbamazepine	185 Compounds	WWTP effluents<br>Hospital<br>WWTP influent<br>WWTP effluent	-	by direct-injection LC-MS-MS<br><br><br>The procedure used follows the general guidelines indicated in USEPA Method 1694 except for sample preservation agent usage (USEPA, 2007)	Note:<br>Recoveries were performed with two spiking levels in: analytical control aliquot, hospital effluent, WWTP influent, WWTP effluent<br><br>Results:<br>-analytical control aliquot: 96–102%<br>-Hospital effluent:92—122%<br>-WWTP influent: 86–116%<br>-WWTP effluent: 67–127%	LC/MS/MS - Agilent 1290-6460 with an electrospray ionization mode	Average concentrations<br>Hospital effluent: 20–620 ng/L<br>WWTP influent: 160–570 ng/L<br>WWTP effluent: 160v580 ng/L	2015<br>Tiago S. Oliveira
Carbamazepine	68 compounds	Natural waters	SPE	Oasis® MCX (60 mg, 3cc) cartridge (Waters, Guyancourt, France) c	Evian® and Vittel® in glass bottles (France Boissons, Rueil-Malmaison, France) were used as reference waters during initial development and initial characterization of the method. Satisfactory extraction recoveries >70% were obtained for the majority of analytes Evian® water: 111%	UPLCsystem (Waters, Guyancourt, France). A XevoTQ-MS®triple quadripole mass spectrometer (Waters, Guyan-court, France) equipped with an electrospray ionisation (ESI)	France<br>River water: 4.75–31.8 ng/L	2017<br>V. Brieudes
Carbamazepine	450 Compounds	WWTP efluent	SPE	Strata X (200 mg) and a mixture of Strata WAX (100 mg), Strata WCX (100 mg), and IsoluteENV+ (150 mg), to achieve sufficient enrichment for a broad range of compounds (neutral, acidic, basic).	Not mentioned	UHPLC system (DionexUltiMate 3000 RSLC, Thermo Fish-erScientific, Germany) interfaced to a QTOF mass spectrometer (MaxisImpact, Bruker Daltonics, Bremen, Germany)<br>UPLC system (Waters, Milford, MA, USA) was interfaced to a hybrid quadrupole-orthogonal acceleration-TOFmass spectrometer (XEVO G2 QTOF, Waters Micromass, Manch-ester, UK), using an orthogonal Z-spray-ESI interface	Greece, Athens<br>Detected in all samples (7 samples)	2017<br>Ibanez

Cabamazepine	484 chemicals	Surface waters<br>Yangtze River Delta	SPE	Not mentioned	Not mentioned	LC-HRMS, LC system coupled via a heated electrospray ion source to a quadrupole orbitrap MS (QExactive Plus, Thermo)	China<br>Surface water: 1.384–14.592 ng/L	2018<br>Ying Peng
Carbamazepine	>2000 emerging contaminants	WWTP influents<br>WWTP effluent	SP+E	200 mg Oasis HLB, 150 mg Isolute ENV+, 100 mg Strata-X-AW and 100 mg Strata-X-CV	Acceptable absolute recovery rates (in the range 57–120 %) were observed for the vast majority of the studied compounds (> 75 % of the total).	UHPLC system,<br>with a HPG-3400 pump (Dionex UltiMate 3000 RSLC, Thermo Fisher Scientific, Germany), interfaced to a QTOF mass spectrometer (Maxis Impact, Bruker Daltonics, Bremen, Germany)	Greece, Athens<br>WWTP influent: 1,610 ng/L<br>WWTP effluent: 1,700 ng/L	2020<br>Pablo Gago-Ferrero
Carbamazepine	107 compounds	WWTP effluent	QuEChERS	4 g MgSO4<br>1 g NaCl<br>1 g citric acid monohydrate<br>0.5 g trisodiumcitrate dihydrate	Two concentration levels<br>83-104 % effluent	Ag ilent 1200 series HPLC system (Agilent Technologies, Foster City, CA, USA) coupled to a hybrid triple quadrupole-linear ion trap-mass spectrometer (5500 QTRAP®LC/MS/MS system, Sciex Instruments, Foster City, CA, USA).	Spain<br>WWTP effluent: 32–116 ng/L	2021<br>A.B. Martínez-Piernas
Carbamazepine	80 pharmaceuticals	Coastal waters	SPE	Oasis HLB cartridges (200 mg, Waters Corp, Milford, USA)	Not mentioned	Heated electrospray (HESI) in positive or negative ion modewas used for ionization	Fiji<br>Coastal water: 1.2–8.9 ng/L	2021<br>Jasha Dehm
Carbamazepine	178 Xenobiotics	Estuary water<br>WWTP effluent<br>Ultrapure water	SPE	Strata HR-X (top), and Strata ZT-WAX and ZT-WCX (bottom) sorbents	Estuary water: 100.9%<br>WWTP effluent: 116.5%<br>Ultrapure water: 68.4%	UHPLC coupled quadrupole-Orbitrap mass spectrometer equipped with a heated ESI source (Thermo-Fisher Scientific, CA, USA)	Spain<br>Galindo Effluent: 31 ng/L<br>Bilbao Estuary: n.d.<br>Leitzaran River: n.d.<br>Mustarre River: <LOD<br>Oria River:; <LOQ	2021<br>González-Gaya, B,
Carbamazepine	79 compounds	Groundwater<br>Surface water<br>WWTP effluents	SPE<br>ONLINE	Mixed-mode online solid-phase extraction. A series of sorbents, including C18 substances, hyper cross-linked polymers, cation-exchange resin, anion-exchange resin, and graphitized nonporous carbon, were selected and mixed into a single online cartridge	Ground water: 90%<br>Surface water: 87%<br>WWTP effluent: 82%	Two sets of MS, QQQ MS and QTOF MS, were applied in this study.<br>Both MS systems were equipped with an Agilent Jet Stream (AJS) electrospray ionization (ESI) source.	India<br>Groundwater: 170 ng/L<br>Surface water: 160–210 ng/L<br>WWTP effluent: 270–460 ng/L	2022<br>Jianmin Zou
Carbamazepine	2362 compounds	River Basin.<br>Ground Water<br>WWTP influent<br>WWTP effluent	SPE	large volume solid phase extraction (LVSPE) device. Solid phase extraction (SPE) (J2 Scientific) was performed on an Atlantic HLB-H SPE Disk	The generic sample preparation protocols that were used assure satisfactory recovery (typically above 60%) for the majority of the targeted compounds	Gas chromatography/high-resolution mass spectrometry (GC-HRMS) (DFS, Thermo) and liquid chromatography/tandem mass spectrometry (LC-MS/MS) (QTrap 5500, Sciex)	Danube<br>WWTP influent: 21–181 ng/L<br>WWTP effluent: 28–343 ng/L<br>River water: 0.13–57.57 ng/L<br>Groundwater: 0.10–0.34 ng/L	2023<br>Kelsey Ng
Carbamazepine	60 compounds	WWTP influent	SPE	Tandem hydrophilic-lipophilic balance (HLB) and mixed-mode cation exchange (MCX) sorbents (Waters)	NanoPure: 70%<br><br>WWTP influent: 103%	Agilent 6410 triple quadrupole mass analyzer equipped with a 1200 HPLC system (Palo Alto, CA) coupled with electrospray ionization<br>(ESI).	United States<br>WWTP influente: 102–376 ng/L	2023<br>Lahiruni M. Halwatura
Carbamazepine	>750 compounds	Surface water and tributaries<br><br>11 surface water samples	spe	Waters Oasis HLB SPE cartridges	Not mentioned	Ultra-high Performance Liquid Chromatography/Quadrupole-Time-of-Flight Mass Spectrometry (LC/Q-TOF-MS) analysis was performed using a semi-	India<br>Surface water: 0.4–11 ng/L	2023<br>Laura A. Richards

						quantitative method on an Agilent Q-TOF (model 6545).		
Carbamazepine	database containing >40,000 chemical substances<br><br>(follow the work of 2021, González-Gaya, B, A total of 178 xenobiotics)	WWTP effluent<br>WWTP influent	SPE	500 mg solid-phase extraction (SPE) cartridges consisting of cation exchange (100 mg, ZT-WCX), anion exchange (100 mg, ZT-WAX) and reverse phase (300 mg, HRX) sorbents for effluent samples, and with 250 mg SPE cartridges containing half of the above described amounts for influent samples	Work of González-Gaya, B:<br>100.9% (Estuary water)<br>116.5 % (WWTP effluent)<br>68.4 % (ultrapure water)<br><br>N. Lopez-Herguedas: 117%	Thermo Scientific Dionex Ulti-Mate 3000 UHPLC coupled to a Thermo Scientific Q Exactive Focus quadrupole Orbitrap mass spectrometer (UHPLC-q-Orbitrap) equipped with a heated electrospray ionization source (HESI, Thermo-Fisher Scientific, CA, USA)	Spain<br>WWTP influent: 20–33 ng/L<br>WWTP effluent: 31176 ng/L	2023<br>N. Lopez-Herguedas,
Carbamazepine	116 Compounds	Surface water pool samples	SPE	Oasis HLB sorbent (200 mg) at the top, and a mixture of the Bond Elut PPL (150 mg), WAX (100 mg), and WCX (100 mg) sorbents at the bottom	Pool samples: 103.70-107.86%<br><br>Good recoveries were obtained between 70 and 120% for the majority of the selected contaminants.	ACQUITY UHPLC system (Waters, Milford, MA) coupled with a Q-Exactive Orbitrap mass spectrometer (Thermo-Fisher Scientific, Germany) and equipped with heated electrospray ionization (HESI)	Spain<br>No information for each specific compound could be taken<br><br>In general, very low levels of contamination were found.<br>48 compounds of the 116 targeted compounds were detected in at least one of the samples<br>The highest concentrations found were mainly concerning those compounds used frequently in our day-to-day life such as sucralose (sweetener) with the highest concentration of 376.9 ng/L found in Taradell followed by caffeine (stimulant), acesulfame or saccharin (sweeteners) with maximum concentrations of 179.4, 130.3 and 119.0 ng/L	2023<br>Olga Gómez-Navarro,
Carbamazepine	697 compounds	Surface water (river(	SPE	HR-X (Macherey Nagel) cartridges containing 200 mg of sorbent (hydrophobic polystyrene divinylbenzene copolymer)	River: 94%	Thermo Ultimate 3000 LC system coupled to a QExactive Plus high-resolution mass spectrometer (Thermo). Separate runs were performed in negative and positive electrospray ionization modes.	Western Kenya<br>River: 51–84 ng/L	2024<br>Isaac Cheruiyot Tanui
Carbamazepine	> 750 compounds	groundwater, Awash River, tap water, and surface water	SPE online	Solid phase extraction (SPE) was conducted with an automated extraction system using waters Oasis HLB SPE cartridges, which had been conditioned with 6 ml of methanol followed by 6 ml of ultra-high purity water. A 1000 ml water sample was loaded	Not mentioned	Agilent 1290 Infinity II LC coupled with 6546 LC-QTOF.	Ethiopia<br><br>Awash river: 4.4–5.8 ng/L<br>Surface water: n.d.<br>Tap water: n.d.<br>Shallow well: 2 ng/L<br>Deep well: n.d.	2024<br>Kidist Hailu
Carbamazepine	785 Compounds	Surface waters:<br>River<br>WWTP Influent<br>WWTP Effluent	SPE	HR-X cartridges containing 200 mg sorbent (Milford, USA)	Rivers: 94%	Thermo Ultimate 3000 LC system coupled to a QExactive Plus high-resolution mass spectrometer	Western Kenya<br>River: 0.952–51.83 ng/L<br>WWTP influent: n.d.–310.5 ng/L<br>WWTP effluent: n.d.–160.5 ng/L	2024<br>Ruth Chepchirchir

						(LC-HRMS, Thermo) with separate runs in negative and positive electrospray ionization modes.		
Citalopram	68 compounds	Natural waters	SPE	Oasis® MCX (60 mg, 3cc) cartridge (Waters, Guyancourt, France) c	Evian® and Vittel® in glass bottles (France Boissons, Rueil-Malmaison, France) were used as reference waters during initial development and initial characterization of the method. Satisfactory extraction recoveries >70% were obtained for the majority of analytes Evian® water: 89%	UPLCsystem (Waters, Guyancourt, France). A XevoTQ-MS®triple quadripole mass spectrometer (Waters, Guyan-court, France) equipped with an electrospray ionisation (ESI)	France<br>River water: 0.76–7.52 ng/L	2017<br>V. Brieudes
Citalopram	450 Compounds	WWTP efluent	SPE	Strata X (200 mg) and a mixture of Strata WAX (100 mg), Strata WCX (100 mg), and IsoluteENV+ (150 mg), to achieve sufficient enrichment for a broad range of compounds (neutral, acidic, basic).	Not mentioned	UHPLC system (DionexUltiMate 3000 RSLC, Thermo Fish-erScientific, Germany) interfaced to a QTOF mass spectrometer(MaxisImpact, Bruker Daltonics, Bremen, Germany) UPLC system (Waters, Milford, MA, USA) was interfaced to a hybrid quadrupole-orthogonal acceleration-TOFmass spectrometer (XEVO G2 QTOF, Waters Micromass, Manch-ester, UK), using an orthogonal Z-spray-ESI interface	Greece, Athens<br>Detected in all samples (7 samples)	2017<br>Ibanez
Citalopram	>2000 emerging contaminants	WWTP influents<br>WWTP effluent	SP+E	200 mg Oasis HLB, 150 mg Isolute ENV+, 100 mg Strata-X-AW and 100 mg Strata-X-CV	Acceptable absolute recovery rates (in the range 57–120 %) were observed for the vast majority of the studied compounds (> 75 % of the total).	UHPLC system,<br>with a HPG-3400 pump (Dionex UltiMate 3000 RSLC, Thermo Fisher Scientific, Germany), interfaced to a QTOF mass spectrometer (Maxis Impact, Bruker Daltonics, Bremen, Germany)	Greece, Athens<br>WWTP influent: 700 ng/L<br>WWTP effluent: 500 ng/L	2020<br>Pablo Gago-Ferrero
Citalopram	107 compounds	WWTP effluent	QuEChERS	4 g MgSO4<br>1 g NaCl<br>1 g citric acid monohydrate<br>0.5 g trisodiumcitrate dihydrate	Two concentration levels<br>82–106 % effluent	Ag ilent 1200 series HPLC system (Agilent Technologies, Foster City, CA, USA) coupled to a hybrid triple quadrupole-linear ion trap-mass spectrometer (5500 QTRAP®LC/MS/MS system, Sciex Instruments, Foster City, CA, USA).	Spain<br>WWTP effluent: 82–235 ng/L	2021<br>A.B. Martínez-Piernas
Citalopram	80 pharmaceuticals	Coastal waters	SPE	Oasis HLB cartridges (200 mg, Waters Corp, Milford, USA)	Not mentioned	Heated electrospray (HESI) in positive or negative ion modewas used for ionization	Fiji<br>Coastal water: 7.0-85 ng/L	2021<br>Jasha Dehm
Citalopram	2362 compounds	River Basin.<br>Ground Water<br>WWTP influent<br>WWTP effluent	SPE	large volume solid phase extraction (LVSPE) device. Solid phase extraction (SPE) (J2 Scientific) was	The generic sample preparation protocols that were used assure satisfactory recovery (typically above 60%)	Gas chromatography/high-resolution mass spectrometry (GC-HRMS) (DFS, Thermo) and liquid chromatography/tandem mass spectrometry (LC-MS/MS) (QTrap 5500, Sciex)	Danube<br>WWTP influent: n.d.–30.0 ng/L<br>WWTP effluent: n.d.–4.5 ng/L<br>River water: n.d.<br>Groundwater: n.d.	2023<br>Kelsey Ng

				performed on an Atlantic HLB-H SPE Disk	for the majority of the targeted compounds			
Citalopram	60 compounds	WWTP influent	SPE	Tandem hydrophilic-lipophilic balance (HLB) and mixed-mode cation exchange (MCX) sorbents (Waters)	NanoPure: 93%<br><br>WWTP influent: 46%	Agilent 6410 triple quadrupole mass analyzer equipped with a 1200 HPLC system (Palo Alto, CA) coupled with electrospray ionization (ESI).	United States<br>WWTP influente: 35–178 ng/L	2023<br>Lahiruni M. Halwatura
Citalopram	>750 compounds	Surface water and tributaries<br><br>11 surface water samples	spe	Waters Oasis HLB SPE cartridges	Not mentioned	Ultra-high Performance Liquid Chromatography/Quadrupole-Time-of-Flight Mass Spectrometry (LC/Q-TOF-MS) analysis was performed using a semi-quantitative method on an Agilent Q-TOF (model 6545).	India<br>Surface water: n.d.	2023<br>Laura A. Richards
Citalopram	80 compounds	Surface water:<br>-River<br>R-eservoir	SPE	Oasis HLB SPE cartridges (Waters, 500 mg, 6 cc) (Tetracyclines, macrolides, sulfonamides, quinolones, and antiviral)<br><br>Oasis MCX SPE cartridges (Waters, 60 mg, 3 cc) (Illicit drugs)	Antiviral recovery ranged from 65.5% to 111.9%<br>Antidepressants: recovery ranged from: 78.8%–107.6%	ACQUITY liquid chromatography and a triple quadrupole mass spectrometer (Xevo T-QS micro, Waters Co., Milford, MA, USA). An electrospray ionization source (positive mode) coupling with multiple-reaction monitoring (MRM)	China<br>Surface water: <LOD–1.06 ng/L	2023<br>Miao Chen
Citalopram	116 Compounds	Surface water pool samples	SPE	Oasis HLB sorbent (200 mg) at the top, and a mixture of the Bond Elut PPL (150 mg), WAX (100 mg), and WCX (100 mg) sorbents at the bottom	Pool samples: 53.13–65.42%<br><br>Good recoveries were obtained between 70 and 120% for the majority of the selected contaminants.	ACQUITY UHPLC system (Waters, Milford, MA) coupled with a Q-Exactive Orbitrap mass spectrometer (Thermo-Fisher Scientific, Germany) and equipped with heated electrospray ionization (HESI)	Spain<br>No information for each specific compound could be taken<br><br>In general, very low levels of contamination were found.<br>48 compounds of the 116 targeted compounds were detected in at least one of the samples<br>The highest concentrations found were mainly concerning those compounds used frequently in our day-to-day life such as sucralose (sweetener) with the highest concentration of 376.9 ng/Lfound in Taradell followed by caffeine (stimulant), acesulfame or saccharin (sweeteners) with maximum concentrations of 179.4, 130.3 and 119.0 ng/L	2023<br>Olga Gómez-Navarro
Citalopram	32 compounds	Surface Water<br>WWTP effluent	SPE	Method developed by Olga Gómez-Navarro, 2023:<br>Oasis HLB sorbent (200 mg) at the top, and a mixture of the Bond Elut PPL (150 mg), WAX (100 mg), and WCX (100 mg) sorbents at the bottom	Method developed by Olga Gómez-Navarro, 2023:<br><br>Good recoveries were obtained between 70 and 120% for the majority of the selected contaminants.	UPLC system (Waters. Milford. MA),<br>coupled with an Orbitrap Q-Exactive™ mass spectrometer (Thermo Fischer Scientific, San Jose, CA, USA).	Spain<br>Surface water: 9.10-–180 ng/L<br>WWTP effluent 258 ng/L	2024<br>Diana P. Manjarrés-Lopez
Citalopram	697 compounds	Surface water (river(	SPE	HR-X (Macherey Nagel) cartridges containing 200 mg of sorbent	Not mentioned	Thermo Ultimate 3000 LC system coupled to a QExactive Plus high-resolution mass spectrometer	Western Kenya<br>River: n.d	2024<br>Isaac Cheruiyot Tanui

				(hydrophobic polystyrene divinylbenzene copolymer)		(Thermo). Separate runs were performed in negative and positive electrospray ionization modes.		
Citalopram N-oxide	2362 compounds	River Basin.<br>Ground Water<br>WWTP influent<br>WWTP effluent	SPE	large volume solid phase extraction (LVSPE) device. Solid phase extraction (SPE) (J2 Scientific) was performed on an Atlantic HLB-H SPE Disk	The generic sample preparation protocols that were used assure satisfactory recovery (typically above 60%) for the majority of the targeted compounds	Gas chromatography/high-resolution mass spectrometry (GC-HRMS) (DFS, Thermo) and liquid chromatography/tandem mass spectrometry (LC-MS/MS) (QTrap 5500, Sciex)	Danube<br>WWTP influent: n.d.–5.10 ng/L<br>WWTP effluent: n.d.–13.0 ng/L<br>River water: n.d.<br>Groundwater: n.d.	2023<br>Kelsey Ng
Desmethylcitalopram	185 Compounds	WWTP effluents<br>Hospital<br>WWTP influent<br>WWTP effluent	-	by direct-injection LC-MS-MS<br><br>The procedure used follows the general guidelines indicated in USEPA Method 1694 except for sample preservation agent usage (USEPA, 2007)	Note:<br>Recoveries were performed with two spiking levels in: analytical control aliquot, hospital effluent, WWTP influent, WWTP effluent<br>Results:<br>-analytical control aliquot: 98%<br>-WWTP effluent: 94–100%	LC/MS/MS - Agilent 1290-6460 with an electrospray ionization mode	Average concentrations<br>Hospital effluents: 30–210 ng/L<br>WWTP influent: 10–40 ng/L<br>WWTP effluent: 30–100 ng/L	2015<br>Tiago S. Oliveira
Desmethylcitalopram	68 compounds	Natural waters	SPE	Oasis® MCX (60 mg, 3cc) cartridge (Waters, Guyancourt, France) c	Evian® and Vittel® in glass bottles (France Boissons, Rueil-Malmaison, France) were used as reference waters during initial development and initial characterization of the method. Satisfactory extraction recoveries >70% were obtained for the majority of analytes<br>Evian® water: 86%	UPLCsystem (Waters, Guyancourt, France). A XevoTQ-MS®triple quadripole mass spectrometer (Waters, Guyan-court, France) equipped with an electrospray ionisation (ESI)	France<br>River water: 0.62–5.86 ng/L	2017<br>V. Brieudes
Desmethylcitalopram	2362 compounds	River Basin.<br>Ground Water<br>WWTP influent<br>WWTP effluent	SPE	large volume solid phase extraction (LVSPE) device. Solid phase extraction (SPE) (J2 Scientific) was performed on an Atlantic HLB-H SPE Disk	The generic sample preparation protocols that were used assure satisfactory recovery (typically above 60%) for the majority of the targeted compounds	Gas chromatography/high-resolution mass spectrometry (GC-HRMS) (DFS, Thermo) and liquid chromatography/tandem mass spectrometry (LC-MS/MS) (QTrap 5500, Sciex)	Danube<br>WWTP influent: n.d.<br>WWTP effluent: n.d.<br>River water: n.d.<br>Groundwater: n.d.	2023<br>Kelsey Ng
Desmethylcitalopram	32 compounds	Surface Water<br>WWTP effluent	SPE	Method developed by Olga Gómez-Navarro, 2023:<br>Oasis HLB sorbent (200 mg) at the top, and a mixture of the Bond Elut PPL (150 mg), WAX (100 mg), and WCX (100 mg) sorbents at the bottom	Method developed by Olga Gómez-Navarro, 2023:<br><br>Good recoveries were obtained between 70 and 120% for the majority of the selected contaminants.	UPLC system (Waters. Milford. MA),<br>coupled with an Orbitrap Q-Exactive™ mass spectrometer (Thermo Fischer Scientific, San Jose, CA, USA).	Spain<br>Surface water: <LOD–93.1 ng/L<br>WWTP effluent: 121 ng/L	2024<br>Diana P. Manjarrés-Lopez

O-Desmethylvenlafaxine	185 Compounds	WWTP effluents<br>Hospital<br>WWTP influent<br>WWTP effluent	-	by direct-injection LC-MS-MS<br><br>The procedure used follows the general guidelines indicated in USEPA Method 1694 except for sample preservation agent usage (USEPA, 2007)	Note:<br>Recoveries were performed with two spiking levels in: analytical control aliquot, hospital effluent, WWTP influent, WWTP effluent<br>Results:<br>-analytical control aliquot: 104%<br>-WWTP effluent: 66–130%	LC/MS/MS - Agilent 1290-6460 with an electrospray ionization mode	Average concentrations<br>Hospital effluent: 620–2,500 ng/L<br>WWTP influent: 470–1,480 ng/L<br>WWTP effluent: n.d.–1,870 ng/L	2015<br>Tiago S. Oliveira
O-Desmethylvenlafaxine	68 compounds	Natural waters	SPE	Oasis® MCX (60 mg, 3cc) cartridge (Waters, Guyancourt, France) c	Evian® and Vittel® in glass bottles (France Boissons, Rueil-Malmaison, France) were used as reference waters during initial development and initial characterization of the method.<br>Satisfactory extraction recoveries >70% were obtained for the majority of analytes<br>Evian® water: 87%	UPLCsystem (Waters, Guyancourt, France). A XevoTQ-MS®triple quadripole mass spectrometer (Waters, Guyan-court, France) equipped with an electrospray ionisation (ESI)	France<br>River water: 6.76–33.9 ng/L	2017<br>V. Brieudes
O-Desmethylvenlafaxine	>2000 emerging contaminants	WWTP influents<br>WWTP effluent	SP+E	200 mg Oasis HLB, 150 mg Isolute ENV+, 100 mg Strata-X-AW and 100 mg Strata-X-CV	Acceptable absolute recovery rates (in the range 57–120 %) were observed for the vast majority of the studied compounds (> 75 % of the total).	UHPLC system,<br>with a HPG-3400 pump (Dionex UltiMate 3000 RSLC, Thermo Fisher Scientific, Germany), interfaced to a QTOF mass spectrometer (Maxis Impact, Bruker Daltonics, Bremen, Germany)	Greece, Athens<br>WWTP influent: 890 ng/L<br>WWTP effluent: 1,100 ng/L	2020<br>Pablo Gago-Ferrero
O-Desmethylvenlafaxine	80 compounds	Surface water:<br>-River<br>Reservoir	SPE	Oasis HLB SPE cartridges (Waters, 500 mg, 6 cc) (Tetracyclines, macrolides, sulfonamides, quinolones, and antiviral)<br><br>Oasis MCX SPE cartridges (Waters, 60 mg, 3 cc) (Illicit drugs)	Antiviral recovery ranged from 65.5% to 111.9%<br>Antidepressants: recovery ranged from: 78.8%–107.6%	ACQUITY liquid chromatography and a triple quadrupole mass spectrometer (Xevo T-QS micro, Waters Co., Milford, MA, USA). An electrospray ionization source (positive mode) coupling with multiple-reaction monitoring (MRM)	China<br>Surface water: <LOD–30.04 ng/L	2023<br>Miao Chen
O-Desmethylvenlafaxine	116 Compounds	Surface water pool samples	SPE	Oasis HLB sorbent (200 mg) at the top, and a mixture of the Bond Elut PPL (150 mg), WAX (100 mg), and WCX (100 mg) sorbents at the bottom	Pool samples: 67.49-80.51%<br><br>Good recoveries were obtained between 70 and 120% for the majority of the selected contaminants.	ACQUITY UHPLC system (Waters, Milford, MA) coupled with a Q-Exactive Orbitrap mass spectrometer (Thermo-Fisher Scientific, Germany) and equipped with heated electrospray ionization (HESI)	Spain<br>No information for each specific compound could be taken<br><br>In general, very low levels of contamination were found.<br>48 compounds of the 116 targeted compounds were detected in at least one of the samples<br>The highest concentrations found were mainly concerning those	2023<br>Olga Gómez-Navarro

							compounds used frequently in our day-to-day life such as sucralose (sweetener) with the highest concentration of 376.9 ng/L found in Taradell followed by caffeine (stimulant), acesulfame or saccharin (sweeteners) with maximum concentrations of 179.4, 130.3 and 119.0 ng/L	
O-Desmethylvenlafaxine	2362 compounds	River Basin.<br>Ground Water<br>WWTP influent<br>WWTP effluent	SPE	large volume solid phase extraction (LVSPE) device. Solid phase extraction (SPE) (J2 Scientific) was performed on an Atlantic HLB-H SPE Disk	The generic sample preparation protocols that were used assure satisfactory recovery (typically above 60%) for the majority of the targeted compounds	Gas chromatography/high-resolution mass spectrometry (GC-HRMS) (DFS, Thermo) and liquid chromatography/tandem mass spectrometry (LC-MS/MS) (QTrap 5500, Sciex)	Danube<br>WWTP influent: 2.70–9.60 ng/L<br>WWTP effluent: n.d.<br>River water: n.d.<br>Groundwater: n.d.	2023<br>Kelsey Ng
O-Desmethylvenlafaxine	32 compounds	Surface Water<br>WWTP effluent	SPE	Method developed by Olga Gómez-Navarro, 2023:<br>Oasis HLB sorbent (200 mg) at the top, and a mixture of the Bond Elut PPL (150 mg), WAX (100 mg), and WCX (100 mg) sorbents at the bottom	Method developed by Olga Gómez-Navarro, 2023:<br><br>Good recoveries were obtained between 70 and 120% for the majority of the selected contaminants.	UPLC system (Waters. Milford. MA),<br>coupled with an Orbitrap Q-Exactive™ mass spectrometer (Thermo Fischer Scientific, San Jose, CA, USA).	Spain<br>Surface water: 234–2,077 ng/L<br>WWTP effluent: 2,758 ng/L	2024<br>Diana P. Manjarrés-Lopez
O-Desmethylvenlafaxine	697 compounds	Surface water (river(	SPE	HR-X (Macherey Nagel) cartridges containing 200 mg of sorbent (hydrophobic polystyrene divinylbenzene copolymer)	Not mentioned	Thermo Ultimate 3000 LC system coupled to a QExactive Plus high-resolution mass spectrometer (Thermo). Separate runs were performed in negative and positive electrospray ionization modes.	Western Kenya<br>River: n.d	2024<br>Isaac Cheruiyot Tanui
O-Desmethylvenlafaxine	785 Compounds	Surface waters:<br>River<br>WWTP Influent<br>WWTP Effluent	SPE	HR-X cartridges containing 200 mg sorbent (Milford, USA)	Not mentioned	Thermo Ultimate 3000 LC system coupled to a QExactive Plus high-resolution mass spectrometer (LC-HRMS, Thermo) with separate runs in negative and positive electrospray ionization modes.	Western Kenya<br>River: n.d.<br>WWTP influent: n.d.<br>WWTP effluent: n.d.–4.48 ng/L	2024<br>Ruth Chepchirchir
Diazepam	484 chemicals	Surface waters<br>Yangtze River Delta	SPE	Not mentioned	Not mentioned	LC-HRMS, LC system coupled via a heated electrospray ion source to a quadrupole orbitrap MS (QExactive Plus, Thermo)	China<br>Surface water: ≈2–100 ng/L	2018<br>Ying Peng
Diazepam	>2000 emerging contaminants	WWTP influents<br>WWTP effluent	SP+E	200 mg Oasis HLB, 150 mg Isolute ENV+, 100 mg Strata-X-AW and 100 mg Strata-X-CV	Acceptable absolute recovery rates (in the range 57–120 %) were observed for the vast majority of the studied compounds (> 75 % of the total).	UHPLC system,<br>with a HPG-3400 pump (Dionex UltiMate 3000 RSLC, Thermo Fisher Scientific, Germany), interfaced to a QTOF mass spectrometer (Maxis Impact, Bruker Daltonics, Bremen, Germany)	Greece, Athens<br>WWTP influent: 20 ng/L<br>WWTP effluent: 10 ng/L	2020<br>Pablo Gago-Ferrero
Diazepam	107 compounds	WWTP effluent	QuEChERS	4 g $MgSO_4$<br>1 g NaCl<br>1 g citric acid monohydrate<br>0.5 g trisodiumcitrate dihydrate	Two concentration levels<br>85-100 % effluent	Ag ilent 1200 series HPLC system (Agilent Technologies, Foster City, CA, USA) coupled to a hybrid triple quadrupole-linear ion trap-mass spectrometer (5500	Spain<br>WWTP effluent: n.d.	2021<br>A.B. Martínez-Piernas

						QTRAP®LC/MS/MS system, Sciex Instruments, Foster City, CA, USA).		
Diazepam	178 Xenobiotics)	Estuary water<br>WWTP effluent<br>Ultrapure water	SPE	Strata HR-X (top), and Strata ZT-WAX and ZT-WCX (bottom) sorbents	Estuary: 113.3%<br>WWTP effluent: 111.3%<br>Ultrapure water: 61.9%	UHPLC coupled quadrupole-Orbitrap mass spectrometer equipped with a heated ESI source (Thermo-Fisher Scientific, CA, USA)	Spain<br>Galindo Effluent: 1.4 ng/L<br>Bilbao Estuary: n.d.<br>Leitzaran River:n.d.<br>Mustarre River: n.d.<br>Oria River: n.d.	2021<br>González-Gaya, B
Diazepam	2362 compounds	River Basin.<br>Ground Water<br>WWTP influent<br>WWTP effluent	SPE	large volume solid phase extraction (LVSPE) device. Solid phase extraction (SPE) (J2 Scientific) was performed on an Atlantic HLB-H SPE Disk	The generic sample preparation protocols that were used assure satisfactory recovery (typically above 60%) for the majority of the targeted compounds	Gas chromatography/high-resolution mass spectrometry (GC-HRMS) (DFS, Thermo) and liquid chromatography/tandem mass spectrometry (LC-MS/MS) (QTrap 5500, Sciex)	Danube<br>WWTP influent: 4.20 ng/L<br>WWTP effluent: 4.20–4.90 ng/L<br>River water: n.d.<br>Groundwater: n.d.	2023<br>Kelsey Ng
Diazepam	>750 compounds	Surface water and tributaries<br><br>11 surface water samples	spe	Waters Oasis HLB SPE cartridges	Not mentioned	Ultra-high Performance Liquid Chromatography/Quadrupole-Time-of-Flight Mass Spectrometry (LC/Q-TOF-MS) analysis was performed using a semi-quantitative method on an Agilent Q-TOF (model 6545).	India<br>Surface water: n.d.	2023<br>Laura A. Richards
Diazepam	database containing >40,000 chemical substances<br><br>(follow the work of 2021, González-Gaya, B, A total of 178 xenobiotics)	WWTP effluent<br>WWTP influent	SPE	500 mg solid-phase extraction (SPE) cartridges consisting of cation exchange (100 mg, ZT-WCX), anion exchange (100 mg, ZT-WAX) and reverse phase (300 mg, HRX) sorbents for effluent samples, and with 250 mg SPE cartridges containing half of the above described amounts for influent samples	Work of González-Gaya, B:<br>113.3% (Estuary water)<br>113.3 % (WWTP effluent)<br>61.9 % (ultrapure water)<br><br>N. Lopez-Herguedas: 115%	Thermo Scientific Dionex Ulti-Mate 3000 UHPLC coupled to a Thermo Scientific Q Exactive Focus quadrupole Orbitrap mass spectrometer (UHPLC-q-Orbitrap) equipped with a heated electrospray ionization source (HESI, Thermo-Fisher Scientific, CA, USA)	Spain<br>WWTP influent: n.d.<br>WWTP effluent: n.d.	2023<br>N. Lopez-Herguedas
Diazepam	116 Compounds	Surface water pool samples	SPE	Oasis HLB sorbent (200 mg) at the top, and a mixture of the Bond Elut PPL (150 mg), WAX (100 mg), and WCX (100 mg) sorbents at the bottom	Pool samples: 98.95-101.95%<br><br>Good recoveries were obtained between 70 and 120% for the majority of the selected contaminants.	ACQUITY UHPLC system (Waters, Milford, MA) coupled with a Q-Exactive Orbitrap mass spectrometer (Thermo-Fisher Scientific, Germany) and equipped with heated electrospray ionization (HESI)	Spain<br>No information for each specific compound could be taken<br><br>In general, very low levels of contamination were found.<br>48 compounds of the 116 targeted compounds were detected in at least one of the samples<br>The highest concentrations found were mainly concerning those compounds used frequently in our day-to-day life such as sucralose (sweetener) with the highest concentration of 376.9 ng/Lfound in Taradell followed by caffeine (stimulant), acesulfame or saccharin (sweeteners) with maximum concentrations of 179.4, 130.3 and 119.0 ng/L	2023<br>Olga Gómez-Navarro

Diazepam	32 compounds	Surface Water<br>WWTP effluent	SPE	Method developed by Olga Gómez-Navarro, 2023:<br>Oasis HLB sorbent (200 mg) at the top, and a mixture of the Bond Elut PPL (150 mg), WAX (100 mg), and WCX (100 mg) sorbents at the bottom	Method developed by Olga Gómez-Navarro, 2023:<br><br>Good recoveries were obtained between 70 and 120% for the majority of the selected contaminants.	UPLC system (Waters. Milford. MA),<br>coupled with an Orbitrap Q-Exactive™ mass spectrometer (Thermo Fischer Scientific, San Jose, CA, USA).	Spain<br>Surface water: 1.62–27.9 ng/L<br>WWTP effluent: 11.6 ng/L	2024<br>Diana P. Manjarrés-Lopez
10,11-Epoxycarbamazepine	185 Compounds	WWTP effluents<br>Hospital<br>WWTP influent<br>WWTP effluent	-	by direct-injection LC-MS-MS<br><br>The procedure used follows the general guidelines indicated in USEPA Method 1694 except for sample preservation agent usage (USEPA, 2007)	Note:<br>Recoveries were performed with two spiking levels in: analytical control aliquot, hospital effluent, WWTP influent, WWTP effluent<br><br>Results:<br>-analytical control aliquot:97%<br>-WWTP effluent: 61–95%	LC/MS/MS - Agilent 1290-6460 with an electrospray ionization mode	Average concentrations<br>Hospital effluent: 40–100 ng/L<br>WWTP influent: 30–70 ng/L<br>WWTP effluent: 20–60 ng/L	2015<br>Tiago S. Oliveira
10,11-Epoxycarbamazepine	>2000 emerging contaminants	WWTP influents<br>WWTP effluent	SP+E	200 mg Oasis HLB, 150 mg Isolute ENV+, 100 mg Strata-X-AW and 100 mg Strata-X-CV	Acceptable absolute recovery rates (in the range 57–120 %) were observed for the vast majority of the studied compounds (> 75 % of the total).	UHPLC system,<br>with a HPG-3400 pump (Dionex UltiMate 3000 RSLC, Thermo Fisher Scientific, Germany), interfaced to a QTOF mass spectrometer (Maxis Impact, Bruker Daltonics, Bremen, Germany)	Greece, Athens<br>WWTP influent: 90 ng/L<br>WWTP effluent: 50 ng/L	2020<br>Pablo Gago-Ferrero
10,11-Epoxycarbamazepine	116 Compounds	Surface water pool samples	SPE	Oasis HLB sorbent (200 mg) at the top, and a mixture of the Bond Elut PPL (150 mg), WAX (100 mg), and WCX (100 mg) sorbents at the bottom	Pool samples: 90.12-109.46%<br>Good recoveries were obtained between 70 and 120% for the majority of the selected contaminants.	ACQUITY UHPLC system (Waters, Milford, MA) coupled with a Q-Exactive Orbitrap mass spectrometer (Thermo-Fisher Scientific, Germany) and equipped with heated electrospray ionization (HESI)	Spain<br>No information for each specific compound could be taken<br><br>In general, very low levels of contamination were found.<br>48 compounds of the 116 targeted compounds were detected in at least one of the samples<br>The highest concentrations found were mainly concerning those compounds used frequently in our day-to-day life such as sucralose (sweetener) with the highest concentration of 376.9 ng/Lfound in Taradell followed by caffeine (stimulant), acesulfame or saccharin (sweeteners) with maximum concentrations of 179.4, 130.3 and 119.0 ng/L	2023<br>Olga Gómez-Navarro,
10,11-Epoxycarbamazepine	2362 compounds	River Basin.<br>Ground Water<br>WWTP influent	SPE	large volume solid phase extraction (LVSPE)	The generic sample preparation protocols that were used assure	Gas chromatography/high-resolution mass spectrometry (GC-HRMS) (DFS, Thermo) and liquid	Danube<br>WWTP influent: 1.40–5.40 ng/L<br>WWTP effluent: 2–11 ng/L	2023<br>Kelsey Ng

		WWTP effluent		device. Solid phase extraction (SPE) (J2 Scientific) was performed on an Atlantic HLB-H SPE Disk	satisfactory recovery (typically above 60%) for the majority of the targeted compounds	chromatography/tandem mass spectrometry (LC-MS/MS) (QTrap 5500, Sciex)	River water: n.d.<br>Groundwater: <LOQ–1.40 ng/L	
Fluoxetine	185 Compounds	WWTP effluents<br>Hospital<br>WWTP influent<br>WWTP effluent	-	by direct-injection LC-MS-MS<br><br>The procedure used follows the general guidelines indicated in USEPA Method 1694 except for sample preservation agent usage (USEPA, 2007)	Note:<br>Recoveries were performed with two spiking levels in: analytical control aliquot, hospital effluent, WWTP influent, WWTP effluent<br><br>Results:<br>-analytical control aliquot: 49-96%<br>-Hospital effluent:102-117%<br>-WWTP influent:104–113%<br>-WWTP effluent: 40–112%%	LC/MS/MS - Agilent 1290-6460 with an electrospray ionization mode	Average concentrations<br>Hospital effluent: 20–230 ng/L<br>WWTP influent: 10–80 ng/L<br>WWTP effluent: n.d.–130 ng/L	2015<br>Tiago S. Oliveira
Fluoxetine	68 compounds	Natural waters	SPE	Oasis® MCX (60 mg, 3cc) cartridge (Waters, Guyancourt, France) c	Evian® and Vittel® in glass bottles (France Boissons, Rueil-Malmaison, France) were used as reference waters during initial development and initial characterization of the method. Satisfactory extraction recoveries >70% were obtained for the majority of analytes<br><br>Evian® water: 67%	UPLCsystem (Waters, Guyancourt, France). A XevoTQ-MS®triple quadripole mass spectrometer (Waters, Guyan-court, France) equipped with an electrospray ionisation (ESI)	France<br>River water: <LOQ	2017<br>V. Brieudes
Fluoxetine	>2000 emerging contaminants	WWTP influents<br>WWTP effluent	SP+E	200 mg Oasis HLB, 150 mg Isolute ENV+, 100 mg Strata-X-AW and 100 mg Strata-X-CV	Acceptable absolute recovery rates (in the range 57–120 %) were observed for the vast majority of the studied compounds (> 75 % of the total).	UHPLC system,<br>with a HPG-3400 pump (Dionex UltiMate 3000 RSLC, Thermo Fisher Scientific, Germany), interfaced to a QTOF mass spectrometer (Maxis Impact, Bruker Daltonics, Bremen, Germany)	Greece, Athens<br>WWTP influent: 100 ng/L<br>WWTP effluent: 70 ng/L	2020<br>Pablo Gago-Ferrero
Fluoxetine	107 compounds	WWTP effluent	QuEChERS	4 g MgSO4<br>1 g NaCl<br>1 g citric acid monohydrate<br>0.5 g trisodiumcitrate dihydrate	Two concentration levels<br>93-107 % effluent	Ag ilent 1200 series HPLC system (Agilent Technologies, Foster City, CA, USA) coupled to a hybrid triple quadrupole-linear ion trap-mass spectrometer (5500 QTRAP®LC/MS/MS system, Sciex Instruments, Foster City, CA, USA).	Spain<br>WWTP effluent: n.d.	2021<br>A.B. Martínez-Piernas

Fluoxetine	80 pharmaceuticals	Coastal waters	SPE	Oasis HLB cartridges (200 mg, Waters Corp, Milford, USA)	Not mentioned	Heated electrospray (HESI) in positive or negative ion modewas used for ionization	Fiji<br>Coastal water: n.d.	2021<br>Jasha Dehm
Fluoxetine	>750 compounds	Surface water and tributaries<br><br>11 surface water samples	spe	Waters Oasis HLB SPE cartridges	Not mentioned	Ultra-high Performance Liquid Chromatography/Quadrupole-Time-of-Flight Mass Spectrometry (LC/Q-TOF-MS) analysis was performed using a semi-quantitative method on an Agilent Q-TOF (model 6545).	India<br>Surface water: n.d.	2023<br>Laura A. Richards
Fluoxetine	80 compounds	Surface water:<br>-River<br>R-eservoir	SPE	Oasis HLB SPE cartridges (Waters, 500 mg, 6 cc) (Tetracyclines, macrolides, sulfonamides, quinolones, and antiviral)<br><br>Oasis MCX SPE cartridges (Waters, 60 mg, 3 cc) (Illicit drugs)	Antiviral recovery ranged from 65.5% to 111.9%<br>Antidepressants: recovery ranged from: 78.8%–107.6%	ACQUITY liquid chromatography and a triple quadrupole mass spectrometer (Xevo T-QS micro, Waters Co., Milford, MA, USA). An electrospray ionization source (positive mode) coupling with multiple-reaction monitoring (MRM)	China<br>Surface water: <LOD–0.29 ng/L	2023<br>Miao Chen
Fluoxetine	2362 compounds	River Basin.<br>Ground Water<br>WWTP influent<br>WWTP effluent	SPE	large volume solid phase extraction (LVSPE) device. Solid phase extraction (SPE) (J2 Scientific) was performed on an Atlantic HLB-H SPE Disk	The generic sample preparation protocols that were used assure satisfactory recovery (typically above 60%) for the majority of the targeted compounds	Gas chromatography/high-resolution mass spectrometry (GC-HRMS) (DFS, Thermo) and liquid chromatography/tandem mass spectrometry (LC-MS/MS) (QTrap 5500, Sciex)	Danube<br>WWTP influent: 6.10 ng/L<br>WWTP effluent: 3.80 ng/L<br>River water: 0.43v0.84 ng/L<br>Groundwater: n.d.	2023<br>Kelsey Ng
Fluoxetine	697 compounds	Surface water (river(	SPE	HR-X (Macherey Nagel) cartridges containing 200 mg of sorbent (hydrophobic polystyrene divinylbenzene copolymer)	Not mentioned	Thermo Ultimate 3000 LC system coupled to a QExactive Plus high-resolution mass spectrometer (Thermo). Separate runs were performed in negative and positive electrospray ionization modes.	Western Kenya<br>River: n.d.	2024<br>Isaac Cheruiyot Tanui
Norfluoxetine	185 Compounds	WWTP effluents<br>Hospital<br>WWTP influent<br>WWTP effluent	-	by direct-injection LC-MS-MS<br><br>The procedure used follows the general guidelines indicated in USEPA Method 1694 except for sample preservation agent usage (USEPA, 2007)	Note:<br>Recoveries were performed with two spiking levels in: analytical control aliquot, hospital effluent, WWTP influent, WWTP effluent<br><br>Results:<br>-analytical control aliquot: 47–98%<br>-Hospital effluent:43–87%<br>-WWTP influent:58–80%<br>-WWTP effluent: 72–134%	LC/MS/MS - Agilent 1290-6460 with an electrospray ionization mode	Average concentrations<br>Hospital effluent: n.d.-30 ng/L<br>WWTP influent: n.d.<br>WWTP effluent: n.d.	2015<br>Tiago S. Oliveira
Norfluoxetine	68 compounds	Natural waters	SPE	Oasis® MCX (60 mg, 3cc) cartridge (Waters, Guyancourt, France) c	Evian® and Vittel® in glass bottles (France Boissons, Rueil-Malmaison, France) were used as reference	UPLCsystem (Waters, Guyancourt, France). A XevoTQ-MS®triple quadripole mass spectrometer (Waters, Guyan-court, France)	France<br>River water: n.d.	2017<br>V. Brieudes

					waters during initial development and initial characterization of the method. Satisfactory extraction recoveries >70'% were obtained for the majority of analytes Evian® water: 58%	equipped with an electrospray ionisation (ESI)		
Norfluoxetine	2362 compounds	River Basin.<br>Ground Water<br>WWTP influent<br>WWTP effluent	SPE	large volume solid phase extraction (LVSPE) device. Solid phase extraction (SPE) (J2 Scientific) was performed on an Atlantic HLB-H SPE Disk	The generic sample preparation protocols that were used assure satisfactory recovery (typically above 60%) for the majority of the targeted compounds	Gas chromatography/high-resolution mass spectrometry (GC-HRMS) (DFS, Thermo) and liquid chromatography/tandem mass spectrometry (LC-MS/MS) (QTrap 5500, Sciex)	Danube<br>WWTP influent: n.d.<br>WWTP effluent: n.d.<br>River water: n.d.<br>Groundwater: n.d.	2023<br>Kelsey Ng
Paroxetine	43 Pharmaceuticals	Wastewater influent<br>Wastewater effluent		SPE - Oasis 1 HLB (200 mg, 6 mL)/50 mL (pH = 7)	WWTP influents: 110%<br>WWTP effluent: 100%	Surveyor HPLC system (Thermo Finnigan) with with an electrospray ionization. The double-focusing magnetic sector HRMS (Thermo Finnigan) was operated in multiple ion detection (MID) mode for selective target analysis	Belgium, Lede<br><br>WWTP influent: n.d.<br>WWTP effluent: n.d.–<MQL	2015<br>Leendert Vergeynst
Paroxetine	185 Compounds	WWTP effluents<br>Hospital<br>WWTP influent<br>WWTP effluent	-	by direct-injection LC-MS-MS<br><br>The procedure used follows the general guidelines indicated in USEPA Method 1694 except for sample preservation agent usage (USEPA, 2007)	Note:<br>Recoveries were performed with two spiking levels in: analytical control aliquot, hospital effluent, WWTP influent, WWTP effluent<br><br>Results:<br>-analytical control aliquot: 98–100%<br>-Hospital effluent:71–155%<br>-WWTP influent:68–94%<br>-WWTP effluent: 82–94%%	LC/MS/MS - Agilent 1290-6460 with an electrospray ionization mode	Average concentrations<br>Hospital effluent: n.d.–380 ng/L<br>WWTP influent: n.d.<br>WWTP effluent: n.d.	2015<br>Tiago S. Oliveira
Paroxetine	68 compounds	Natural waters	SPE	Oasis® MCX (60 mg, 3cc) cartridge (Waters, Guyancourt, France) c	Evian® and Vittel® in glass bottles (France Boissons, Rueil-Malmaison, France) were used as reference waters during initial development and initial characterization of the method. Satisfactory extraction recoveries >70% were obtained for the majority of analytes Evian® water: 59%	UPLCsystem (Waters, Guyancourt, France). A XevoTQ-MS®triple quadripole mass spectrometer (Waters, Guyan-court, France) equipped with an electrospray ionisation (ESI)	France<br>River water: n.d.	2017<br>V. Brieudes

Paroxetine	484 chemicals	Surface waters<br>Yangtze River Delta	SPE	Not mentioned	Not mentioned	LC-HRMS, LC system coupled via a heated electrospray ion source to a quadrupole orbitrap MS (QExactive Plus, Thermo)	China<br>Surface water: ≈2–100 ng/L	2018<br>Ying Peng
Paroxetine	178 Xenobiotics	Estuary water<br>WWTP effluent<br>Ultrapure water	SPE	Strata HR-X (top), and Strata ZT-WAX and ZT-WCX (bottom) sorbents	Estuary water: 88.7%<br>WWTP effluent: 75.5%<br>Ultrapure water: 48.8%	UHPLC coupled quadrupole-Orbitrap mass spectrometer equipped with a heated ESI source (Thermo-Fisher Scientific, CA, USA)	Spain<br>Galindo Effluent: n.d.<br>Bilbao Estuary: n.d.<br>Leitzaran River: n.d.<br>Mustarre River: n.d<br>Oria River: n.d.	2021<br>González-Gaya, B
Paroxetine	107 compounds	WWTP effluent	QuEChERS	4 g MgSO4<br>1 g NaCl<br>1 g citric acid monohydrate<br>0.5 g trisodiumcitrate dihydrate	Two concentration levels<br>98-100 % effluent	Ag ilent 1200 series HPLC system (Agilent Technologies, Foster City, CA, USA) coupled to a hybrid triple quadrupole-linear ion trap-mass spectrometer (5500 QTRAP®LC/MS/MS system, Sciex Instruments, Foster City, CA, USA).	Spain<br>WWTP effluent: n.d.	2021<br>A.B. Martínez-Piernas
Paroxetine	80 pharmaceuticals	Coastal waters	SPE	Oasis HLB cartridges (200 mg, Waters Corp, Milford, USA)	Not mentioned	Heated electrospray (HESI) in positive or negative ion modewas used for ionization	Fiji<br>Coastal water: 11–370 ng/L	2021<br>Jasha Dehm
Paroxetine	>750 compounds	Surface water and tributaries<br><br>11 surface water samples	spe	Waters Oasis HLB SPE cartridges	Not mentioned	Ultra-high Performance Liquid Chromatography/Quadrupole-Time-of-Flight Mass Spectrometry (LC/Q-TOF-MS) analysis was performed using a semi-quantitative method on an Agilent Q-TOF (model 6545).	India<br>Surface water: n.d.	2023<br>Laura A. Richards
Paroxetine	80 compounds	Surface water:<br>-River<br>R-eservoir	SPE	Oasis HLB SPE cartridges (Waters, 500 mg, 6 cc) (Tetracyclines, macrolides, sulfonamides, quinolones, and antiviral)<br>Oasis MCX SPE cartridges (Waters, 60 mg, 3 cc) (Illicit drugs)	Antiviral recovery ranged from 65.5% to 111.9%<br>Antidepressants: recovery ranged from: 78.8%–107.6%	ACQUITY liquid chromatography and a triple quadrupole mass spectrometer (Xevo T-QS micro, Waters Co., Milford, MA, USA). An electrospray ionization source (positive mode) coupling with multiple-reaction monitoring (MRM)	China<br>Surface water: <LOD-0.04 ng/L	2023<br>Miao Chen
Paroxetine	database containing >40,000 chemical substances<br><br>(follow the work of 2021, González-Gaya, B, A total of 178 xenobiotics)	WWTP effluent<br>WWTP influent	SPE	500 mg solid-phase extraction (SPE) cartridges consisting of cation exchange (100 mg, ZT-WCX), anion exchange (100 mg, ZT-WAX) and reverse phase (300 mg, HRX) sorbents for effluent samples, and with 250 mg SPE cartridges containing half of the above described amounts for influent samples	Work of González-Gaya, B:<br>88.7% (Estuary water)<br>75.5 % (WWTP effluent)<br>48.8 % (ultrapure water)<br><br>2023<br>N. Lopez-Herguedas: 55%	Thermo Scientific Dionex Ulti-Mate 3000 UHPLC coupled to a Thermo Scientific Q Exactive Focus quadrupole Orbitrap mass spectrometer (UHPLC-q-Orbitrap) equipped with a heated electrospray ionization source (HESI, Thermo-Fisher Scientific, CA, USA)	Spain<br>WWTP influent: n.d.<br>WWTP effluent: n.d.	2023<br>N. Lopez-Herguedas
Paroxetine	116 Compounds	Surface water pool samples	SPE	Oasis HLB sorbent (200 mg) at the top, and a mixture of the Bond Elut PPL (150 mg), WAX (100 mg), and WCX (100 mg) sorbents at the bottom	Pool samples: 17.14-35.70%<br><br>Good recoveries were obtained between 70 and 120% for the majority of the selected contaminants.	ACQUITY UHPLC system (Waters, Milford, MA) coupled with a Q-Exactive Orbitrap mass spectrometer (Thermo-Fisher Scientific, Germany) and equipped with heated electrospray ionization (HESI)	Spain<br>No information for each specific compound could be taken<br><br>In general, very low levels of contamination were found.<br>48 compounds of the 116 targeted compounds were detected in at least one of the samples	2023<br>Olga Gómez-Navarro

							The highest concentrations found were mainly concerning those compounds used frequently in our day-to-day life such as sucralose (sweetener) with the highest concentration of 376.9 ng/Lfound in Taradell followed by caffeine (stimulant), acesulfame or saccharin (sweeteners) with maximum concentrations of 179.4, 130.3 and 119.0 ng/L	
Paroxetine	2362 compounds	River Basin.<br>Ground Water<br>WWTP influent<br>WWTP effluent	SPE	large volume solid phase extraction (LVSPE) device. Solid phase extraction (SPE) (J2 Scientific) was performed on an Atlantic HLB-H SPE Disk	The generic sample preparation protocols that were used assure satisfactory recovery (typically above 60%) for the majority of the targeted compounds	Gas chromatography/high-resolution mass spectrometry (GC-HRMS) (DFS, Thermo) and liquid chromatography/tandem mass spectrometry (LC-MS/MS) (QTrap 5500, Sciex)	Danube<br>WWTP influent: n.d.<br>WWTP effluent: n.d.<br>River water: n.d.<br>Groundwater: n.d.	2023<br>Kelsey Ng
Sertraline	185 Compounds	WWTP effluents<br>Hospital<br>WWTP influent<br>WWTP effluent	-	by direct-injection LC-MS-MS<br><br>The procedure used follows the general guidelines indicated in USEPA Method 1694 except for sample preservation agent usage (USEPA, 2007)	Note:<br>Recoveries were performed with two spiking levels in: analytical control aliquot, hospital effluent, WWTP influent, WWTP effluent<br><br>Results:<br>-analytical control aliquot:100-102%<br>-Hospital effluent:83–109%<br>-WWTP influent:89–96%<br>-WWTP effluent: 86–115%	LC/MS/MS - Agilent 1290-6460 with an electrospray ionization mode	Average concentrations<br>Hospital effluent: 20–150 ng/L<br>WWTP influent: 40–140 ng/L<br>WWTP effluent: n.d.–80 ng/L	2015<br>Tiago S. Oliveira
Sertraline	68 compounds	Natural waters	SPE	Oasis® MCX (60 mg, 3cc) cartridge (Waters, Guyancourt, France) c	Evian® and Vittel® in glass bottles (France Boissons, Rueil-Malmaison, France) were used as reference waters during initial development and initial characterization of the method. Satisfactory extraction recoveries >70% were obtained for the majority of analytes 50%	UPLCsystem (Waters, Guyancourt, France). A XevoTQ-MS®triple quadripole mass spectrometer (Waters, Guyan-court, France) equipped with an electrospray ionisation (ESI)	France<br>River water: n.d.	2017<br>V. Brieudes
**Sertraline**	484 chemicals	Surface waters<br>Yangtze River Delta	SPE	Not mentioned	Not mentioned	LC-HRMS, LC system coupled via a heated electrospray ion source to a quadrupole orbitrap MS (QExactive Plus, Thermo)	China<br>Surface water: ≈2–100 ng/L	2018<br>Ying Peng

Sertraline	>2000 emerging contaminants	WWTP influents<br>WWTP effluent	SP+E	200 mg Oasis HLB, 150 mg Isolute ENV+, 100 mg Strata-X-AW and 100 mg Strata-X-CV	Acceptable absolute recovery rates (in the range 57–120 %) were observed for the vast majority of the studied compounds (> 75 % of the total)	UHPLC system,<br>with a HPG-3400 pump (Dionex UltiMate 3000 RSLC, Thermo Fisher Scientific, Germany), interfaced to a QTOF mass spectrometer (Maxis Impact, Bruker Daltonics, Bremen, Germany)	Greece, Athens<br>WWTP influent: 90 ng/L<br>WWTP effluent: 30 ng/L	2020<br>Pablo Gago-Ferrero
Sertraline	80 pharmaceuticals	Coastal waters	SPE	Oasis HLB cartridges (200 mg, Waters Corp, Milford, USA)	Not mentioned	Heated electrospray (HESI) in positive or negative ion modewas used for ionization	Fiji<br>Coastal water: 12-140 ng/L	2021<br>Jasha Dehm
Sertraline	178 Xenobiotics	Estuary water<br>WWTP effluent<br>Ultrapure water	SPE	Strata HR-X (top), and Strata ZT-WAX and ZT-WCX (bottom) sorbents	Estuary: 97.8%<br>WWTP Effluent: 94.4%<br>Ultrapure: 45.7%	UHPLC coupled quadrupole-Orbitrap mass spectrometer equipped with a heated ESI source (Thermo-Fisher Scientific, CA, USA)	Spain<br>Galindo Effluent: 5.6 ng/L<br>Bilbao Estuary: n.d.<br>Leitzaran River: n.d.<br>Mustarre River: n.d.<br>Oria River: n.d.	2021<br>González-Gaya, B,
Sertraline	>750 compounds	Surface water and tributaries<br><br>11 surface water samples	spe	Waters Oasis HLB SPE cartridges	Not mentioned	Ultra-high Performance Liquid Chromatography/Quadrupole-Time-of-Flight Mass Spectrometry (LC/Q-TOF-MS) analysis was performed using a semi-quantitative method on an Agilent Q-TOF (model 6545).	India<br>Surface water: n.d.	2023<br>Laura A. Richards
Sertraline	80 compounds	Surface water:<br>-River<br>Reservoir	SPE	Oasis HLB SPE cartridges (Waters, 500 mg, 6 cc) (Tetracyclines, macrolides, sulfonamides, quinolones, and antiviral)<br>Oasis MCX SPE cartridges (Waters, 60 mg, 3 cc) (Illicit drugs)	Antiviral recovery ranged from 65.5% to 111.9%<br>Antidepressants: recovery ranged from: 78.8%−107.6%	ACQUITY liquid chromatography and a triple quadrupole mass spectrometer (Xevo T-QS micro, Waters Co., Milford, MA, USA). An electrospray ionization source (positive mode) coupling with multiple-reaction monitoring (MRM)	China<br>Surface water: <LOD-0.55 ng/L	2023<br>Miao Chen
Sertraline	database containing >40,000 chemical substances<br><br>(follow the work of 2021, González-Gaya, B, A total of 178 xenobiotics)	WWTP effluent<br>WWTP influent	SPE	500 mg solid-phase extraction (SPE) cartridges consisting of cation exchange (100 mg, ZT-WCX), anion exchange (100 mg, ZT-WAX) and reverse phase (300 mg, HRX) sorbents for effluent samples, and with 250 mg SPE cartridges containing half of the above described amounts for influent samples	Work of González-Gaya, B:<br>97.87% (Estuary water)<br>94.4 % (WWTP effluent)<br>45.7 % (ultrapure water)<br><br>N. Lopez-Herguedas: 97%	Thermo Scientific Dionex Ulti-Mate 3000 UHPLC coupled to a Thermo Scientific Q Exactive Focus quadrupole Orbitrap mass spectrometer (UHPLC-q-Orbitrap) equipped with a heated electrospray ionization source (HESI, Thermo-Fisher Scientific, CA, USA)	Spain<br>WWTP influent: 4 ng/L<br>WWTP effluent: 7–17 ng/L	2023<br>N. Lopez-Herguedas,
Sertraline	116 Compounds	Surface water pool samples	SPE	Oasis HLB sorbent (200 mg) at the top, and a mixture of the Bond Elut PPL (150 mg), WAX (100 mg), and WCX (100 mg) sorbents at the bottom	Pool samples: 27.20–37.53%<br><br>Good recoveries were obtained between 70 and 120% for the majority of the selected contaminants.	ACQUITY UHPLC system (Waters, Milford, MA) coupled with a Q-Exactive Orbitrap mass spectrometer (Thermo-Fisher Scientific, Germany) and equipped with heated electrospray ionization (HESI)	Spain<br>No information for each specific compound could be taken<br><br>In general, very low levels of contamination were found.<br>48 compounds of the 116 targeted compounds were detected in at least one of the samples<br>The highest concentrations found were mainly concerning those compounds used frequently in our	2023<br>Olga Gómez-Navarro

							day-to-day life such as sucralose (sweetener) with the highest concentration of 376.9 ng/Lfound in Taradell followed by caffeine (stimulant), acesulfame or saccharin (sweeteners) with maximum concentrations of 179.4, 130.3 and 119.0 ng/L	
Sertraline	2362 compounds	River Basin.<br>Ground Water<br>WWTP influent<br>WWTP effluent	SPE	large volume solid phase extraction (LVSPE) device. Solid phase extraction (SPE) (J2 Scientific) was performed on an Atlantic HLB-H SPE Disk	The generic sample preparation protocols that were used assure satisfactory recovery (typically above 60%) for the majority of the targeted compounds	Gas chromatography/high-resolution mass spectrometry (GC-HRMS) (DFS, Thermo) and liquid chromatography/tandem mass spectrometry (LC-MS/MS) (QTrap 5500, Sciex)	Danube<br>WWTP influent: 0.53 ng/L<br>WWTP effluent: n.d.<br>River water: <LOQ–1.12 ng/L<br>Groundwater: n.d.	2023<br>Kelsey Ng
Sertraline	697 compounds	Surface water (river(	SPE	HR-X (Macherey Nagel) cartridges containing 200 mg of sorbent (hydrophobic polystyrene divinylbenzene copolymer)	Not mentioned	Thermo Ultimate 3000 LC system coupled to a QExactive Plus high-resolution mass spectrometer (Thermo). Separate runs were performed in negative and positive electrospray ionization modes.	Western Kenya<br>River water: n.d.	2024<br>Isaac Cheruiyot Tanui
Venlafaxine	43 Pharmaceuticals	Wastewater influent<br>Wastewater effluent		SPE - Oasis 1 HLB (200 mg, 6 mL)/50 mL (pH = 7)	WWTP influent: 110%<br>WWTP effluent: 100%	Surveyor HPLC system (Thermo Finnigan) with with an electrospray ionization. The double-focusing magnetic sector HRMS (Thermo Finnigan) was operated in multiple ion detection (MID) mode for selective target analysis	Belgium, Lede<br><br>WWTP influent: 219–403 ng/L<br>WWTP effluent: 205–365 ng/L	2015<br>Leendert Vergeynst
Venlafaxine	185 Compounds	WWTP effluents<br>Hospital<br>WWTP influent<br>WWTP effluent	-	by direct-injection LC-MS-MS<br><br><br>The procedure used follows the general guidelines indicated in USEPA Method 1694 except for sample preservation agent usage (USEPA, 2007)	Note:<br>Recoveries were performed with two spiking levels in: analytical control aliquot, hospital effluent, WWTP influent, WWTP effluent<br><br>Results:<br>-analytical control aliquot:104–108%<br>-Hospital effluent:83–135%<br>-WWTP influent:80–114%<br>-WWTP effluent: 40–112%%	LC/MS/MS - Agilent 1290-6460 with an electrospray ionization mode	Average concentrations<br>Hospital effluent: 170–660 ng/L<br>WWTP influent: 330–480 ng/L<br>WWTP effluent: 50–550 ng/L	2015<br>Tiago S. Oliveira
Venlafaxine	450 Compounds	WWTP efluent	SPE	Strata X (200 mg) and a mixture of Strata WAX (100 mg), Strata WCX (100 mg), and IsoluteENV+ (150 mg), to achieve sufficient enrichment for a broad range of compounds (neutral, acidic, basic).	Not mentioned	UHPLC system (DionexUltiMate 3000 RSLC, Thermo Fish-erScientific, Germany) interfaced to a QTOF mass spectrometer(MaxisImpact, Bruker Daltonics, Bremen, Germany)<br>UPLC system (Waters, Milford, MA, USA) was interfaced to a hybrid quadrupole-orthogonal	Greece, Athens<br>Detected in all samples (7 samples)	2017<br>Ibanez

						acceleration-TOFmass spectrometer (XEVO G2 QTOF, Waters Micromass, Manch-ester, UK), using an orthogonal Z-spray-ESI interface		
Venlafaxine	68 compounds	Natural waters	SPE	Oasis® MCX (60 mg, 3cc) cartridge (Waters, Guyancourt, France) c	Evian® and Vittel® in glass bottles (France Boissons, Rueil-Malmaison, France) were used as reference waters during initial development and initial characterization of the method. Satisfactory extraction recoveries >70% were obtained for the majority of analytes 93%	UPLCsystem (Waters, Guyancourt, France). A XevoTQ-MS®triple quadripole mass spectrometer (Waters, Guyan-court, France) equipped with an electrospray ionisation (ESI)	France<br>River water: n.d.	2017<br>V. Brieudes
Venlafaxine	>2000 emerging contaminants	WWTP influents<br>WWTP effluent	SP+E	200 mg Oasis HLB, 150 mg Isolute ENV+, 100 mg Strata-X-AW and 100 mg Strata-X-CV	Acceptable absolute recovery rates (in the range 57–120 %) were observed for the vast majority of the studied compounds (> 75 % of the total).	UHPLC system,<br>with a HPG-3400 pump (Dionex UltiMate 3000 RSLC, Thermo Fisher Scientific, Germany), interfaced to a QTOF mass spectrometer (Maxis Impact, Bruker Daltonics, Bremen, Germany)	Greece, Athens<br>WWTP influent: 920 ng/L<br>WWTP effluent: 1,000 ng/L	2020<br>Pablo Gago-Ferrero
Venlafaxine	80 pharmaceuticals	Coastal waters	SPE	Oasis HLB cartridges (200 mg, Waters Corp, Milford, USA)	Not mentioned	Heated electrospray (HESI) in positive or negative ion modewas used for ionization	Fiji<br>Coastal water: 5.7–8.4 ng/L	2021<br>Jasha Dehm
Venlafaxine	107 compounds	WWTP effluent	QuEChERS	4 g MgSO4<br>1 g NaCl<br>1 g citric acid monohydrate<br>0.5 g trisodiumcitrate dihydrate	Two concentration levels<br>74-111 % effluent	Ag ilent 1200 series HPLC system (Agilent Technologies, Foster City, CA, USA) coupled to a hybrid triple quadrupole-linear ion trap-mass spectrometer (5500 QTRAP®LC/MS/MS system, Sciex Instruments, Foster City, CA, USA).	Spain<br>WWTP effluent: 172–382 ng/L	2021<br>A.B. Martínez-Piernas
Venlafaxine	79 compounds	Groundwater<br>Surface water<br>WWTP effluents	SPE<br>ONLINE	Mixed-mode online solid-phase extraction. A series of sorbents, including C18 substances, hyper cross-linked polymers, cation-exchange resin, anion-exchange resin, and graphitized nonporous carbon, were selected and mixed into a single online cartridge	Ground water: 97%<br>Surface water: 92%<br>WWTP effluent: 93%	Two sets of MS, QQQ MS and QTOF MS, were applied in this study.<br>Both MS systems were equipped with an Agilent Jet Stream (AJS) electrospray ionization (ESI) source.	India<br>Ground water: 0.10 ng/L<br>Surface water: 0.47–20 ng/L<br>WWTP effluent: 240–880 ng/L	2022<br>Jianmin Zou
venlafaxine	60 compounds	WWTP influent	SPE	Tandem hydrophilic-lipophilic balance (HLB) and mixed-mode cation exchange (MCX) sorbents (Waters)	NanoPure: 72%<br><br>WWTP influent: 78%	Agilent 6410 triple quadrupole mass analyzer equipped with a 1200 HPLC system (Palo Alto, CA) coupled with electrospray ionization (ESI).	United States<br>WWTP influent: n.d.-1,450 ng/L	2023<br>Lahiruni M. Halwatura
venlafaxine	>750 compounds	Surface water and tributaries	spe	Waters Oasis HLB SPE cartridges	Not mentioned	Ultra-high Performance Liquid Chromatography/Quadrupole-Time-of-Flight Mass Spectrometry (LC/Q-TOF-MS) analysis was	India<br>Surface water: n.d.	2023<br>Laura A. Richards

		11 surface water samples				performed using a semi-quantitative method on an Agilent Q-TOF (model 6545).		
Venlafaxine	80 compounds	Surface water:<br>-River<br>Reservoir	SPE	Oasis HLB SPE cartridges (Waters, 500 mg, 6 cc) (Tetracyclines, macrolides, sulfonamides, quinolones, and antiviral)<br><br>Oasis MCX SPE cartridges (Waters, 60 mg, 3 cc) (Illicit drugs)	Antiviral recovery ranged from 65.5% to 111.9%<br>Antidepressants: recovery ranged from: 78.8%–107.6%	ACQUITY liquid chromatography and a triple quadrupole mass spectrometer (Xevo T-QS micro, Waters Co., Milford, MA, USA). An electrospray ionization source (positive mode) coupling with multiple-reaction monitoring (MRM)	China<br>Surface water: <LOD-5.35 ng/L	2023<br>Miao Chen
Venlafaxine	116 Compounds	Surface water pool samples	SPE	Oasis HLB sorbent (200 mg) at the top, and a mixture of the Bond Elut PPL (150 mg), WAX (100 mg), and WCX (100 mg) sorbents at the bottom	Pool samples: 65.98–-75.43%<br><br>Good recoveries were obtained between 70 and 120% for the majority of the selected contaminants.	ACQUITY UHPLC system (Waters, Milford, MA) coupled with a Q-Exactive Orbitrap mass spectrometer (Thermo-Fisher Scientific, Germany) and equipped with heated electrospray ionization (HESI)	Spain<br>No information for each specific compound could be taken<br><br>In general, very low levels of contamination were found.<br>48 compounds of the 116 targeted compounds were detected in at least one of the samples<br>The highest concentrations found were mainly concerning those compounds used frequently in our day-to-day life such as sucralose (sweetener) with the highest concentration of 376.9 ng/Lfound in Taradell followed by caffeine (stimulant), acesulfame or saccharin (sweeteners) with maximum concentrations of 179.4, 130.3 and 119.0 ng/L	2023<br>Olga Gómez-Navarro
Venlafaxine	2362 compounds	River Basin.<br>Ground Water<br>WWTP influent<br>WWTP effluent	SPE	large volume solid phase extraction (LVSPE) device. Solid phase extraction (SPE) (J2 Scientific) was performed on an Atlantic HLB-H SPE Disk	The generic sample preparation protocols that were used assure satisfactory recovery (typically above 60%) for the majority of the targeted compounds	Gas chromatography/high-resolution mass spectrometry (GC-HRMS) (DFS, Thermo) and liquid chromatography/tandem mass spectrometry (LC-MS/MS) (QTrap 5500, Sciex)	Danube<br>WWTP influent: 0.36–1.70 ng/L<br>WWTP effluent: 0.21-0.22 ng/L<br>River water: <LOQ-15.67 ng/L<br>Groundwater: n.d.	2023<br>Kelsey Ng
Venlafaxine	32 compounds	Surface Water<br>WWTP effluent	SPE	Method developed by Olga Gómez-Navarro, 2023:<br>Oasis HLB sorbent (200 mg) at the top, and a mixture of the Bond Elut PPL (150 mg), WAX (100 mg), and WCX (100 mg) sorbents at the bottom	Method developed by Olga Gómez-Navarro, 2023:<br><br>Good recoveries were obtained between 70 and 120% for the majority of the selected contaminants.	UPLC system (Waters. Milford. MA),<br>coupled with an Orbitrap Q-Exactive™ mass spectrometer (Thermo Fischer Scientific, San Jose, CA, USA).	Surface water: 80.5–758 ng/L<br>WWTP effluent 855 ng/L	2024<br>Diana P. Manjarrés-Lopez
Trazodone	185 Compounds	WWTP effluents<br>Hospital<br>WWTP influent<br>WWTP effluent	-	by direct-injection LC-MS-MS<br><br><br>The procedure used follows the general guidelines indicated in USEPA Method 1694	Note:<br>Recoveries were performed with two spiking levels in: analytical control aliquot, hospital effluent, WWTP	LC/MS/MS - Agilent 1290-6460 with an electrospray ionization mode	Average concentrations<br>Hospital effluent: 30–1,160 ng/L<br>WWTP influent: 20–80 ng/L<br>WWTP effluent: n.d.–40 ng/L	2015<br>Tiago S. Oliveira

				except for sample preservation agent usage (USEPA, 2007)	influent, WWTP effluent<br>Results:<br>-analytical control aliquot:105–106%<br>-Hospital effluent:111–119%<br>-WWTP influent:105–125%<br>-WWTP effluent: 108–114%%			
Trazodone	107 compounds	WWTP effluent	QuEChERS	4 g $MgSO_4$<br>1 g NaCl<br>1 g citric acid monohydrate<br>0.5 g trisodiumcitrate dihydrate	Two concentration levels<br>66-83 % effluent	Ag ilent 1200 series HPLC system (Agilent Technologies, Foster City, CA, USA) coupled to a hybrid triple quadrupole-linear ion trap-mass spectrometer (5500 QTRAP®LC/MS/MS system, Sciex Instruments, Foster City, CA, USA).	Spain<br>WWTP effluent: 27–58 ng/L	2021<br>A.B. Martínez-Piernas
Trazodone	80 compounds	Surface water:<br>-River<br>Reservoir	SPE	Oasis HLB SPE cartridges (Waters, 500 mg, 6 cc) (Tetracyclines, macrolides, sulfonamides, quinolones, and antiviral)<br><br>Oasis MCX SPE cartridges (Waters, 60 mg, 3 cc) (Illicit drugs)	Antiviral recovery ranged from 65.5% to 111.9%<br>Antidepressants: recovery ranged from: 78.8%–107.6%	ACQUITY liquid chromatography and a triple quadrupole mass spectrometer (Xevo T-QS micro, Waters Co., Milford, MA, USA). An electrospray ionization source (positive mode) coupling with multiple-reaction monitoring (MRM)	China<br>Surface water: <LOD—0.08 ng/L	2023<br>Miao Chen
Trazodone	2362 compounds	River Basin.<br>Ground Water<br>WWTP influent<br>WWTP effluent	SPE	large volume solid phase extraction (LVSPE) device. Solid phase extraction (SPE) (J2 Scientific) was performed on an Atlantic HLB-H SPE Disk	The generic sample preparation protocols that were used assure satisfactory recovery (typically above 60%) for the majority of the targeted compounds	Gas chromatography/high-resolution mass spectrometry (GC-HRMS) (DFS, Thermo) and liquid chromatography/tandem mass spectrometry (LC-MS/MS) (QTrap 5500, Sciex)	Danube<br>WWTP influent: n.d.<br>WWTP effluent: n.d.<br>River water: n.d.<br>Groundwater: n.d.	2023<br>Kelsey Ng

A)

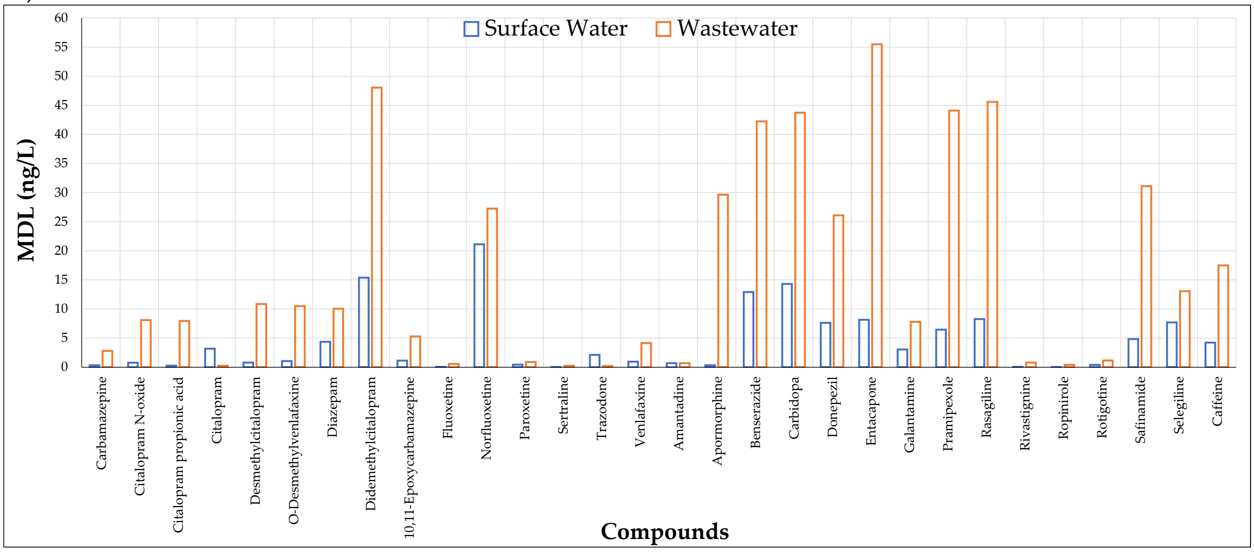

B)

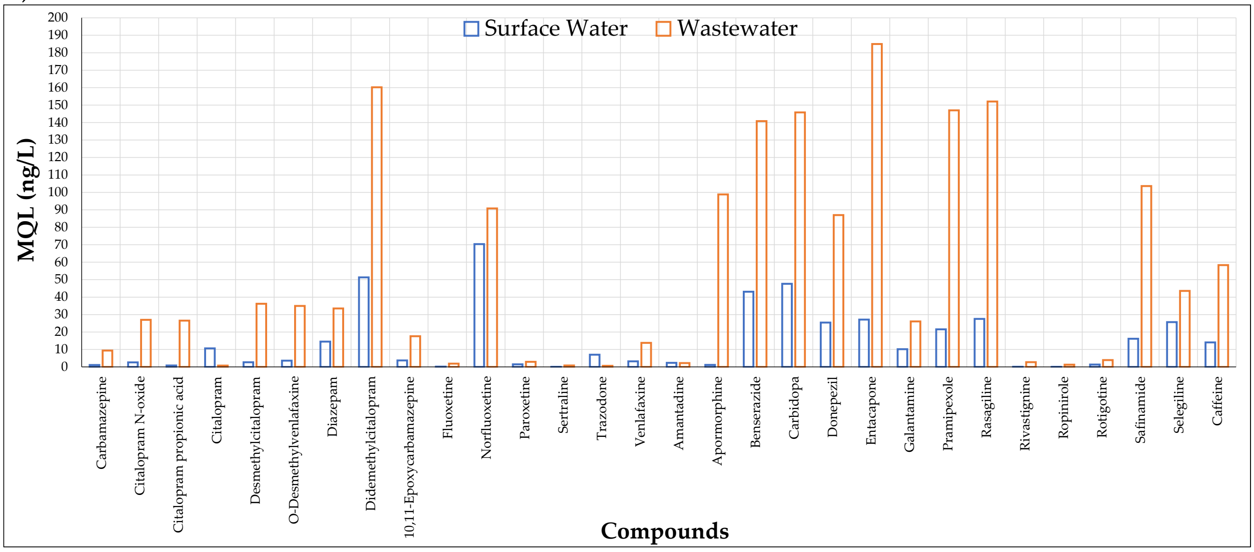

Figure S1. Method detection limits for surface water and wastewater matrices for each studied compound.

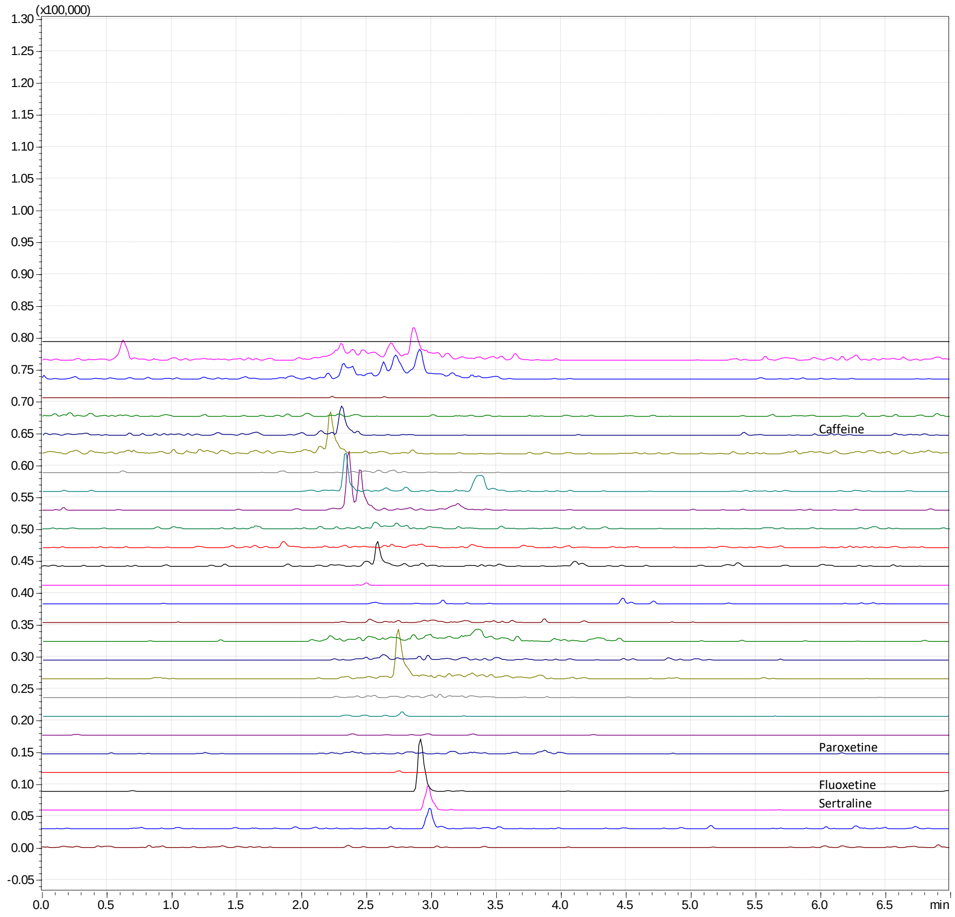

Figure S2. Overlay chromatogram of the detected compounds in ocean sample AO2.

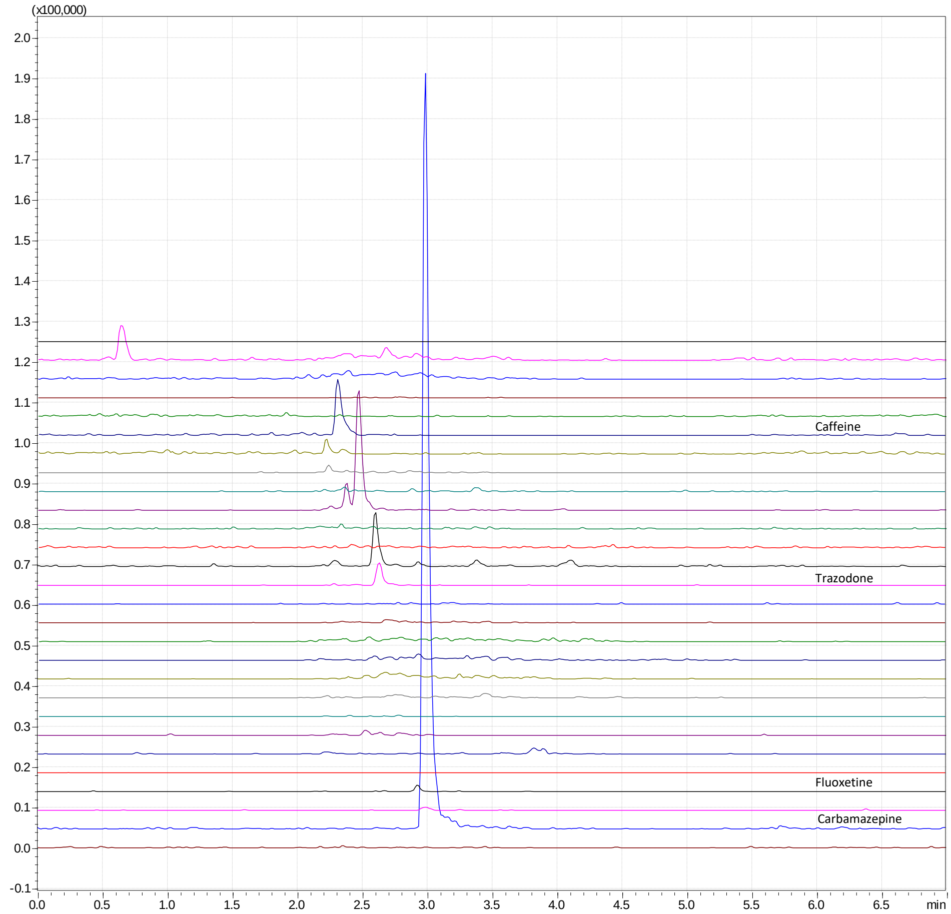

Figure S3. Overlay chromatogram of the detected compounds in stream water sample S3.

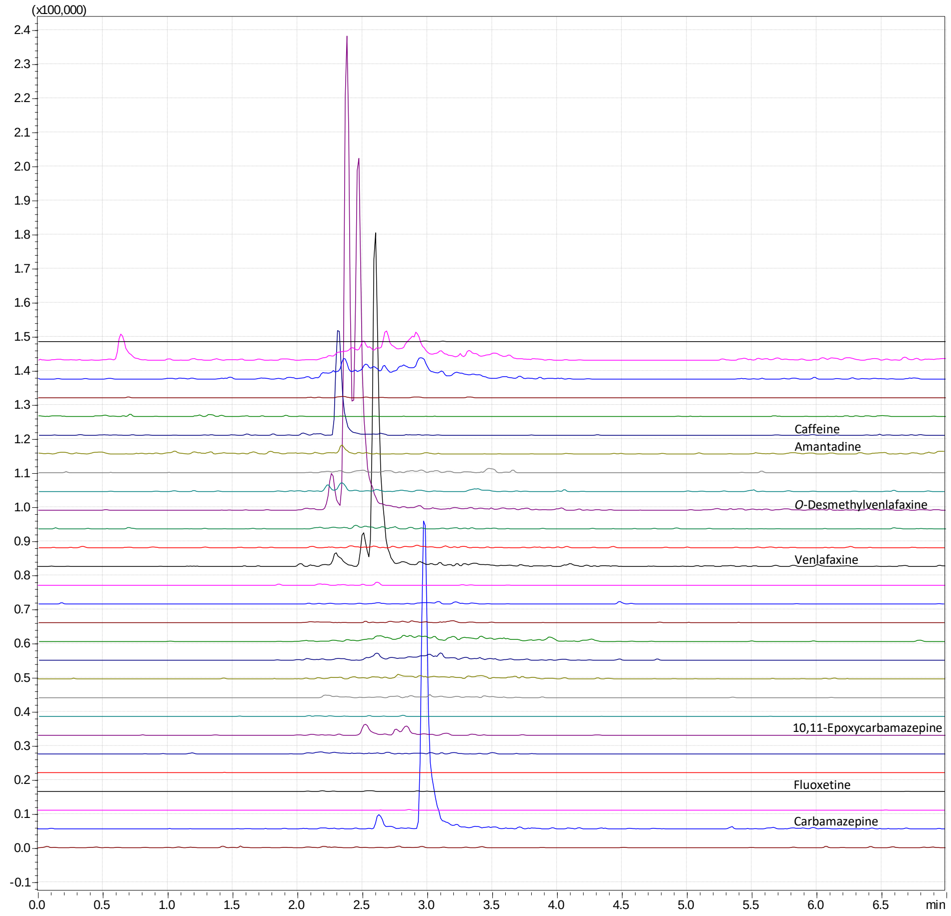

Figure S4. Overlay chromatogram of the detected compounds in river water sample R1.

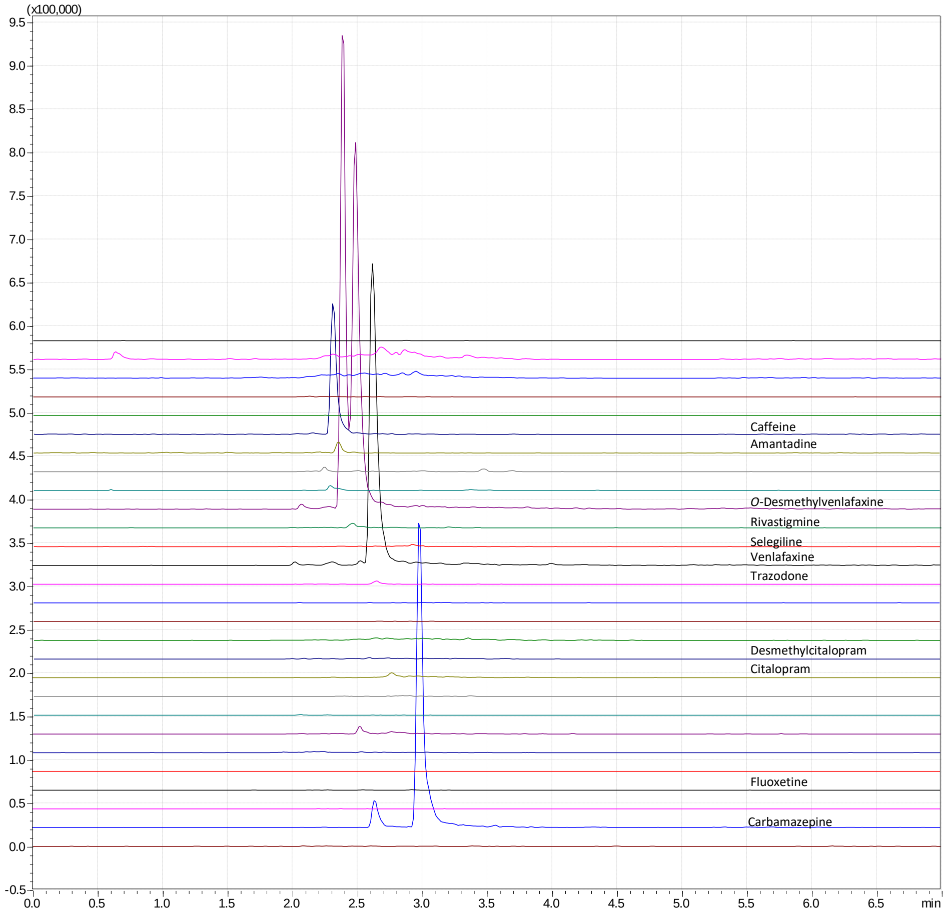

Figure S5. Overlay chromatogram of the detected compounds in WWTP effluent wastewater sample E1.

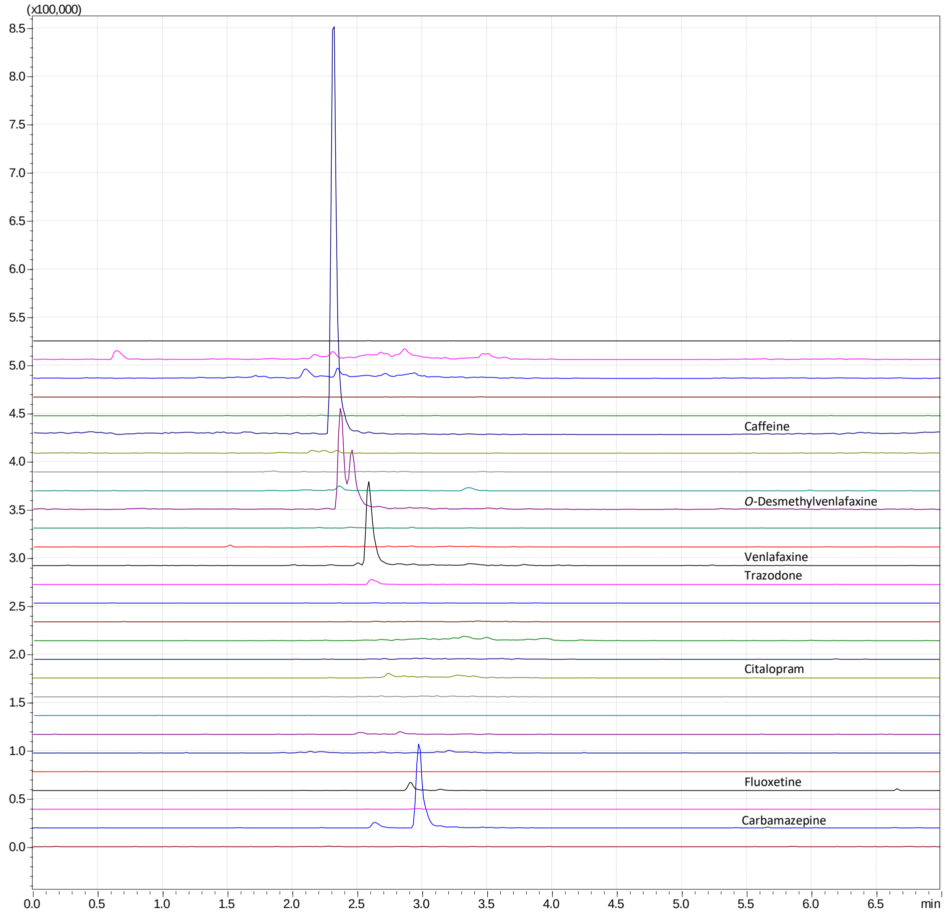

Figure S6. Overlay chromatogram of the detected compounds in WWTP influent wastewater sample I2.
